# Application of novel nanomagnetic metal–organic frameworks as a catalyst for the synthesis of new pyridines and 1,4-dihydropyridines via a cooperative vinylogous anomeric based oxidation

**DOI:** 10.1038/s41598-021-84005-2

**Published:** 2021-03-05

**Authors:** Hassan Sepehrmansourie, Mahmoud Zarei, Mohammad Ali Zolfigol, Saeed Babaee, Sadegh Rostamnia

**Affiliations:** 1 Department of Organic Chemistry, Faculty of Chemistry, Bu-Ali Sina University, 6517838683 Hamedan, Iran; 2 Organic and Nano Group (ONG), Department of Chemistry, Iran University of Science and Technology (IUST), PO Box, 16846-13114 Tehran, Iran; 3 Organic and Nano Group (ONG), Department of Chemistry, Faculty of Science, University of Maragheh, PO Box, 55181-83111 Maragheh, Iran

**Keywords:** Chemistry, Nanoscience and technology

## Abstract

Herein, a new magnetic metal–organic frameworks based on Fe_3_O_4_ (NMMOFs) with porous and high surface area materials were synthesized. Then, NMMOFs were characterized by FT-IR, XRD, SEM, elemental mapping, energy dispersive X-ray (EDS), TG, DTG, VSM, and N_2_ adsorption–desorption isotherms (BET). Fe_3_O_4_@Co(BDC)-NH_2_ as a magnetic porous catalyst was applied for synthesis of novel fused pyridines and 1,4-dihydropyridines with pyrazole and pyrimidine moieties as suitable drug candidates under ultrasonic irradiation. The significant advantages of the presented methodology are mild, facile workup, high yields, short reaction times, high thermal stability, and reusability of the described NMMOFs catalyst.

## Introduction

Catalysis under ultrasonic irradiation has been widely applied for the preparation of organic compounds and catalysis^1–3^. On the other hand, hybrid organic–inorganic catalysts such as metal–organic frameworks (MOFs) as a new class of porous materials have high attention in chemical processes. Porous and magnetic materials have been widely used in biotechnology, magnetic resonance imaging (MRI), catalysis, adsorption, gas separation, and purification, optics, drug delivery, etc.^4–6^. However, metal–organic frameworks (MOFs) are a widespread strategy for the expansion of new porous materials to reach with the higher surface area. By selecting a suitable plan, reactants and reaction conditions can be correctly controlled by the porosity and structure of desired materials^7–13^. Magnetic catalysts have been used for the synthesis of a good range of pharmaceutical and chemical compounds, due to their easy removal and convenient separation^14–16^. The reported catalysts can be easily isolated from the reaction mixture with an external magnetic field^17–19^. Therefore magnetic metal–organic frameworks (MMOFs) have been used for various purposes due to their exciting properties^20–23^, such as high thermal stability and application at the hard reaction conditions^24–26^. The chemistry of magnetic metal–organic frameworks and their corresponding applications comprehensively have been reviewed^27–29^.

**Figure 1:**
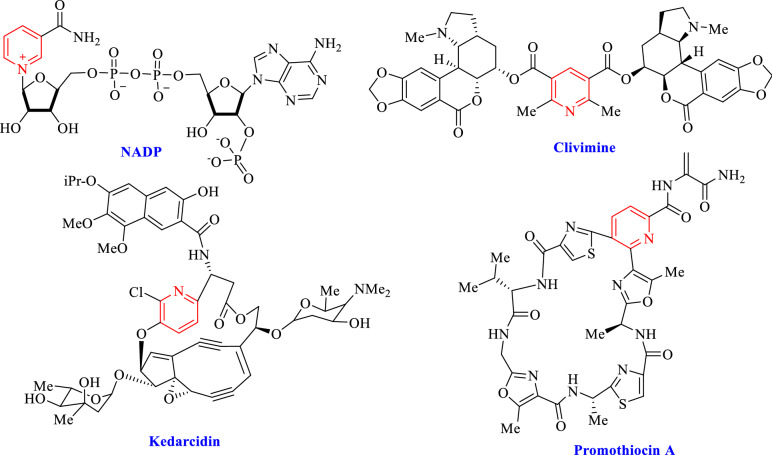
Structure of pyridine as natural products.

Fused *N*-heterocycles compounds with pyrazole and pyridines have shown a broad spectrum of biological and agricultural activities such as antitumor, cardiotonic hepatoprotactive, antihypertensive, antibronchitic, and antifungal activity^30–33^. Therefore, research and develop the new strategy are necessary for the synthesis of pyridines with pyrazole moieties. Pyridine derivatives are the central core of natural products such as NADP, clivimine, kedarcidin, and promothiocin A (Fig. 1)^34^.

**Scheme 1:**
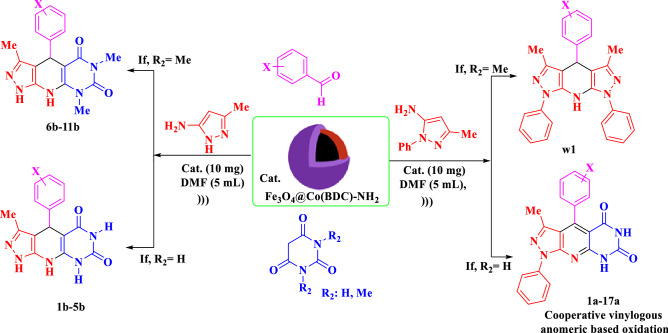
Preparation of novel fused pyridines and 1,4-dihydropyridines with pyrazole and pyrimidine moieties by using Fe_3_O_4_@Co(BDC)-NH_2_ as the catalyst.

Synthesis of composites of MOF and nano-magnetic Fe_3_O_4_ is our great research interest. With this aim, we have decided to synthesize nano-magnetic metal–organic frameworks Fe_3_O_4_@Co(BDC)-NH_2_ as a porous and magnetic catalyst under ultrasonic irradiation condition. This nanomagnetic metal–organic frameworks (NMMOFs) was applied in the synthesis of novel fused pyridines and 1,4-dihydropyridines with pyrazole and pyrimidine moieties by using the corresponding precursors in DMF (5 mL) as solvent under ultrasonic irradiation (Scheme 1).

## Experimental

### Materials and methods

All chemicals were purchased from Merck Chemical Company. The known products were identified by comparison of their melting points and spectral data with those reported in the literature. To scrutinize the progress of the reaction silica gel SIL G/UV 254 plates were used. From the model of the BRUKER Ultra shield, NMR spectrometer (δ in ppm) was recorded ^1^H NMR (400 MHz) and ^13^C NMR (100 MHz). Recorded on a Büchi B-545 apparatus in open capillary tubes were melting points. The PerkinElmer PE-1600-FTIR device was registered for the infrared spectra of compounds. SEM was performed using a scanning electron microscope for field publishing made by TE-SCAN. Thermal gravimetry (TG), differential thermal gravimetric (DTG) and differential thermal (DTA) were analyzed by a Perkin Elmer (Model: Pyris 1). The analysis 25–1000 °C, temperature increase rate of 10 °C min^−1^.

### General procedure for the synthesis of Fe_3_O_4_@CH_2_CO_2_H

Fe_3_O_4_ was prepared according to the previously reported literature^35,36^. Then, in a 25 mL round-bottomed flask, a mixture of Fe_3_O_4_ (1 g), HSCH_2_CO_2_H (10.0 mmol, 1.38 g), and EtOH (30 mL) were added and refluxed for 24 h. After this time, a dark brown precipitate was appeared, which it is isolated by using a magnet. The obtained Fe_3_O_4_@CH_2_CO_2_H (1.95 g) was dried under vacuum^23^.

### General procedure for the synthesis of Fe_3_O_4_@Co(BDC)-NH_2_

**Scheme 2:**
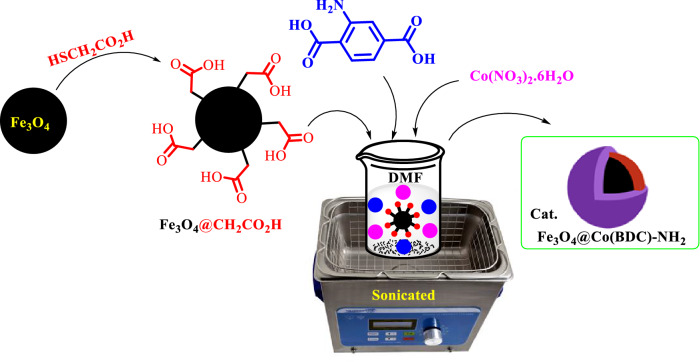
Synthesis of Fe_3_O_4_@Co(BDC)-NH_2_ as a nanomagnetic metal–organic frameworks.

At first, a solution of 45.0 mM of Co(NO_3_)_2_·6H_2_O (2.34 g in 180 mL DMF) (solution I) and 45.0 mM of H_2_BDC-NH_2_ (1.46 g in 180 mL DMF) (solution II) were prepared respectively. In a 25 mL glass vials, a mixture of Fe_3_O_4_@CH_2_CO_2_H (0.5 g) and 10 mL of solution I were sonicated for 20 min. Then, this mixture was separated by a permanent magnet and washed with DMF as step I. Then, a mixture of step I and 10 mL of solution II were sonicated for 45 min. The produced solid was separated by a permanent magnet and washed with EtOH as step II. In continued, two strategies (a mixture of step I and step II) were repeated 18 times, respectively. Finally, Fe_3_O_4_@Co(BDC)-NH_2_ (0.8 g) was dried under vacuum for 2 h (Scheme 2).

### General procedure for the synthesis of novel fused pyridines and 1,4-dihydropyridines

In a 10 mL round-bottomed, a mixture of aldehyde (1.0 mmol), pyrimidine (1,3-dimethylpyrimidine-2,4,6(1*H*,3*H*,5*H*)-trione or pyrimidine-2,4,6(1*H*,3*H*,5*H*)-trione) and pyrazole-5-amine (3-methyl-1*H*-pyrazole-5-amine or 3-methyl-1-phenyl-1*H*-pyrazole-5-amine) derivatives] and Fe_3_O_4_@Co(BDC)-NH_2_ (10 mg) as a catalyst were mixed in DMF (5 mL) as solvent under ultrasonic irradiation. After completion of the reaction (monitor by TLC *n-*hexane/ethyl acetate; 4:6), the catalyst was separated by an external magnet. Finally, the mixture was poured into H_2_O and filtered off its precipitate. The obtained residue was washed with warm ethanol and dried at 100 °C (Scheme 1).

## Result and discussion

The systematic study of the stereoelectronic effects in target molecules, allows for the design of synthetic strategies based on a nomerically driven stereoselective reactions, or highly biased equilibria among isomeric products. To the best of our knowledge, many biological processes involve the oxidation–reduction of substrates by NAD^+^/NADH, respectively^37–41^. The key feature of the oxidation mechanism is hydride transfer from carbon via stereoelectronic interactions. Thus the development of stereoelectronic effects leads to knowledge-based designing of biomimetic reactions. The obtained results from this research will be supporting the idea of rational designs, syntheses, and applications of tasked-specific catalysts and molecules for the development of stereoelectronic effects in the course of organic synthesis. With this aim, a nanomagnetic metal–organic frameworks (NMMOFs) was designed, characterized and applied for the preparation of pyridines fused with pyrazole and pyrimidine under ultrasonic irradiation.

**Figure 2:**
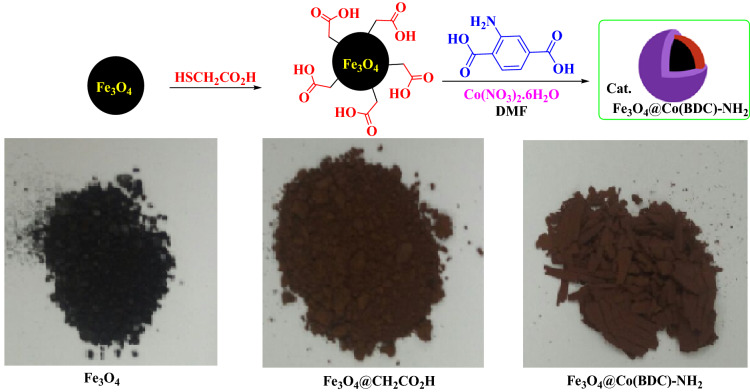
Stepwise synthesis of the nanomagnetic metal–organic frameworks (NMMOFs) system.

At first, nanomagnetic metal–organic frameworks (NMMOFs) were synthesized (Scheme 2). Its schematic synthesis is showed in Fig. 2. The synthesized Fe_3_O_4_@Co(BDC)-NH_2_ fully characterized by applying FT-IR, XRD, SEM, elemental mapping, energy dispersive X-ray (EDS), TG, DTG, VSM and N_2_ adsorption–desorption isotherms (BET).

FT-IR spectrum of H_2_BDC-NH_2_, Fe_3_O_4_, Fe_3_O_4_@CH_2_CO_2_H, and Fe_3_O_4_@Co(BDC)-NH_2_ are shown in Figure S1 (see supporting information). The absorption bands at 670 cm^−1^ linked to the stretching vibrational modes of Fe–O groups in Fe_3_O_4_. The absorption bands at 1741, 2924, and 3426 cm^−1^ related to C=O, C–H and, O–H stretching respectively in Fe_3_O_4_@CH_2_CO_2_H. Also, the absorption bands at 633 cm^−1^ and 3318–3448 cm^−1^ are related to Co–O and N–H_2_ stretching respectively, of Fe_3_O_4_@Co(BDC)-NH_2_. Finally, the differences between H_2_BC-NH_2_, Fe_3_O_4_, Fe_3_O_4_@CH_2_CO_2_H, and Fe_3_O_4_@Co(BDC)-NH_2_ in the FT-IR spectrum were confirmed the synthesis of Fe_3_O_4_@Co(BDC)-NH_2_.

**Figure 3:**
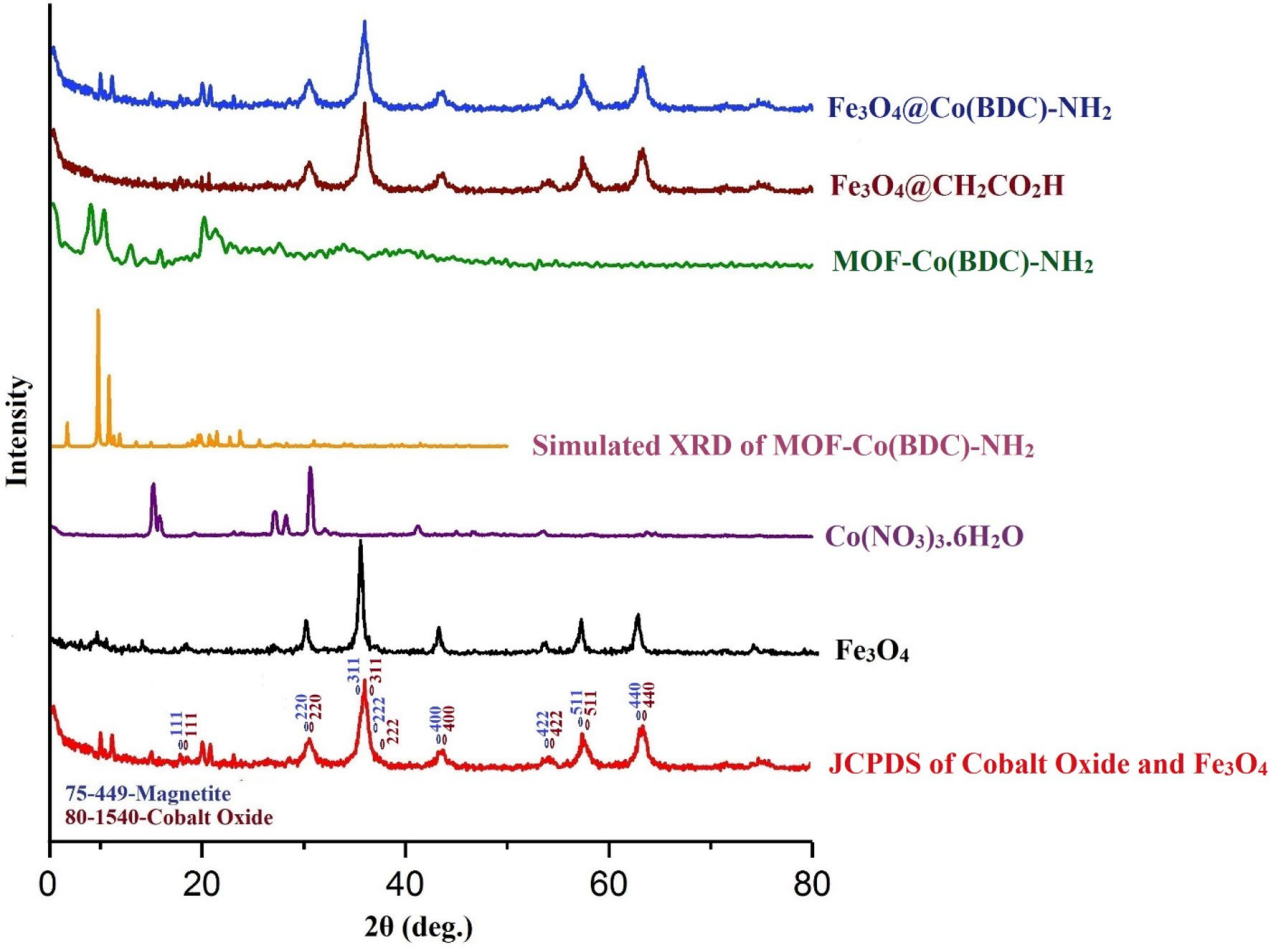
Comparison XRD pattern of JCPDS (red line), Fe_3_O_4_ (black line), Co(NO_3_)_3_·6H_2_O (purple line), Simulated XRD (orange line), MOF-Co(BDC)-NH_2_ (green line), Fe_3_O_4_@CH_2_CO_2_H (brown line) and Fe_3_O_4_@Co(BDC)-NH_2_ (blue line).

**Figure 4:**
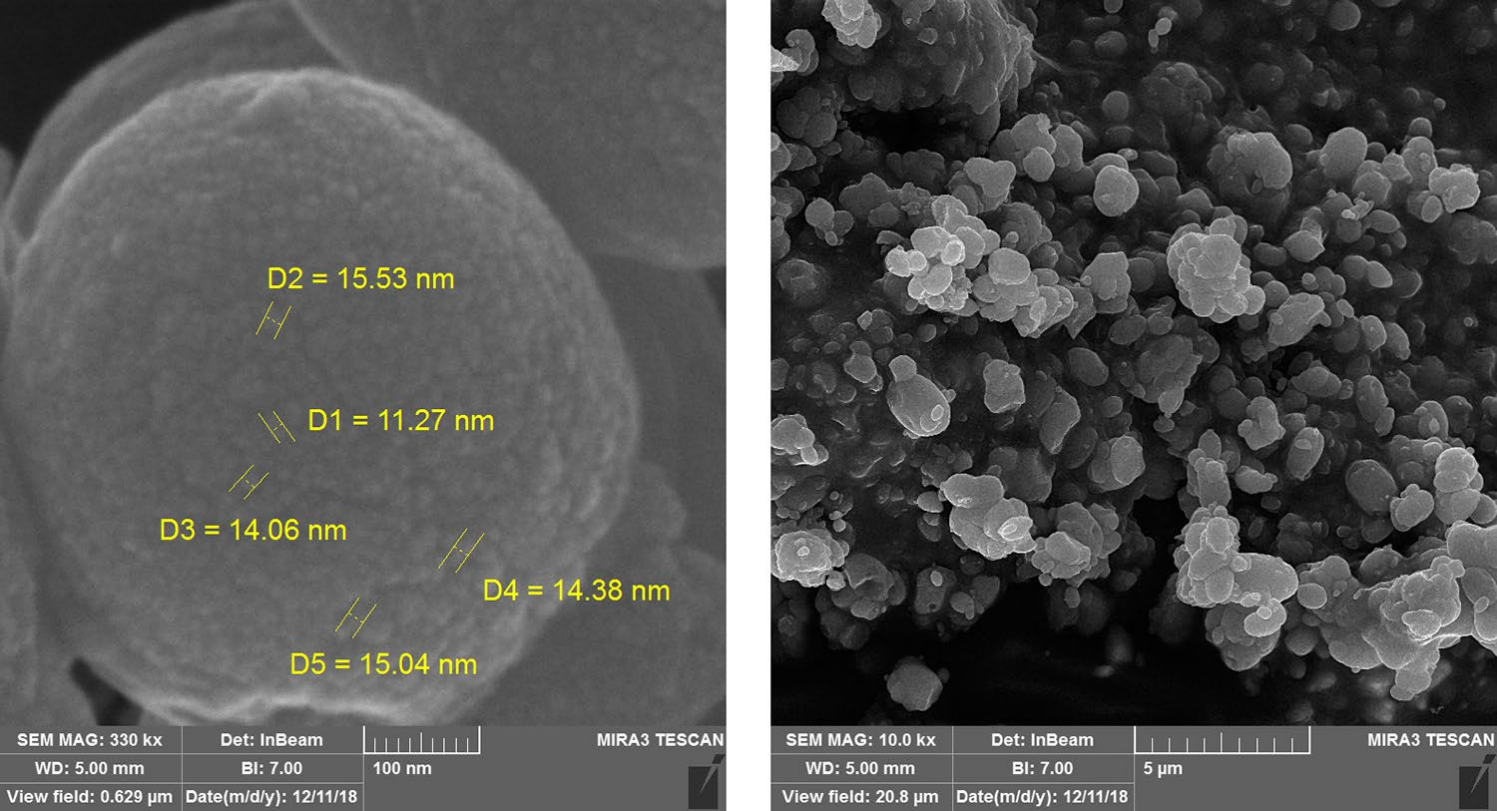
Scanning electron microscope (SEM) images of Fe_3_O_4_@Co(BDC)-NH_2_.

The particle size and shape, as well as the morphology of Fe_3_O_4_@CH_2_CO_2_H, Fe_3_O_4_, MOF-Co(BDC)-NH_2_, Co(NO_3_)_3_·6H_2_O and Fe_3_O_4_@Co(BDC)-NH_2_ were studied by XRD (Fig. 3), and SEM (Fig. 4). The comparison XRD pattern of JCPDS (red line), Fe_3_O_4_ (black line), Co(NO_3_)_3_·6H_2_O (purple line), Simulated XRD (orange line), MOF-Co(BDC)-NH_2_ (green line), Fe_3_O_4_@CH_2_CO_2_H (brown line) and Fe_3_O_4_@Co(BDC)-NH_2_ (blue line) is assembled according to the liturture servey at the range of 5°–80° in Fig. 3^42^. The phase of Co oxide and Fe_3_O_4_ in Fe_3_O_4_@Co(BDC)-NH_2_ as standard brown line (ICDD Card: 80-1540) of Co and pinks standard line (JCP2: 75-449) of Fe_3_O_4_ in the standard references. Also, Peaks of Fe_3_O_4_@Co(BDC)-NH_2_ exhibited 2θ = 18.3°, 30.2°, 35.6°, 43.3°, 53.6°, 57.3°, 62.8° and 74.2° corresponding to diffraction lines (111), (220), (311), (400), (422), (511), (440) and (533). Then, the averaged interlunar distance and sizes of crystal were calculated by the Scherer equation and Bragg equation, which are determined 0.67 nm (single peak at 12.8 and 17.5–37.5 nm range (Table S1 see in supporting information)^43,44^.

For comparison, structure and elementals in the synthesis of step by step Fe_3_O_4_@CH_2_CO_2_H and Fe_3_O_4_@Co(BDC)-NH_2_ were also studied with energy dispersive X-ray analysis (EDX) analysis (Figure S2 see supporting information). The structures of Fe_3_O_4_@Co(BDC)-NH_2_ and Fe_3_O_4_@CH_2_CO_2_H were verified with existence of Fe, Co, N, C, O and Fe, C, O, and S atoms respectively ^45^. Then, elementals dispersed over the surface of the catalyst, and step Fe_3_O_4_@Co(BDC)-NH_2_ was checked out by SEM-elemental mapping (Figure S3 see supporting information). The images in Figure S3 shows that all kinds of elements are well dispersed over the surface of Fe_3_O_4_@Co(BDC)-NH_2_. The difference between EDX analysis and SEM-elemental mapping is confirmed by the structure of Fe_3_O_4_@Co(BDC)-NH_2_.

**Figure 5:**
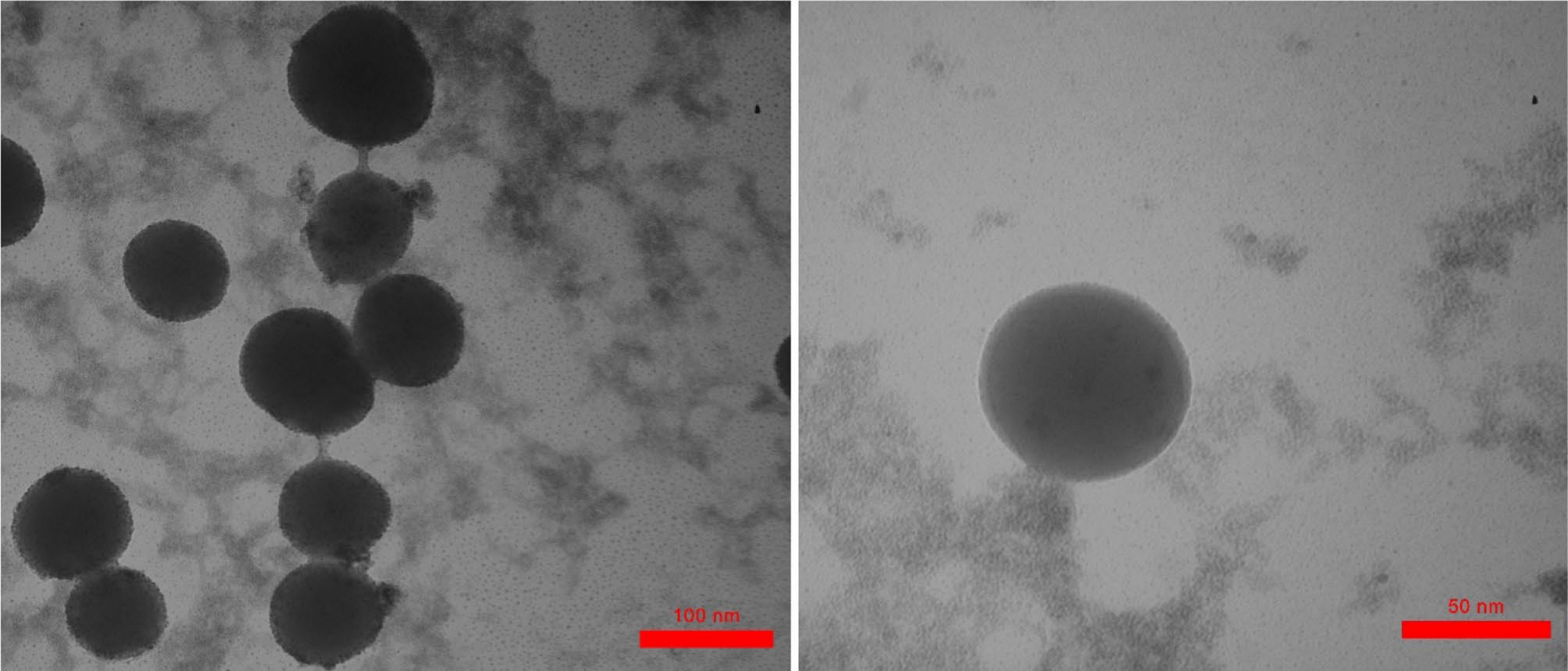
Transmission electron microscopy (TEM) images of Fe_3_O_4_@Co(BDC)-NH_2_.

In another investigation, the particle size and shape, as well as the morphology of Fe_3_O_4_@Co(BDC)-NH_2_ were examined by scanning electron microscope (SEM) (Fig. 4). As shown in Fig. 4, nano-spherical particles of the nanomagnetic metal–organic frameworks (NMMOFs) are in the nanoscale, as the particles are quite overlapped with different crystallite size as observed in SEM Transmission electron microcopy (TEM) images of Fe_3_O_4_@Co(BDC)-NH_2_ catalyst reveal that the particles shape is spherical and the particle size is up to 50 nm (Fig. 5).

**Figure 6:**
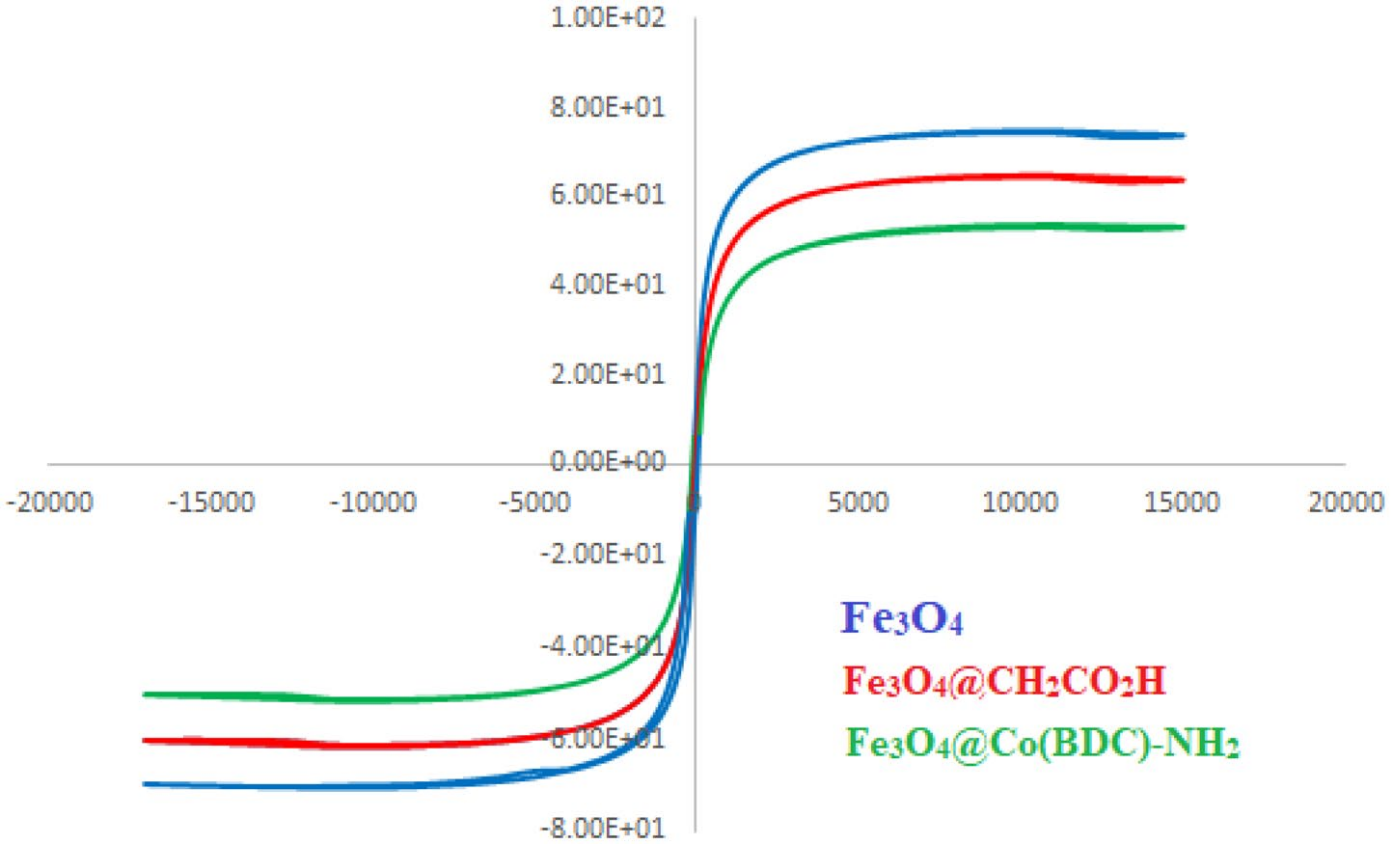
Vibrating sample magnetometer (VSM) of Fe_3_O_4,_ Fe_3_O_4_@CH_2_CO_2_H, and Fe_3_O_4_@Co(BDC)-NH_2_.

The magnetic measurement of Fe_3_O_4,_ Fe_3_O_4_@CH_2_CO_2_H, and Fe_3_O_4_@Co(BDC)-NH_2_ are shown in Fig. 6. Based on Fig. 6, the vibrating sample magnetometer (VSM) of Fe_3_O_4_, Fe_3_O_4_@CH_2_CO_2_H, and Fe_3_O_4_@Co(BDC)-NH_2_ were examined and reduced from 64.4, 60.1 up to 54.3 μg^−1^ respectively. Therefore, these decreases are the result of coating with its corresponding layers.

In another investigation, the structural and thermal stability of Fe_3_O_4_@Co(BDC)-NH_2_ was also determined using the technique of the thermal gravimetric (TG), derivative thermal gravimetric (DTG), as well as the differential thermal analysis (DTA) (Figure S4 see in supporting information). First stage weight loss is about 100 °C, associated with the removal of possible solvents (organic and water), which was used in the course of catalyst preparation. Then, twice a step of weight loss has occurred at about 300 °C, which is the onset of the structural degradation of the catalyst.

**Figure 7:**
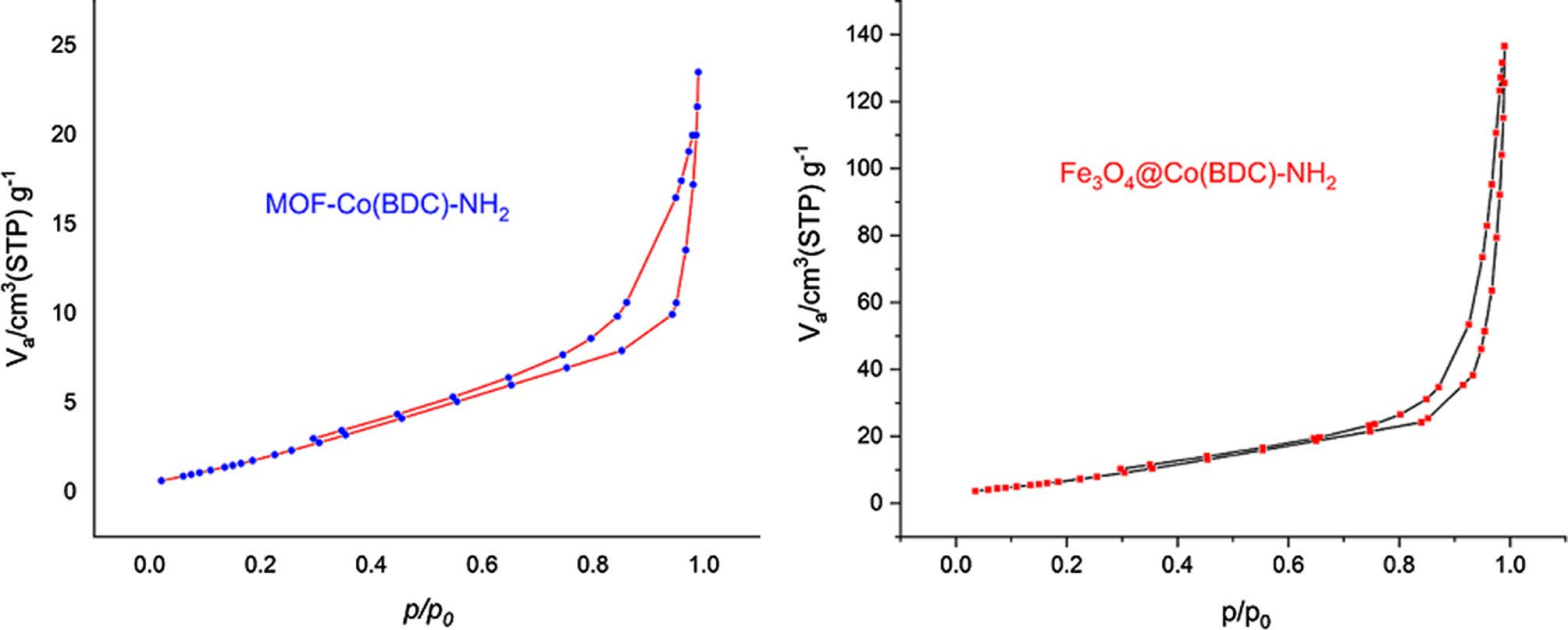
Nitrogen adsorption–desorption isotherm (BET) of Fe_3_O_4_@Co(BDC)-NH_2_ and MOF-Co(BDC)-NH_2_.

For the determination of surface structural parameters, the *N*_*2*_ adsorption/desorption technique was used. The results of *N*_*2*_ adsorption/desorption were plotted in Fig. 7. The obtained surface area based on BET isotherm is 22.35 m^2^ g^−1^. The total pore volume of the catalyst is 0.02 cm^3^ g^−1^. Also, for studying the textural properties of MOF-Co(BDC)-NH_2_ the N_2_ adsorption–desorption isotherms were used (Fig. 7). The adsorption isotherm is type III and the appearance of hysteresis loop shows the presence of mesopores in the sample. The calculated surface areas based on BET equation and total pore volumes are 86 m^2^ g^−1^ and 0.36 cm^3^ g^−1^ respectively. The pore size distribution of MOF-Co(BDC)-NH_2_ based on BJH method is shown (Figure S5 see in supporting information). This plot clearly shows presence of micropores (size < 2 nm) and mesopores (2 < size < 50 nm) in the sample, however the micropores are more abundant.

After the synthesis and characterization of Fe_3_O_4_@Co(BDC)-NH_2_, it was applied for the synthesis of novel mono, bis and tris novel fused pyridines and 1,4-dihydropyridines with pyrazole and pyrimidine moieties by using the corresponding precursors such as 3-methyl-1,4-diphenyl-1,8-dihydro-5*H*-pyrazolo[4′,3′:5,6]pyrido[2,3-*d*]pyrimidine-5,7(6*H*)-dione and 3-methyl-4-phenyl-1,4,8,9-tetrahydro-5*H*-pyrazolo[4′,3′:5,6]pyrido[2,3-*d*]pyrimidine-5,7(6*H*)-dione. The above mentioned products were obtained by reaction of 4-nitro benzaldehyde (1.0 mmol, 0.151 g), 3-methyl-1-phenyl-1*H*-pyrazole-5-amine (1.0 mmol, 0.174 g) and pyrimidine-2,4,6(1*H*,3*H*,5*H*)-trione (1.0 mmol, 0.128 g) as a model for the optimization the reaction conditions. The optimized data is listed in Table 1. As shown in Table 1, the best of choice for the synthesis of 3-methyl-1,4-diphenyl-1,8-dihydro-5*H*-pyrazolo[4′,3′:5,6]pyrido[2,3-*d*]pyrimidine-5,7(6*H*)-dione was achieved in the presence of 10 mg Fe_3_O_4_@Co(BDC)-NH_2_ in DMF (5 mL) as solvent under ultrasonic irradiation (entry 4, Table 1). The model reaction was also studied by using several solvents such as H_2_O, CH_3_CN, *n*-hexane, CHCl_3_, MeOH, EtOH, CH_2_Cl_2_, EtOAc (5 mL) and solvent-free condition in the presence of 10 mg of Fe_3_O_4_@Co(BDC)-NH_2_. The results of the reaction did not improve (Table 1, entries 6–13). Also, the model reaction was also studied in the magnetic stirrer condition at room temperature under the solvent-free reaction (Table 1, entry 14).

**Table 1:** Effect of different amounts of catalysts and solvent (5 mL) in the synthesis of novel fused pyridines and 1,4-dihydropyridines with pyrazole and pyrimidine moieties by using the corresponding precursors under ultrasonic irradiation.


Entry	Solvent	Cat. (mg)	Sonication (min)	Yield (%)
1	DMF	–	120	Trace
2	DMF	5	75	25
3	DMF	7.5	60	45
4	DMF	10	50	72
5	DMF	15	50	72
6	H_2_O	10	60	35
7	*n*-Hexane	10	60	25
8	EtOH	10	60	Trace
9	CH_2_Cl_2_	10	60	Trace
10	CHCl_3_	10	60	28
11	EtOAc	10	60	46
12	CH_3_CN	10	60	54
13	MeOH	10	60	Trace
14	–	10	60	15

Reaction conditions: 3-methyl-1-phenyl-1*H*-pyrazole-5-amine (1.0 mmol, 0.174 g), pyrimidine-2,4,6(1*H*,3*H*,5*H*)-trione (1.0 mmol, 0.128 g) and 4-nitrobenzaldehyde (1.0 mmol, 0.151 g).

After optimizing the reaction conditions, Fe_3_O_4_@Co(BDC)-NH_2_(10 mg) is applied to synthesis a good range of novel biological and pharmacological candidate compounds using various aromatic aldehydes (trephetaldehyde, iso-trephetaldehyde, tris-aldehyde, bearing electron-donating and electron-withdrawing groups), pyrimidine (1,3-dimethylpyrimidine-2,4,6(1*H*,3*H*,5*H*)-trione, pyrimidine-2,4,6(1*H*,3*H*,5*H*)-trione) and pyrazole-5-amine (3-methyl-1*H*-pyrazole-5-amine, 3-methyl-1-phenyl-1*H*-pyrazole-5-amine) derivatives. As shown in Table 2, the obtained results indicated that Fe_3_O_4_@Co(BDC)-NH_2_ is appropriate for the preparation of target molecules in high to excellent yields (65–90%) with in relatively short reaction times (20–40 min). Furthermore, the model reaction is tested by the reaction of 3-methyl-1*H*-pyrazole-5-amine, and 3-methyl-1*H*-pyrazole-5-amine to give a mixture of the corresponding pyridine and 1,4-dihydropyridine respectively.

**Table 2:**
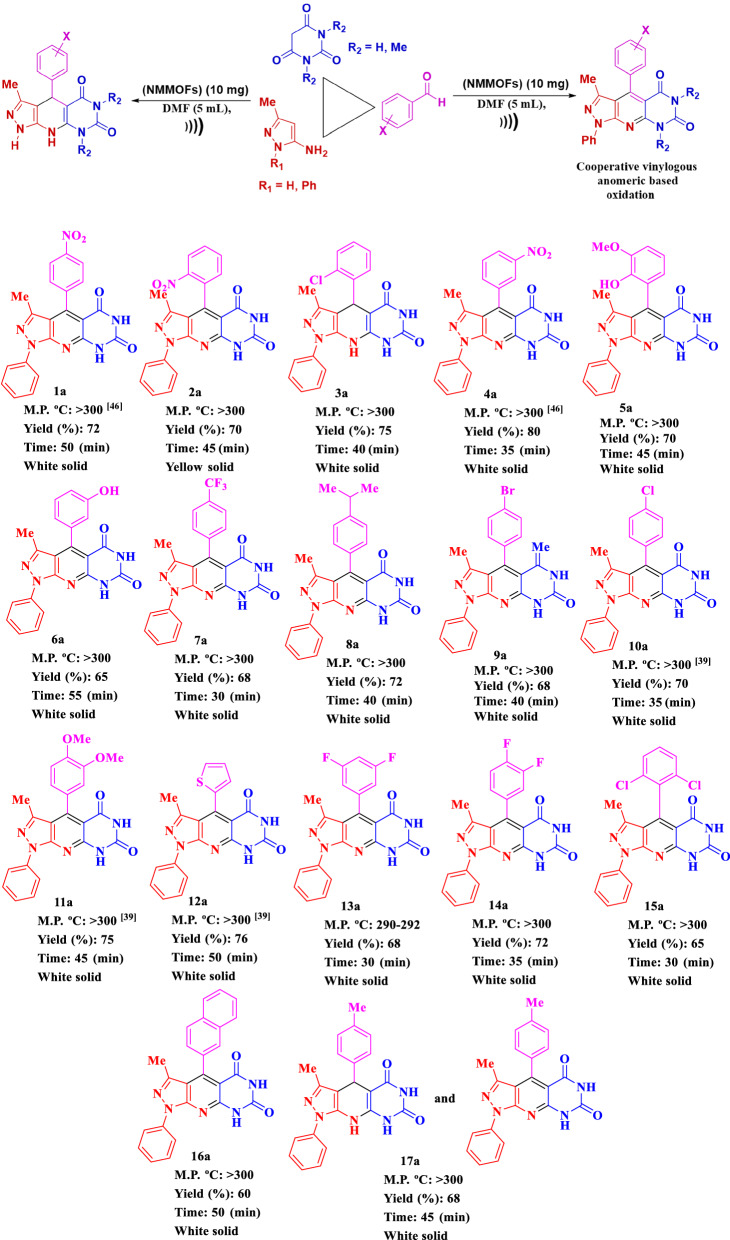

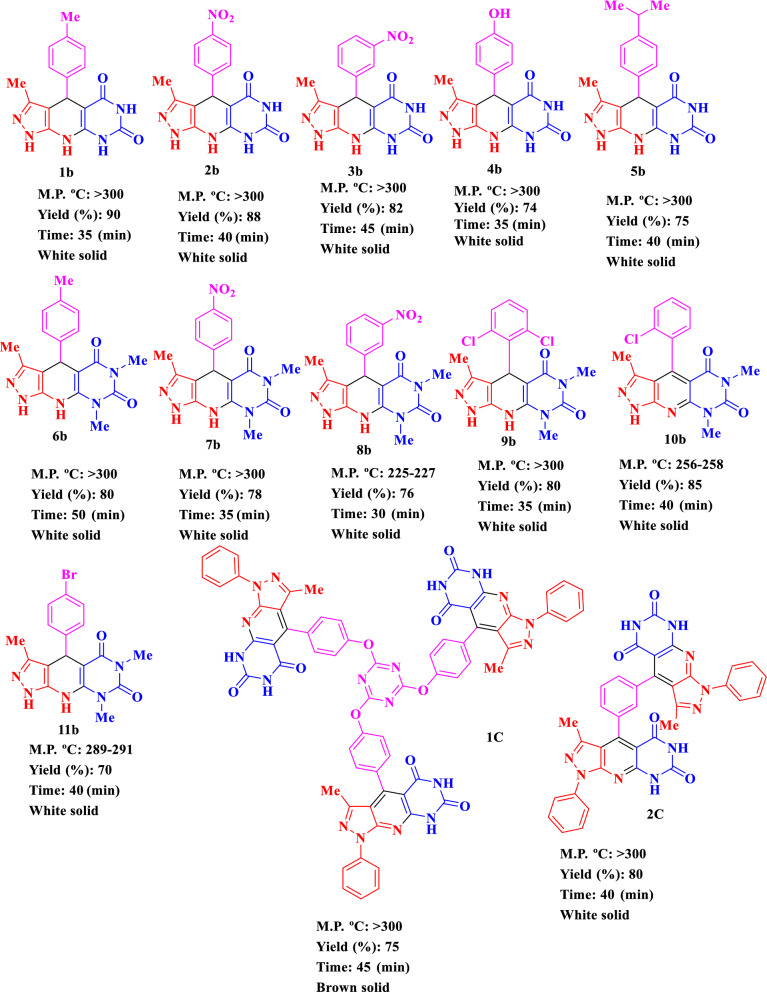
Synthesis of (**a**) 3-methyl-1,4-diphenyl-1,8-dihydro-5*H*-pyrazolo[4′,3′:5,6]pyrido[2,3-*d*]pyrimidine-5,7(6*H*)-dione derivatives, (**b**) 3-Methyl-4-phenyl-1,4,8,9-tetrahydro-5*H*-pyrazolo[4′,3′:5,6]pyrido[2,3-*d*]pyrimidine-5,7(6*H*)-dione derivatives, (**c**) Bis and tris 3-methyl-1,4-diphenyl-1,8-dihydro-5*H*-pyrazolo[4′,3′:5,6]pyrido[2,3-*d*]pyrimidine-5,7(6*H*)-dione derivatives using Fe_3_O_4_@Co(BDC)-NH_2_ under ultrasonic irradiation.

**Scheme 3:**
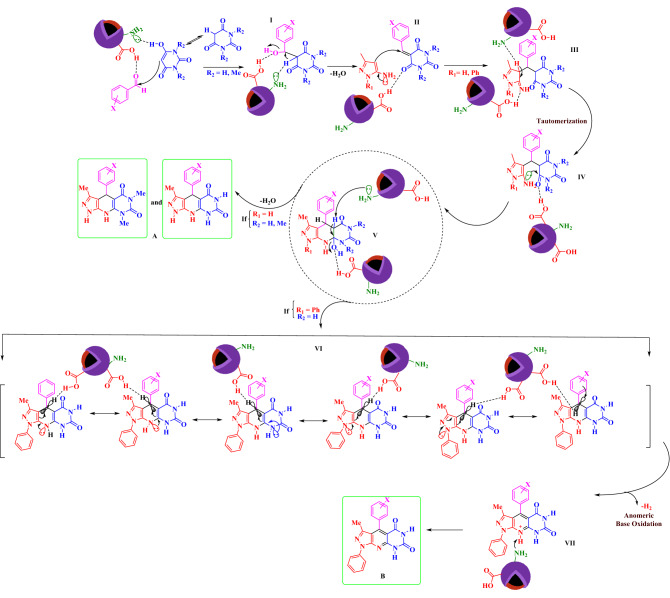
Proposed mechanism for the preparation of novel fused pyridines and 1,4-dihydropyridines with pyrazole and pyrimidine moieties via a cooperative vinylogous anomeric based oxidation.

In the proposed mechanism, the aldehyde is activated by Fe_3_O_4_@Co(BDC)-NH_2_. In the initial step, intermediate (**I**) is produced by the reaction of pyrimidine (R_2_ = H, Me) and activated aldehyde. In the next step, intermediate (**II**) is prepared with losing one molecule of H_2_O. In the third step, pyrazole-5-amine (R_1_ = H, Ph) derivatives react with intermediate (**II**) to gives intermediate (**IV**) after tautomerization. Then, intermediate (**IV**) gives intermediate (**V**) after intramolecular cyclization and losing another molecule of H_2_O. In the last step, the lone pair electrons of N atoms of 1,4-dihydropyridine (**VI**) interacts through C–C double bonds with a vacant anti-bonding orbital of C–H bond (n_N_→ σ_C–H_^*^ and π_C=C_→ σ_C–H_^*^) and weaken it, so that is favoring for hydride transfer and H_2_ releasing from intermediate **VI** to generate its corresponding pyridinium salt. The achieved data from the optimization of described reaction under argon and nitrogen atmospheres verified our suggestion for oxidation and aromatization of intermediate **VI**. On the other hand, 1,4-dihydropyridine (**VI**) is converted to its corresponding pyridinium intermediate (**VII**), via a cooperative vinylogous anomeric based oxidation and releasing a hydrogen molecule (–H_2_)^47–55^. Finally, the desired pyridine fused with pyrazole and pyrimidine moiety (**B**) is obtained via removing a proton from the pyridinium intermediate (**VII**). When, 3-methyl-1*H*-pyrazole-5-amine was used instead of 3-methyl-1-phenyl-1*H*-pyrazole-5-amine after intramolecular cyclization and losing a molecule of water, intermediate (**VI**) is converted to product (**A**) (Scheme 3). Interestingly, the 1,4-dihydropyridines (**A**) did not convert to their corresponding pyridines.

**Figure 8:**
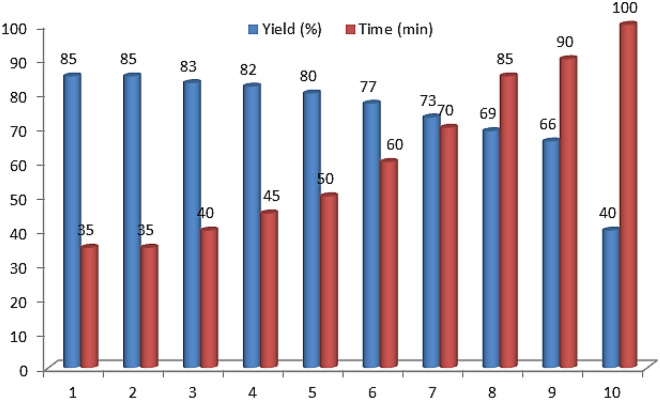
Recyclability of Fe_3_O_4_@Co(BDC)-NH_2_ as a catalyst in the synthesis pyrazolo[4′,3′:5,6]pyrido[2,3-*d*]pyrimidine-5,7(6*H*)-dione.

The reusability of Fe_3_O_4_@Co(BDC)-NH_2_ was also investigated. The reaction of 4-nitro benzaldehyde (1.0 mmol, 0.151 g), 3-methyl-1-phenyl-1*H*-pyrazole-5-amine (1.0 mmol, 0.174 g) and pyrimidine-2,4,6(1*H*,3*H*,5*H*)-trione (1.0 mmol, 0.128 g) was selected as a model reaction under ultrasonic irradiation. The nanomagnetic metal–organic frameworks (NMMOFs) catalyst was separated by an external magnet, washed with DMF and dried. The results indicated that the catalyst could be utilized for nine runs without any significant loss of its initial catalytic activity, which can be ascribed to the high stability of the synthesized catalyst (Fig. 8). Then, the reused catalyst was also characterized by FT-IR spectrum (Figure S6 see supporting information**)**, *N*_*2*_ adsorption–desorption isotherm (BET) and scanning electron microscope (SEM) images. The obtained spectra are as same as the corresponding spectra of fresh catalyst (Figures S7, S8 see supporting information), Furthermore, to compare the performance of nanomagnetic metal–organic frameworks (NMMOFs) catalyst for the synthesis of desired fused pyridines and 1,4-dihydropyridines with pyrazole and pyrimidine moieties via a cooperative vinylogous anomeric based oxidation, we have used various organic and inorganic acid catalysts for condensation reaction between 4-nitro benzaldehyde (1.0 mmol, 0.151 g), 3-methyl-1-phenyl-1*H*-pyrazole-5-amine (1.0 mmol, 0.174 g) and pyrimidine-2,4,6(1*H*,3*H*,5*H*)-trione (1.0 mmol, 0.128 g). As Table 3 indicates, Fe_3_O_4_@Co(BDC)-NH_2_ is the best of choice for the synthesis of pyrazolo[4′,3′:5,6]pyrido[2,3-*d*]pyrimidine-5,7(6*H*)-dione (Table 3).

**Table 3:** Synthesis of pyrazolo[4′,3′:5,6]pyrido[2,3-d]pyrimidine-5,7(6*H*)-dionein the presence of various catalysts under ultrasonic irradiation.

Entry	Catalyst	(mol%)	Time (min)	Yield (%)
1	FeCl_3_	10	120	25
2	[PVI-SO_3_H]FeCl^56^	10	120	48
3	Fe_3_O_4_	10 mg	120	Trace
4	NH_4_NO_3_	10	120	Trace
5	SSA^57,58^	10 mg	120	35
6	Nano-SB-[PSIM]Cl^59^	10 mg	120	Trace
7	NaHSO_4_	10	120	–
8	GTBSA^60^	10	120	55
9	SBA-15/(CH_2_)_3_ N(CH_2_PO_3_H_2_)_2_^61^	10 mg	120	35
10	[Py-SO_3_H]Cl^62^	10	120	35
11	*p*-TSA	10	120	25
12	Et_3_N	10	120	–
13	MIL-100(Cr)/NHEtN(CH_2_PO_3_H_2_)_2_^63^	10 mg	120	63
14	APVPB^64^	10 mg	120	40
15	MHMHPA^65^	10	120	55
16	GTMPA^66^	10	120	40
17	Co(NO_3_)_3_·6H_2_O	10	120	36
18	H_2_BDC-NH_2_	10	120	42
19	Fe_3_O_4_@Co(BDC)-NH_2_	10 mg	50	72^a^

^a^This work.

## Conclusion

In summary, a novel core–shell nanomagnetic metal–organic frameworks Fe_3_O_4_@Co(BDC)-NH_2_ as a new catalyst was prepared and fully characterized. This catalyst was applied for the synthesis of a range of novel fused pyridines and 1,4-dihydropyridines with pyrazole and pyrimidine moieties with good yields via a cooperative vinylogous anomeric based oxidation mechanism under ultrasonic irradiation. The obtained biological-based compounds are suitable candidates for biological studies. The described catalyst is reusable and easily separated by an external magnet.

## Supplementary Information

Supplementary Figures.
