# Supplementary material for: Application of novel nanomagnetic metal–organic frameworks as a catalyst for the synthesis of new pyridines and 1,4-dihydropyridines via a cooperative vinylogous anomeric based oxidation
Source: Sci Rep. 2021 Mar 5;11:5279. doi: 10.1038/s41598-021-84005-2 (PMC7935861; doi:10.1038/s41598-021-84005-2)
Supplement: Supplementary file 1 — Supplementary Figures. [file 41598_2021_84005_MOESM1_ESM.docx]

**Application of novel nanomagnetic metal-organic frameworks as a catalyst for the synthesis of new pyridines and 1,4-dihydropyridines *via* a cooperative vinylogous anomeric based oxidation**

Hassan Sepehrmansourie,^a^ Mahmoud Zarei,*^a^ Mohammad Ali Zolfigol,*^a^ Saeed Babaee ^a^ and Sadegh Rostamnia*^b^

^a^ Department of Organic Chemistry, Faculty of Chemistry, Bu-Ali Sina University, Hamedan 6517838683, Tel: +988138282807, Fax: +988138380709 Iran. E-Mail: mahmoud8103@yahoo.com, [zolfi@basu.ac.ir &](mailto:zolfi@basu.ac.ir%20&) [mzolfigol@yahoo.com](mailto:mzolfigol@yahoo.com) (M. A. Zolfigol).

^b^ Organic and Nano Group (ONG), Department of Chemistry, Faculty of Science, University of Maragheh, PO Box 55181-83111, Maragheh, Iran. E-Mail: rostamnia@maragheh.ac.ir

^c^ Organic and Nano Group (ONG), Department of Chemistry, Faculty of Science, University of Maragheh, PO Box 55181-83111, Maragheh, Iran. E-Mail: rostamnia@maragheh.ac.ir

**Figure S1:** FT-IR spectrum of H_2_BDC-NH_2_, Fe_3_O_4_, Fe_3_O_4_@CH_2_CO_2_H, and Fe_3_O_4_@Co(BDC)-NH_2_. ...........................................................................................................................................................8

**Figure S2:** Energy dispersive X-ray analysis (EDX) of Fe_3_O_4_@CH_2_CO_2_H and Fe_3_O_4_@Co(BDC)-NH_2_.....................................................................................................................................................9

**Figure S3:** Elemental mapping of C (red); O (blue), N (green); Fe (violet) and Co (yellow-orange) atoms for Fe_3_O_4_@Co(BDC)-NH_2_..........................................................................................................................10

**Figure S4:** Thermal gravimetric (TG) and differential thermal gravimetric (DTG) profile of Fe_3_O_4_@Co(BDC)-NH_2_...................................................................................................................................................11

**Figure S5:** BJH of Fe_3_O_4_@Co(BDC)-NH_2_ and MOF-Co(BDC)-NH_2_.............................................................12

**Figure S7:** Nitrogen adsorption-desorption isotherm (BET) of recycled Fe_3_O_4_@Co(BDC)-NH_2_..........................12

**Figure S8:** Scanning electron microscope (SEM) images of recycled Fe_3_O_4_@Co(BDC)-NH_2_..............................13

**Table S1:** XRD data of Fe_3_O_4_@Co(BDC)-NH_2_..........................................................................................13

Spectral data .....................................................................................................................................14

[FT-IR Spectrum of 3-methyl-4-(4-nitrophenyl)-1-phenyl-1,8-dihydro-5*H*-pyrazolo[4',3':5,6]pyrido[2,3-*d*]pyrimidine-5,7(6*H*)-dione (1a)](#_Toc38178898) ..23

[^1^H-NMR Spectrum of 3-methyl-4-(4-nitrophenyl)-1-phenyl-1,8-dihydro-5*H*-pyrazolo[4',3':5,6]pyrido[2,3-*d*]pyrimidine-5,7(6*H*)-dione (1a) 24](#_Toc38178899)

[^13^C-NMR Spectrum of 3-methyl-4-(4-nitrophenyl)-1-phenyl-1,8-dihydro-5*H*-pyrazolo[4',3':5,6]pyrido[2,3-*d*]pyrimidine-5,7(6*H*)-dione (1a)](#_Toc38178900) 25

[FT-IR Spectrum of 3-methyl-4-(2-nitrophenyl)-1-phenyl-1,8-dihydro-5*H*-pyrazolo[4',3':5,6]pyrido[2,3-*d*]pyrimidine-5,7(6*H*)-dione (2a)](#_Toc38178901) 26

[^1^H-NMR Spectrum of 3-methyl-4-(2-nitrophenyl)-1-phenyl-1,8-dihydro-5*H*-pyrazolo[4',3':5,6]pyrido[2,3-*d*]pyrimidine-5,7(6*H*)-dione (2a)](#_Toc38178902) 27

[^13^C-NMR Spectrum of 3-methyl-4-(2-nitrophenyl)-1-phenyl-1,8-dihydro-5*H*-pyrazolo[4',3':5,6]pyrido[2,3-*d*]pyrimidine-5,7(6*H*)-dione (2a)](#_Toc38178903) 28

[FT-IR Spectrum of 4-(2-chlorophenyl)-3-methyl-1-phenyl-1,4,8,9-tetrahydro-5*H*-pyrazolo[4',3':5,6]pyrido[2,3-*d*]pyrimidine-5,7(6*H*)-dione (3a)](#_Toc38178904) 29

[^1^H-NMR Spectrum of 4-(2-chlorophenyl)-3-methyl-1-phenyl-1,4,8,9-tetrahydro-5*H*-pyrazolo[4',3':5,6]pyrido[2,3-*d*]pyrimidine-5,7(6*H*)-dione (3a)](#_Toc38178905) 30

[^13^C-NMR Spectrum of 4-(2-chlorophenyl)-3-methyl-1-phenyl-1,4,8,9-tetrahydro-5*H*-pyrazolo[4',3':5,6]pyrido[2,3-*d*]pyrimidine-5,7(6*H*)-dione (3a)](#_Toc38178906) 31

[FT-IR Spectrum of 3-methyl-4-(3-nitrophenyl)-1-phenyl-1,8-dihydro-5*H*-pyrazolo[4',3':5,6]pyrido[2,3-*d*]pyrimidine-5,7(6*H*)-dione (4a)](#_Toc38178907) 32

[^1^H-NMR Spectrum of 3-methyl-4-(3-nitrophenyl)-1-phenyl-1,8-dihydro-5*H*-pyrazolo[4',3':5,6]pyrido[2,3-*d*]pyrimidine-5,7(6*H*)-dione (4a)](#_Toc38178908) 33

[^13^C-NMR Spectrum of 3-methyl-4-(3-nitrophenyl)-1-phenyl-1,8-dihydro-5*H*-pyrazolo[4',3':5,6]pyrido[2,3-*d*]pyrimidine-5,7(6*H*)-dione (4a)](#_Toc38178909) 34

[FT-IR Spectrum of 4-(2-hydroxy-3-methoxyphenyl)-3-methyl-1-phenyl-1,8-dihydro-5*H*-pyrazolo[4',3':5,6]pyrido[2,3-*d*]pyrimidine-5,7(6*H*)-dione (5a)](#_Toc38178910) 35

[^1^H-NMR Spectrum of 4-(2-hydroxy-3-methoxyphenyl)-3-methyl-1-phenyl-1,8-dihydro-5*H*-pyrazolo[4',3':5,6]pyrido[2,3-*d*]pyrimidine-5,7(6*H*)-dione (5a)](#_Toc38178911) 36

[^13^C-NMR Spectrum of 4-(2-hydroxy-3-methoxyphenyl)-3-methyl-1-phenyl-1,8-dihydro-5*H*-pyrazolo[4',3':5,6]pyrido[2,3-*d*]pyrimidine-5,7(6*H*)-dione (5a)](#_Toc38178912) 37

[FT-IR Spectrum of 4-(3-hydroxyphenyl)-3-methyl-1-phenyl-1,8-dihydro-5*H*-pyrazolo[4',3':5,6]pyrido[2,3-*d*]pyrimidine-5,7(6*H*)-dione (6a)](#_Toc38178913) 38

[^1^H-NMR Spectrum of 4-(3-hydroxyphenyl)-3-methyl-1-phenyl-1,8-dihydro-5*H*-pyrazolo[4',3':5,6]pyrido[2,3-*d*]pyrimidine-5,7(6*H*)-dione (6a)](#_Toc38178914) 39

[^13^C-NMR Spectrum of4-(3-hydroxyphenyl)-3-methyl-1-phenyl-1,8-dihydro-5*H*-pyrazolo[4',3':5,6]pyrido[2,3-*d*]pyrimidine-5,7(6*H*)-dione (6a)](#_Toc38178915) 40

[FT-IR Spectrum of 3-methyl-1-phenyl-4-(4-(trifluoromethyl)phenyl)-1,8-dihydro-5*H*-pyrazolo[4',3':5,6]pyrido[2,3-*d*]pyrimidine-5,7(6*H*)-dione (7a)](#_Toc38178916) 41

[^1^H-NMR Spectrum of 3-methyl-1-phenyl-4-(4-(trifluoromethyl)phenyl)-1,8-dihydro-5*H*-pyrazolo[4',3':5,6]pyrido[2,3-*d*]pyrimidine-5,7(6*H*)-dione (7a)](#_Toc38178917) 42

[^13^C-NMR Spectrum of 3-methyl-1-phenyl-4-(4-(trifluoromethyl)phenyl)-1,8-dihydro-5*H*-pyrazolo[4',3':5,6]pyrido[2,3-*d*]pyrimidine-5,7(6*H*)-dione (7a)](#_Toc38178918) 43

[FT-IR Spectrum of 4-(4-isopropylphenyl)-3-methyl-1-phenyl-1,8-dihydro-5*H*-pyrazolo[4',3':5,6]pyrido[2,3-*d*]pyrimidine-5,7(6*H*)-dione (8a)](#_Toc38178919) 44

[^1^H-NMR Spectrum of 4-(4-isopropylphenyl)-3-methyl-1-phenyl-1,8-dihydro-5*H*-pyrazolo[4',3':5,6]pyrido[2,3-*d*]pyrimidine-5,7(6*H*)-dione (8a)](#_Toc38178920) 45

[^13^C-NMR Spectrum of 4-(4-isopropylphenyl)-3-methyl-1-phenyl-1,8-dihydro-5*H*-pyrazolo[4',3':5,6]pyrido[2,3-*d*]pyrimidine-5,7(6*H*)-dione (8a)](#_Toc38178921) 46

[FT-IR Spectrum of 4-(4-bromophenyl)-3-methyl-1-phenyl-1,8-dihydro-5*H*-pyrazolo[4',3':5,6]pyrido[2,3-*d*]pyrimidine-5,7(6*H*)-dione (9a)](#_Toc38178922) 47

[^1^H-NMR Spectrum of 4-(4-bromophenyl)-3-methyl-1-phenyl-1,8-dihydro-5H-pyrazolo[4',3':5,6]pyrido[2,3-*d*]pyrimidine-5,7(6*H*)-dione (9a)](#_Toc38178923) 48

[^13^C-NMR Spectrum of 4-(4-bromophenyl)-3-methyl-1-phenyl-1,8-dihydro-5*H*-pyrazolo[4',3':5,6]pyrido[2,3-*d*]pyrimidine-5,7(6*H*)-dione (9a)](#_Toc38178924) 49

[FT-IR Spectrum of 4-(4-chlorophenyl)-3-methyl-1-phenyl-1,8-dihydro-5*H*-pyrazolo[4',3':5,6]pyrido[2,3-*d*]pyrimidine-5,7(6*H*)-dione (10a)](#_Toc38178925) 50

[^1^H-NMR Spectrum of 4-(4-chlorophenyl)-3-methyl-1-phenyl-1,8-dihydro-5*H*-pyrazolo[4',3':5,6]pyrido[2,3-*d*]pyrimidine-5,7(6*H*)-dione (10a)](#_Toc38178926) 51

[^13^C-NMR Spectrum of 4-(4-chlorophenyl)-3-methyl-1-phenyl-1,8-dihydro-5*H*-pyrazolo[4',3':5,6]pyrido[2,3-*d*]pyrimidine-5,7(6*H*)-dione (10a)](#_Toc38178927) 52

[FT-IR Spectrum of 4-(3,4-dimethoxyphenyl)-3-methyl-1-phenyl-1,8-dihydro-5*H*-pyrazolo[4',3':5,6]pyrido[2,3-*d*]pyrimidine-5,7(6*H*)-dione (11a)](#_Toc38178928) 53

[^1^H-NMR Spectrum of 4-(3,4-dimethoxyphenyl)-3-methyl-1-phenyl-1,8-dihydro-5*H*-pyrazolo[4',3':5,6]pyrido[2,3-*d*]pyrimidine-5,7(6*H*)-dione (11a)](#_Toc38178929) 54

[^13^C-NMR Spectrum of 4-(3,4-dimethoxyphenyl)-3-methyl-1-phenyl-1,8-dihydro-5*H*-pyrazolo[4',3':5,6]pyrido[2,3-*d*]pyrimidine-5,7(6*H*)-dione (11a)](#_Toc38178930) 55

[FT-IR Spectrum of 3-methyl-1-phenyl-4-(thiophen-2-yl)-1,8-dihydro-5*H*-pyrazolo[4',3':5,6]pyrido[2,3-*d*]pyrimidine-5,7(6*H*)-dione (12a)](#_Toc38178931) 56

[^1^H-NMR Spectrum of 3-methyl-1-phenyl-4-(thiophen-2-yl)-1,8-dihydro-5*H*-pyrazolo[4',3':5,6]pyrido[2,3-*d*]pyrimidine-5,7(6*H*)-dione (12a)](#_Toc38178932) 57

[^13^C-NMR Spectrum of 3-methyl-1-phenyl-4-(thiophen-2-yl)-1,8-dihydro-5*H*-pyrazolo[4',3':5,6]pyrido[2,3-*d*]pyrimidine-5,7(6*H*)-dione (12a)](#_Toc38178933) 58

[FT-IR Spectrum of 4-(3,5-difluorophenyl)-3-methyl-1-phenyl-1,8-dihydro-5*H*-pyrazolo[4',3':5,6]pyrido[2,3-*d*]pyrimidine-5,7(6*H*)-dione (13a)](#_Toc38178934) 59

[^1^H-NMR Spectrum of 4-(3,5-difluorophenyl)-3-methyl-1-phenyl-1,8-dihydro-5*H*-pyrazolo[4',3':5,6]pyrido[2,3-*d*]pyrimidine-5,7(6*H*)-dione (13a)](#_Toc38178935) 60

[^13^C-NMR Spectrum of 4-(3,5-difluorophenyl)-3-methyl-1-phenyl-1,8-dihydro-5*H*-pyrazolo[4',3':5,6]pyrido[2,3-*d*]pyrimidine-5,7(6*H*)-dione (13a)](#_Toc38178936) 61

[FT-IR Spectrum of 4-(3,4-difluorophenyl)-3-methyl-1-phenyl-1,8-dihydro-5*H*-pyrazolo[4',3':5,6]pyrido[2,3-*d*]pyrimidine-5,7(6*H*)-dione (14a)](#_Toc38178937) 62

[^1^H-NMR Spectrum of 4-(3,4-difluorophenyl)-3-methyl-1-phenyl-1,8-dihydro-5*H*-pyrazolo[4',3':5,6]pyrido[2,3-*d*]pyrimidine-5,7(6*H*)-dione (14a)](#_Toc38178938) 63

[^13^C-NMR Spectrum of 4-(3,4-difluorophenyl)-3-methyl-1-phenyl-1,8-dihydro-5*H*-pyrazolo[4',3':5,6]pyrido[2,3-*d*]pyrimidine-5,7(6*H*)-dione (14a)](#_Toc38178939) 64

[FT-IR Spectrum of 4-(2,6-dichlorophenyl)-3-methyl-1-phenyl-1,8-dihydro-5*H*-pyrazolo[4',3':5,6]pyrido[2,3-*d*]pyrimidine-5,7(6*H*)-dione (15a)](#_Toc38178940) 65

[^1^H-NMR Spectrum of4-(2,6-dichlorophenyl)-3-methyl-1-phenyl-1,8-dihydro-5*H*-pyrazolo[4',3':5,6]pyrido[2,3-*d*]pyrimidine-5,7(6*H*)-dione (15a)](#_Toc38178941) 66

[^13^C-NMR Spectrum of 4-(2,6-dichlorophenyl)-3-methyl-1-phenyl-1,8-dihydro-5*H*-pyrazolo[4',3':5,6]pyrido[2,3-*d*]pyrimidine-5,7(6*H*)-dione (15a)](#_Toc38178942) 67

[FT-IR Spectrum of 3-methyl-4-(naphthalen-2-yl)-1-phenyl-1,8-dihydro-5*H*-pyrazolo[4',3':5,6]pyrido[2,3-*d*]pyrimidine-5,7(6*H*)-dione (16a)](#_Toc38178943) 68

[^1^H-NMR Spectrum of 3-methyl-4-(naphthalen-2-yl)-1-phenyl-1,8-dihydro-5*H*-pyrazolo[4',3':5,6]pyrido[2,3-*d*]pyrimidine-5,7(6*H*)-dione (16a)](#_Toc38178944) 69

[^13^C-NMR Spectrum of3-methyl-4-(naphthalen-2-yl)-1-phenyl-1,8-dihydro-5*H*-pyrazolo[4',3':5,6]pyrido[2,3-*d*]pyrimidine-5,7(6*H*)-dione (16a)](#_Toc38178945) 70

[FT-IR Spectrum of 3-methyl-1-phenyl-4-(p-tolyl)-1,8-dihydro-5*H*-pyrazolo[4',3':5,6]pyrido[2,3-*d*]pyrimidine-5,7(6*H*)-dion (17a)](#_Toc38178946) 71

[^1^H-NMR Spectrum of 3-methyl-1-phenyl-4-(p-tolyl)-1,8-dihydro-5*H*-pyrazolo[4',3':5,6]pyrido[2,3-*d*]pyrimidine-5,7(6*H*)-dion (17a)](#_Toc38178947) 72

[FT-IR Spectrum of 3-methyl-4-(p-tolyl)-1,4,8,9-tetrahydro-5*H*-pyrazolo[4',3':5,6]pyrido[2,3-*d*]pyrimidine-5,7(6*H*)-dione (1b)](#_Toc38178948) 73

[^1^H-NMR Spectrum of 3-methyl-4-(p-tolyl)-1,4,8,9-tetrahydro-5*H*-pyrazolo[4',3':5,6]pyrido[2,3-*d*]pyrimidine-5,7(6*H*)-dione (1b)](#_Toc38178949) 74

[^13^C-NMR Spectrum of3-methyl-4-(p-tolyl)-1,4,8,9-tetrahydro-5*H*-pyrazolo[4',3':5,6]pyrido[2,3-*d*]pyrimidine-5,7(6*H*)-dione (1b)](#_Toc38178950) 75

[FT-IR Spectrum of 3-methyl-4-(4-nitrophenyl)-1,4,8,9-tetrahydro-5*H-*pyrazolo[4',3':5,6]pyrido[2,3-*d*]pyrimidine-5,7(6*H*)-dione (2b)](#_Toc38178951) 76

[^1^H-NMR Spectrum of 3-methyl-4-(4-nitrophenyl)-1,4,8,9-tetrahydro-5*H*-pyrazolo[4',3':5,6]pyrido[2,3-*d*]pyrimidine-5,7(6*H*)-dione (2b)](#_Toc38178952) 77

[^13^C-NMR Spectrum of 3-methyl-4-(4-nitrophenyl)-1,4,8,9-tetrahydro-5*H*-pyrazolo[4',3':5,6]pyrido[2,3-*d*]pyrimidine-5,7(6*H*)-dione (2b)](#_Toc38178953) 78

[FT-IR Spectrum of 3-methyl-4-(3-nitrophenyl)-1,4,8,9-tetrahydro-5*H*-pyrazolo[4',3':5,6]pyrido[2,3-*d*]pyrimidine-5,7(6*H*)-dione (3b)](#_Toc38178954) 79

[^1^H-NMR Spectrum of 3-methyl-4-(3-nitrophenyl)-1,4,8,9-tetrahydro-5*H*-pyrazolo[4',3':5,6]pyrido[2,3-*d*]pyrimidine-5,7(6*H*)-dione (3b)](#_Toc38178955) 80

[^13^C-NMR Spectrum of 3-methyl-4-(3-nitrophenyl)-1,4,8,9-tetrahydro-5*H*-pyrazolo[4',3':5,6]pyrido[2,3-*d*]pyrimidine-5,7(6*H*)-dione (3b)](#_Toc38178956) 81

[FT-IR Spectrum of 4-(4-hydroxyphenyl)-3-methyl-1,4,8,9-tetrahydro-5*H*-pyrazolo[4',3':5,6]pyrido[2,3-*d*]pyrimidine-5,7(6*H*)-dione (4b)](#_Toc38178957) 82

[^1^H-NMR Spectrum of 4-(4-hydroxyphenyl)-3-methyl-1,4,8,9-tetrahydro-5*H*-pyrazolo[4',3':5,6]pyrido[2,3-*d*]pyrimidine-5,7(6*H*)-dione (4b)](#_Toc38178958) 83

[^13^C-NMR Spectrum of 4-(4-hydroxyphenyl)-3-methyl-1,4,8,9-tetrahydro-5*H*-pyrazolo[4',3':5,6]pyrido[2,3-*d*]pyrimidine-5,7(6*H*)-dione (4b)](#_Toc38178959) 84

[FT-IR Spectrum of 4-(4-isopropylphenyl)-3-methyl-1,4,8,9-tetrahydro-5*H*-pyrazolo[4',3':5,6]pyrido[2,3-*d*]pyrimidine-5,7(6*H*)-dione (5b)](#_Toc38178960) 85

[^1^H-NMR Spectrum of 4-(4-isopropylphenyl)-3-methyl-1,4,8,9-tetrahydro-5*H*-pyrazolo[4',3':5,6]pyrido[2,3-*d*]pyrimidine-5,7(6*H*)-dione (5b)](#_Toc38178961) 86

[^13^C-NMR Spectrum of 4-(4-isopropylphenyl)-3-methyl-1,4,8,9-tetrahydro-5*H*-pyrazolo[4',3':5,6]pyrido[2,3-*d*]pyrimidine-5,7(6*H*)-dione (5b)](#_Toc38178962) 87

[FT-IR Spectrum of 3,6,8-trimethyl-4-(p-tolyl)-1,4,8,9-tetrahydro-5*H*-pyrazolo[4',3':5,6]pyrido[2,3-*d*]pyrimidine-5,7(6*H*)-dione (6b)](#_Toc38178963) 88

[^1^H-NMR Spectrum of 3,6,8-trimethyl-4-(p-tolyl)-1,4,8,9-tetrahydro-5*H*-pyrazolo[4',3':5,6]pyrido[2,3-*d*]pyrimidine-5,7(6*H*)-dione (6b)](#_Toc38178964) 89

[^13^C-NMR Spectrum of 3,6,8-trimethyl-4-(p-tolyl)-1,4,8,9-tetrahydro-5*H*-pyrazolo[4',3':5,6]pyrido[2,3-*d*]pyrimidine-5,7(6*H*)-dione (6b)](#_Toc38178965) 90

[FT-IR Spectrum of 3,6,8-trimethyl-4-(4-nitrophenyl)-1,4,8,9-tetrahydro-5*H*-pyrazolo[4',3':5,6]pyrido[2,3-*d*]pyrimidine-5,7(6*H*)-dione (7)](#_Toc38178966) 91

[^1^H-NMR Spectrum of 3,6,8-trimethyl-4-(4-nitrophenyl)-1,4,8,9-tetrahydro-5*H*-pyrazolo[4',3':5,6]pyrido[2,3-*d*]pyrimidine-5,7(6*H*)-dione (7b)](#_Toc38178967) 92

[^13^C-NMR Spectrum of 3,6,8-trimethyl-4-(4-nitrophenyl)-1,4,8,9-tetrahydro-5*H*-pyrazolo[4',3':5,6]pyrido[2,3-*d*]pyrimidine-5,7(6*H*)-dione (7b)](#_Toc38178968) 93

[FT-IR Spectrum of3,6,8-trimethyl-4-(3-nitrophenyl)-1,4,8,9-tetrahydro-5*H*-pyrazolo[4',3':5,6]pyrido[2,3-*d*]pyrimidine-5,7(6*H*)-dione (8b)](#_Toc38178969) 94

[^1^H-NMR Spectrum of 3,6,8-trimethyl-4-(3-nitrophenyl)-1,4,8,9-tetrahydro-5*H*-pyrazolo[4',3':5,6]pyrido[2,3-*d*]pyrimidine-5,7(6*H*)-dione (8b)](#_Toc38178970) 95

[^13^C-NMR Spectrum of 3,6,8-trimethyl-4-(3-nitrophenyl)-1,4,8,9-tetrahydro-5*H*-pyrazolo[4',3':5,6]pyrido[2,3-*d*]pyrimidine-5,7(6*H*)-dione (8b)](#_Toc38178971) 96

[FT-IR Spectrum of 4-(2,6-dichlorophenyl)-3,6,8-trimethyl-1,4,8,9-tetrahydro-5*H*-pyrazolo[4',3':5,6]pyrido[2,3-*d*]pyrimidine-5,7(6*H*)-dione (9b)](#_Toc38178972) 97

[^1^H-NMR Spectrum of 4-(2,6-dichlorophenyl)-3,6,8-trimethyl-1,4,8,9-tetrahydro-5*H*-pyrazolo[4',3':5,6]pyrido[2,3-*d*]pyrimidine-5,7(6*H*)-dione (9b)](#_Toc38178973) 98

[^13^C-NMR Spectrum of 4-(2,6-dichlorophenyl)-3,6,8-trimethyl-1,4,8,9-tetrahydro-5*H*-pyrazolo[4',3':5,6]pyrido[2,3-*d*]pyrimidine-5,7(6*H*)-dione (9b)](#_Toc38178974) 99

[FT-IR Spectrum of 4-(2-chlorophenyl)-3,6,8-trimethyl-1,8-dihydro-5*H*-pyrazolo[4',3':5,6]pyrido[2,3-*d*]pyrimidine-5,7(6*H*)-dione (10b)](#_Toc38178975) 100

[^1^H-NMR Spectrum of 4-(2-chlorophenyl)-3,6,8-trimethyl-1,8-dihydro-5*H*-pyrazolo[4',3':5,6]pyrido[2,3-*d*]pyrimidine-5,7(6*H*)-dione (10b)](#_Toc38178976) 101

[^13^C-NMR Spectrum of 4-(2-chlorophenyl)-3,6,8-trimethyl-1,8-dihydro-5*H*-pyrazolo[4',3':5,6]pyrido[2,3-*d*]pyrimidine-5,7(6*H*)-dione (10b)](#_Toc38178977) 102

[FT-IR Spectrum of 4-(4-bromophenyl)-3,6,8-trimethyl-1,4,8,9-tetrahydro-5*H*-pyrazolo[4',3':5,6]pyrido[2,3-*d*]pyrimidine-5,7(6*H*)-dione (11b)](#_Toc38178978) 103

[^1^H-NMR Spectrum of 4-(4-bromophenyl)-3,6,8-trimethyl-1,4,8,9-tetrahydro-5*H*-pyrazolo[4',3':5,6]pyrido[2,3-*d*]pyrimidine-5,7(6*H*)-dione (11b)](#_Toc38178979) 104

[^13^C-NMR Spectrum of 4-(4-bromophenyl)-3,6,8-trimethyl-1,4,8,9-tetrahydro-5*H*-pyrazolo[4',3':5,6]pyrido[2,3-*d*]pyrimidine-5,7(6*H*)-dione (11b)](#_Toc38178980) 105

[FT-IR Spectrum of 4,4',4''-(((1,3,5-triazine-2,4,6-triyl)tris(oxy))tris(benzene-4,1-diyl))tris(3-methyl-1-phenyl-1,8-dihydro-5*H*-pyrazolo[4',3':5,6]pyrido[2,3-*d*]pyrimidine-5,7(6*H*)-dione) (1c).](#_Toc38178981) 106

[^1^H-NMR Spectrum of 4,4',4''-(((1,3,5-triazine-2,4,6-triyl)tris(oxy))tris(benzene-4,1-diyl))tris(3-methyl-1-phenyl-1,8-dihydro-5*H*-pyrazolo[4',3':5,6]pyrido[2,3-*d*]pyrimidine-5,7(6*H*)-dione) (1c).](#_Toc38178982) 107

[FT-IR Spectrum of 4,4'-(1,3-phenylene)bis(3-methyl-1-phenyl-1,8-dihydro-5*H*-pyrazolo[4',3':5,6]pyrido[2,3-*d*]pyrimidine-5,7(6*H*)-dione) (2c).](#_Toc38178984) 108

[^1^H-NMR Spectrum of 4,4'-(1,3-phenylene)bis(3-methyl-1-phenyl-1,8-dihydro-5*H*-pyrazolo[4',3':5,6]pyrido[2,3-*d*]pyrimidine-5,7(6*H*)-dione) (2c).](#_Toc38178985) 109

[^13^C-NMR Spectrum of 4,4'-(1,3-phenylene)bis(3-methyl-1-phenyl-1,8-dihydro-5*H*-pyrazolo[4',3':5,6]pyrido[2,3-*d*]pyrimidine-5,7(6*H*)-dione) (2c).](#_Toc38178986) 110

[^1^H-NMR Spectrum of 4-(3,5-dimdthyl-1,7-diphenyl-1,4,7,8-tetrahydrodipyrazolo[3,4:4,3-e]pyridine-4-yl)-2-ethoxyphenol (W1)…………...............................................................................................................................](#_Toc38178987)111


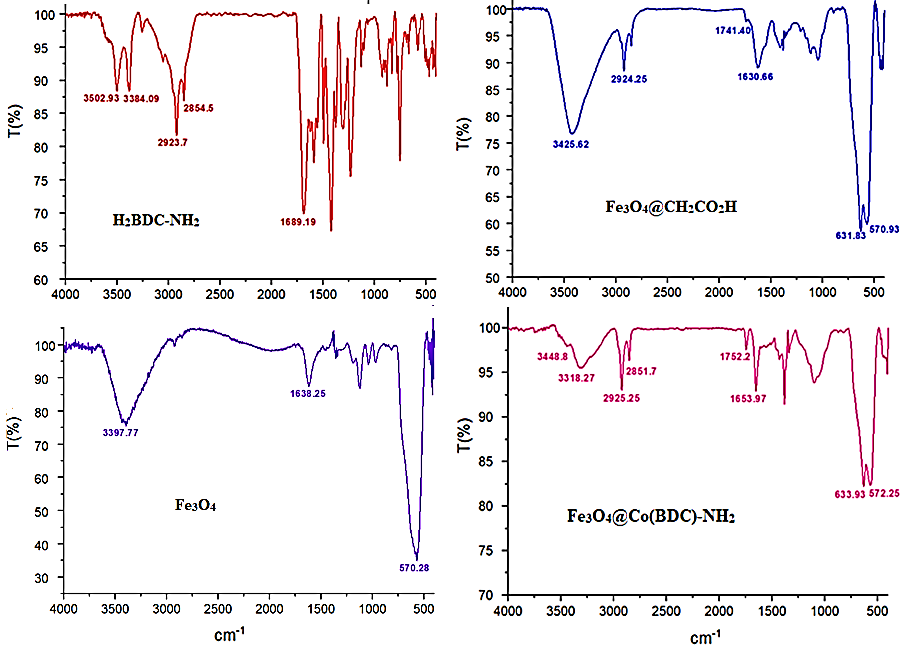


**Figure S1:** FT-IR spectrum of H_2_BDC-NH_2_, Fe_3_O_4_, Fe_3_O_4_@CH_2_CO_2_H, and Fe_3_O_4_@Co(BDC)-NH_2_.


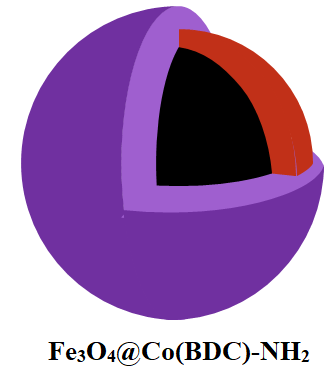

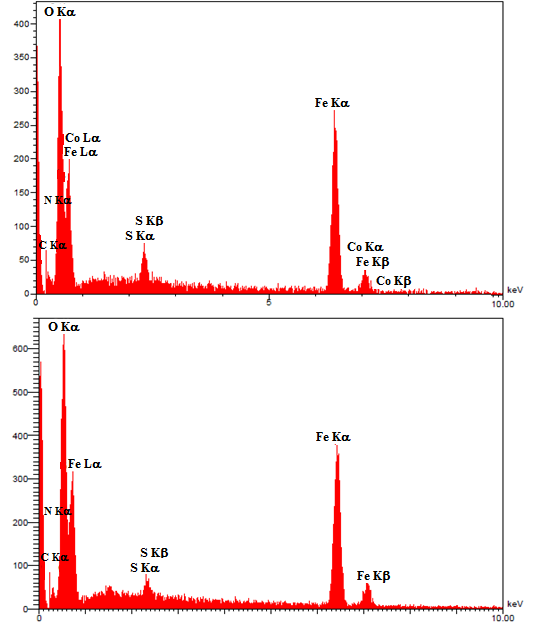


**Figure S2:** Energy dispersive X-ray analysis (EDX) of Fe_3_O_4_@CH_2_CO_2_H and Fe_3_O_4_@Co(BDC)-NH_2_


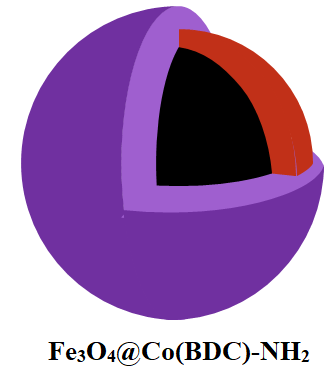

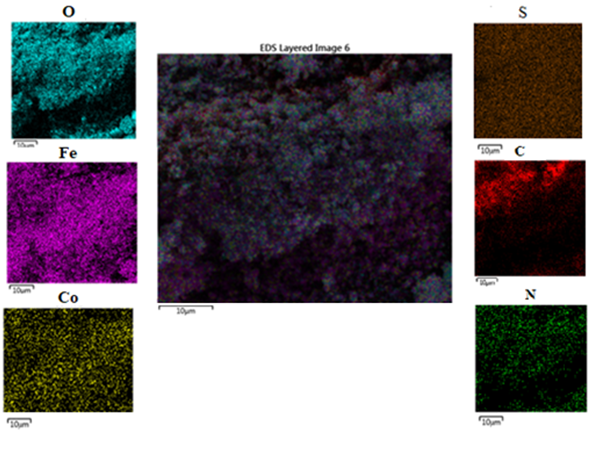


**Figure S3:** Elemental mapping of C (red); O (blue), N (green); Fe (violet) and Co (yellow-orange) atoms for Fe_3_O_4_@Co(BDC)-NH_2_


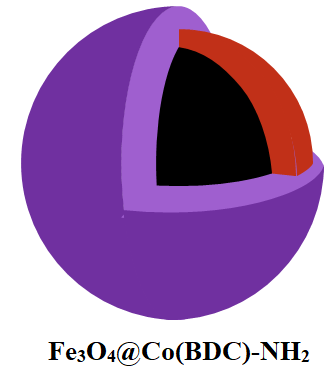

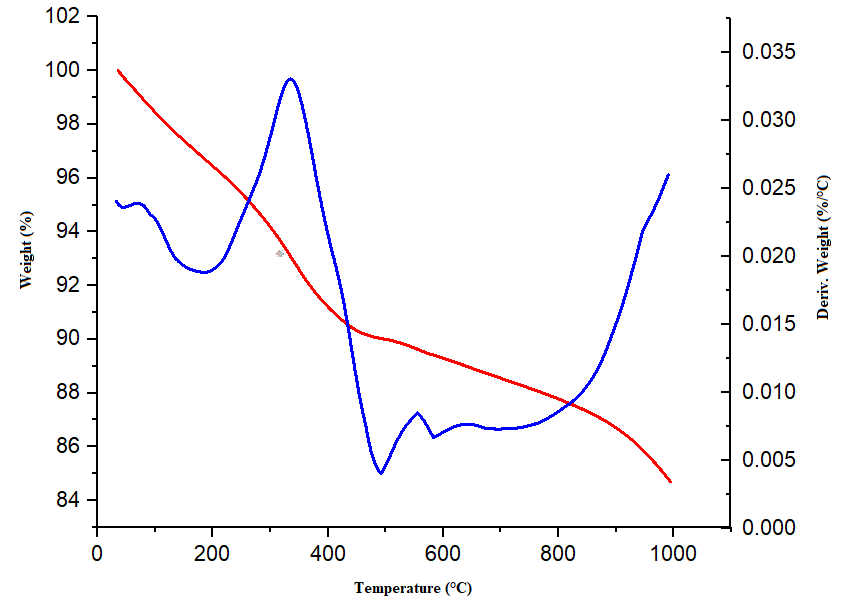


**Figure S4:** Thermal gravimetric (TG) and differential thermal gravimetric (DTG) profile of Fe_3_O_4_@Co(BDC)-NH_2_


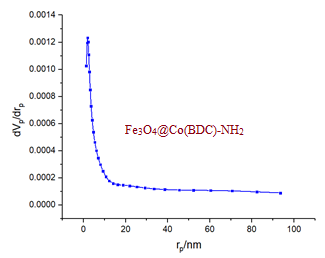

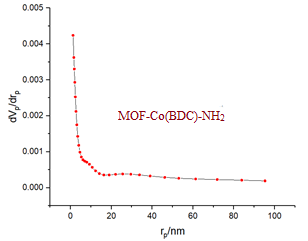


**Figure S5:** BJH of Fe_3_O_4_@Co(BDC)-NH_2_ and MOF-Co(BDC)-NH_2_


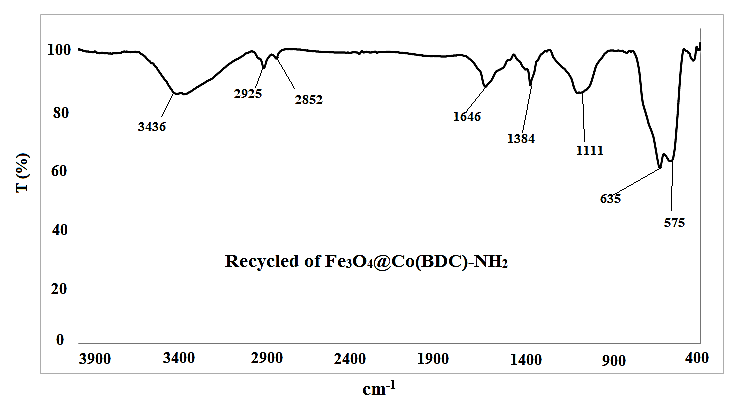


**Figure S6:** FT-IR spectrum recycled of Fe_3_O_4_@Co(BDC)-NH_2_


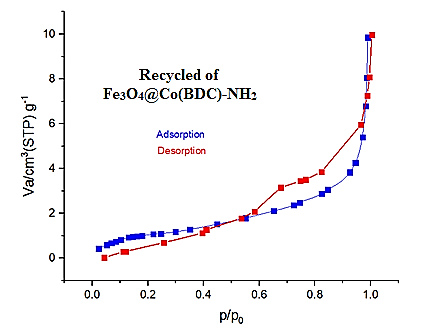


**Figure S7:** Nitrogen adsorption-desorption isotherm (BET) of recycled Fe_3_O_4_@Co(BDC)-NH_2_


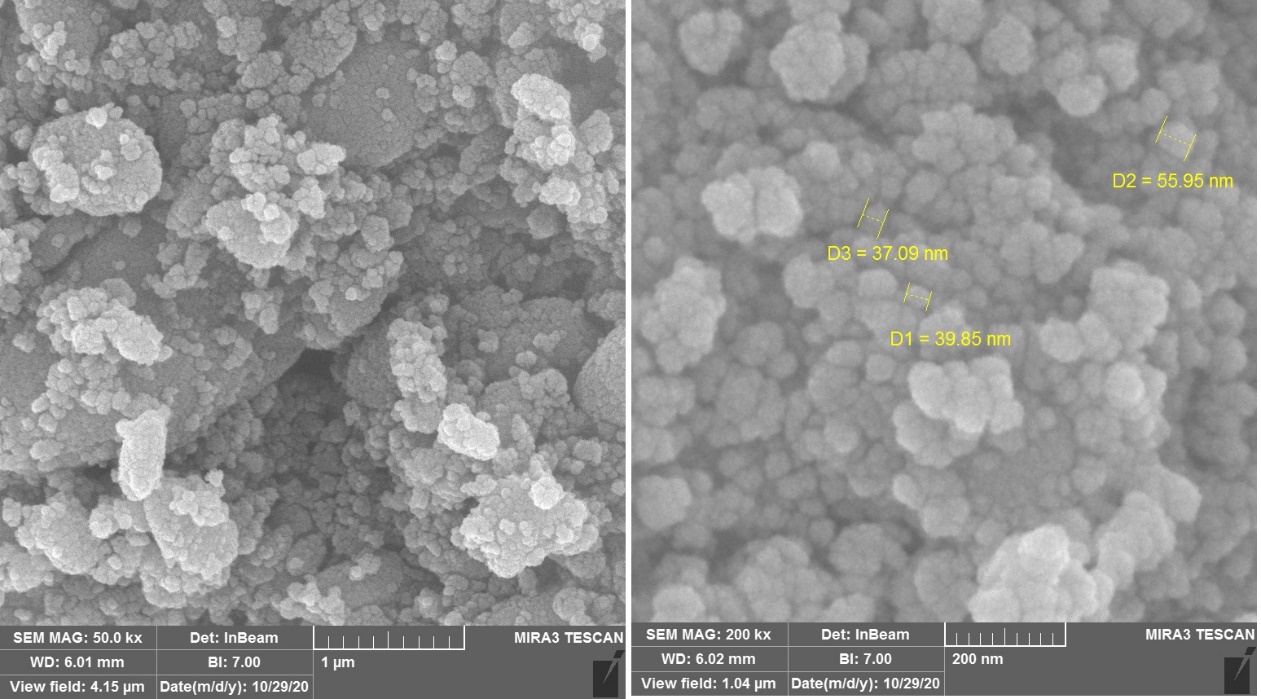


**Figure S8:** Scanning electron microscope (SEM) images of recycled Fe_3_O_4_@Co(BDC)-NH_2_

**Table S1:** XRD data of Fe_3_O_4_@Co(BDC)-NH_2_

| Entry | 2θ | Peak width (degree) | Size [nm] | Inter planer distance [nm] |
| --- | --- | --- | --- | --- |
| 1 | 12.81 | 0.25 | 37.75 | 0.6885 |
| 2 | 25.46 | 0.3 | 26.96 | 0.3486 |
| 3 | 28.51 | 0.3 | 27.13 | 0.3119 |
| 4 | 33.31 | 0.3 | 27.45 | 0.2680 |
| 5 | 35.76 | 0.45 | 18.42 | 0.2501 |
| 6 | 51.26 | 0.5 | 17.50 | 0.1776 |

*3-Methyl-4-(4-nitrophenyl)-1-phenyl-1,8-dihydro-5H-pyrazolo[4',3':5,6]pyrido[2,3-d]pyrimidine-5,7(6H)-dione (1a).*

White solid; Mp: >300 ˚C; FT-IR (KBr, cm^-1^): 3457, 3210, 30780, 1728, 1587; ^1^H NMR (400 MHz, DMSO-*d_6_*): 11.89 (s, 1H), 11.30 (s, 1H), 8.36 (d, *J* = 8.7 Hz, 2H), 8.24 (d, *J* = 7.8 Hz, 2H), 7.68 (d, *J* = 8.7 Hz, 2H), 7.55 (t, *J* = 8.0 Hz, 2H), 7.35 (t, *J* = 7.4 Hz, 1H), 1.75 (s, 3H): ^13^C NMR (100 MHz, DMSO-*d_6_*): δ (ppm): 161.5, 152.6, 150.1, 149.8, 147.9, 147.1, 144.5, 143.4, 138.3, 129.1, 129.1, 126.0, 122.7, 120.3, 112.8, 103.6, 14.0.

*3-Methyl-4-(2-nitrophenyl)-1-phenyl-1,8-dihydro-5H-pyrazolo[4',3':5,6]pyrido[2,3-d]pyrimidine-5,7(6H)-dione (2a).*

Yellow solid; Mp: >300 ˚C; FT-IR (KBr, cm^-1^): 3405, 3069, 2949, 1723, 1702; ^1^H NMR (400 MHz, DMSO-*d_6_*): δ (ppm): 12.03 (s, 1H), 11.43 (s, 1H), 8.44 (dd, *J* = 8.2, 0.9 Hz, 1H), 8.35 (d, *J* = 7.4 Hz, 2H), 7.99 (td, *J* = 7.5, 1.1 Hz, 1H), 7.93 – 7.87 (m, 1H), 7.66 (t, *J* = 8.0 Hz, 2H), 7.61 (dd, *J* = 7.6, 1.2 Hz, 1H), 7.45 (t, *J* = 7.4 Hz, 1H), 1.84 (s, 3H); ^13^C NMR (100 MHz, DMSO-*d_6_*): δ (ppm): 161.9, 152.5, 150.2, 150.1, 147.3, 146.6, 144.4, 138.3, 134.0, 131.4, 130.2, 129.9, 129.2, 126.1, 124.2, 120.4, 112.5, 102.8, 13.4.

*4-(2-Chlorophenyl)-3-methyl-1-phenyl-1,4,8,9-tetrahydro-5H-pyrazolo[4',3':5,6]pyrido[2,3-d]pyrimidine-5,7(6H)-dione* ***(3a).***

White solid; Mp: >300 ˚C; FT-IR (KBr, cm^-1^): 3210, 3067, 3012, 2788, 1719; ^1^H NMR (400 MHz, DMSO-*d_6_*): δ (ppm):10.67 (s, 1H), 7.63 – 7.57 (m, 4H), 7.47 – 7.41 (m, 1H), 7.34 (t, *J* = 7.5 Hz, 2H), 7.26 (t, *J* = 7.0 Hz, 1H), 7.19 (dd, *J* = 7.5, 1.7 Hz, 1H), 5.41 (s, 1H), 1.87 (s, 3H);^13^C NMR (100 MHz, DMSO-*d_6_*): δ (ppm): 163.2, 161.7, 153.2, 150.6, 150.2, 147.4, 146.5, 145.0, 138.9, 138.3, 136.0, 135.4, 132.2, 131.6, 131.2, 130.4, 130.1, 129.6, 129.4, 129.1, 128.0, 127.7, 127.5, 127.2, 126.6, 122.8, 120.9, 113.4, 104.4, 88.5, 13.6, 12.6.

*3-Methyl-4-(3-nitrophenyl)-1-phenyl-1,8-dihydro-5H-pyrazolo[4',3':5,6]pyrido[2,3-d]pyrimidine-5,7(6H)-dione* ***(4a).***

White solid; Mp: >300 ˚C; FT-IR (KBr, cm^-1^): 3417, 3183, 2929, 1714, 1587; ^1^H NMR (400 MHz, DMSO-*d_6_*): δ (ppm): 11.90 (s, 1H), 11.30 (s, 1H), 8.37 (d, *J* = 7.8 Hz, 1H), 8.32 – 8.22 (m, 2H), 7.88 (d, *J* = 7.3 Hz, 1H), 7.81 (t, *J* = 7.8 Hz, 1H), 7.57 (t, *J* = 7.6 Hz, 2H), 7.36 (t, *J* = 7.2 Hz, 1H), 1.78 (s, 3H); ^13^C NMR (100 MHz, DMSO-*d_6_*): δ (ppm):160.8, 155.0, 150.7, 147.2, 145.7, 145.4, 135.4, 128.5, 123.2, 101.1, 86.1, 35.8, 30.1, 27.4, 9.4.

*4-(2-Hydroxy-3-methoxyphenyl)-3-methyl-1-phenyl-1,8-dihydro-5H-pyrazolo[4',3':5,6]pyrido[2,3-d]pyrimidine-5,7(6H)-dione* ***(5a).***

White solid; Mp: >300 ˚C; FT-IR (KBr, cm^-1^): 3382, 3203, 2932, 2811, 1705; ^1^H NMR (400 MHz, DMSO-*d_6_*): δ (ppm):11.78 (s, 1H), 11.17 (s, 1H), 11.03 (s, 1H), 8.72 (s, 1H), 8.26 (d, *J* = 7.7 Hz, 2H), 7.56 (t, *J* = 8.0 Hz, 2H), 7.35 (t, *J* = 7.4 Hz, 1H), 7.10 (t, *J* = 7.9 Hz, 1H), 7.06 (dd, *J* = 8.1, 1.1 Hz,1H), 6.98 (d, *J* = 7.5 Hz, 1H), 6.91 – 6.82 (m, 2H), 6.70 (dd, *J* = 7.7, 1.2 Hz, 1H), 3.88 (s, 3H), 2.09 (s, 3H); ^13^C NMR (100 MHz, DMSO-*d_6_*): δ (ppm): 164.0, 161.1, 153.6, 152.7, 150.2, 149.9, 149.5, 148.2, 147.2, 145.1, 142.6, 138.5, 138.2, 129.1, 125.9, 124.9, 123.3, 120.8, 120.4, 120.3, 118.6, 113.6, 111.4, 110.9, 104.2, 83.6, 55.7, 20.5, 13.1.

*4-(3-Hydroxyphenyl)-3-methyl-1-phenyl-1,8-dihydro-5H-pyrazolo[4',3':5,6]pyrido[2,3-d]pyrimidine-5,7(6H)-dione* ***(6a).***

White solid; Mp: >300 ˚C; FT-IR (KBr, cm^-1^): 3388, 3194, 3078, 2854, 1715; ^1^H NMR (400 MHz, DMSO-*d_6_*): δ (ppm):11.78 (s, 1H), 11.18 (s, 1H), 9.57 (s, 1H), 8.26 (d, 2H), 7.55 (t, 2H), 7.31 (dt, *J* = 15.7, 7.6 Hz, 2H), 6.86 (dd, *J* = 8.2, 1.6 Hz, 1H), 6.71 (dd, *J* = 10.2, 4.8 Hz, 2H), 1.82 (s, 3H);^13^C NMR (100 MHz, DMSO-*d_6_*): δ (ppm): 161.2, 156.5, 152.7, 150.6, 150.2, 149.7, 145.0, 138.5, 137.1, 129.1, 128.7, 125.9, 120.3, 118.1, 114.7, 114.3, 113.4, 103.6, 13.6.

*3-Methyl-1-phenyl-4-(4-(trifluoromethyl)phenyl)-1,8-dihydro-5H-pyrazolo[4',3':5,6]pyrido[2,3-d]pyrimidine-5,7(6H)-dione* ***(7a).***

White solid; Mp: >300 ˚C; FT-IR (KBr, cm^-1^): 3420, 3181, 2929, 2851, 1716; ^1^H NMR (400 MHz, DMSO-*d_6_*): δ (ppm):11.88 (s, 1H), 11.28 (s, 1H), 8.25 (d, *J* = 7.7 Hz, 2H), 7.86 (d, *J* = 8.2 Hz, 2H), 7.61 (d, *J* = 8.0 Hz, 2H), 7.57 (t, *J* = 8.4 Hz, 2H), 7.36 (t, *J* = 7.4 Hz, 2H), 1.75 (s, 3H);^13^C NMR (100 MHz, DMSO-*d_6_*): δ (ppm):161.5, 152.7, 150.2, 149.8, 148.7, 144.6, 140.5, 138.4, 129.2, 128.4, 128.2, 126.0, 124.4, 120.4, 113.1, 103.7, 13.9.

*4-(4-Isopropylphenyl)-3-methyl-1-phenyl-1,8-dihydro-5H-pyrazolo[4',3':5,6]pyrido[2,3-d]pyrimidine-5,7(6H)-dione* ***(8a).***

White solid; Mp: >300 ˚C; FT-IR (KBr, cm^-1^): 3417, 3179, 2926, 2851, 1731; ^1^H NMR (400 MHz, DMSO-*d_6_*): δ (ppm):11.79 (s, 1H), 11.17 (s, 1H), 8.26 (d, *J* = 7.8 Hz, 2H), 7.56 (t, *J* = 7.9 Hz, 2H), 7.35 (d, *J* = 8.1 Hz, 3H), 7.24 (d, *J* = 8.1 Hz, 2H), 3.01 (h, *J* = 6.4 Hz, 1H), 1.74 (s, 3H), 1.29 (d, *J* = 6.9 Hz, 6H);^13^C NMR (100 MHz, DMSO-*d_6_*): δ (ppm): 161.3, 152.7, 150.9, 150.2, 149.7, 147.9, 145.0, 138.5, 133.2, 129.1, 127.4, 125.9, 125.3, 120.3, 113.7, 103.7, 33.1, 23.9, 13.7.

*4-(4-Bromophenyl)-3-methyl-1-phenyl-1,8-dihydro-5H-pyrazolo[4',3':5,6]pyrido[2,3-d]pyrimidine-5,7(6H)-dione* ***(9a).***

White solid; Mp: >300 ˚C; FT-IR (KBr, cm^-1^): 3421, 3219, 2925, 2854, 1716; ^1^H NMR (400 MHz, DMSO-*d_6_*): δ (ppm):11.84 (s, 1H), 11.25 (s, 1H), 8.25 (d, *J* = 7.7 Hz, 2H), 7.69 (d, *J* = 8.3 Hz, 2H), 7.57 (t, *J* = 8.0 Hz, 2H), 7.37 (d, *J* = 7.4 Hz, 1H), 7.33 (d, *J* = 8.3 Hz, 2H), 1.82 (s, 3H);^13^C NMR (100 MHz, DMSO-*d_6_*): δ (ppm): 161.5, 152.7, 150.2, 149.8, 149.2, 144.8, 138.4, 135.2, 130.5, 129.7, 129.2, 126.0, 121.2, 120.4, 113.3, 103.7, 14.1.

*4-(4-Chlorophenyl)-3-methyl-1-phenyl-1,8-dihydro-5H-pyrazolo[4',3':5,6]pyrido[2,3-d]pyrimidine-5,7(6H)-dione* ***(10a).***

White solid; Mp: >300 ˚C; FT-IR (KBr, cm^-1^): 3480, 3222, 2925, 2860, 1719; ^1^H NMR (400 MHz, DMSO-*d_6_*): δ (ppm):11.84 (s, 1H), 11.24 (s, 1H), 8.25 (d, *J* = 7.8 Hz, 2H), 7.56 (t, *J* = 8.0 Hz, 4H), 7.39 (d, *J* = 8.4 Hz, 2H), 7.36 (t, *J* = 7.2 Hz,1H), 1.83 (d, *J* = 19.3 Hz, 3H); ^13^C NMR (100 MHz, DMSO-*d_6_*): δ (ppm): 161.5, 152.7, 150.2, 149.8, 149.2, 144.8, 138.4, 134.8, 132.6, 129.4, 129.1, 127.6, 126.0, 120.4, 113.4, 103.7, 14.1.

*4-(3,4-Dimethoxyphenyl)-3-methyl-1-phenyl-1,8-dihydro-5H-pyrazolo[4',3':5,6]pyrido[2,3-d]pyrimidine-5,7(6H)-dione* ***(11a).***

White solid; Mp: >300 ˚C; FT-IR (KBr, cm^-1^): 3491, 3425, 2924, 1716, 1699; ^1^H NMR (400 MHz, DMSO-*d_6_*): δ (ppm):11.71 (s, 1H), 11.14 (s, 1H), 8.26 (d, *J* = 7.9 Hz, 2H), 7.55 (t, *J* = 7.8 Hz, 2H), 7.34 (t, *J* = 7.2 Hz, 1H), 7.05 (d, *J* = 8.2 Hz, 1H), 6.95 (s, 1H), 6.85 (d, *J* = 7.3 Hz, 1H), 3.84 (s, 3H), 3.73 (s, 3H), 1.85 (s, 3H); ^13^C NMR (100 MHz, DMSO-*d_6_*): δ (ppm): 161.4, 152.9, 150.8, 150.3, 149.8, 148.3, 147.8, 145.2, 138.5, 129.1, 128.1, 125.8, 120.3, 119.9, 113.8, 111.6, 110.6, 103.9, 55.5, 55.3, 14.0.

*3-Methyl-1-phenyl-4-(thiophen-2-yl)-1,8-dihydro-5H-pyrazolo[4',3':5,6]pyrido[2,3-d]pyrimidine-5,7(6H)-dione* ***(12a).***

White solid; Mp: >300 ˚C; FT-IR (KBr, cm^-1^): 3417, 3184, 2923, 1720, 1697; ^1^H NMR (400 MHz, DMSO-*d_6_*): δ (ppm): 11.82 (s, 1H), 11.22 (s, 1H), 8.25 (d, *J* = 7.7 Hz, 2H), 7.79 (dd, *J* = 5.0, 1.1 Hz, 1H), 7.56 (t, *J* = 8.0 Hz, 2H), 7.35 (t, *J* = 7.4 Hz, 1H), 7.20 (dd, *J* = 5.0, 3.5 Hz, 1H), 7.12 (dd, *J* = 3.4, 1.1 Hz, 1H), 1.92 (s, 3H);^13^C NMR (100 MHz, DMSO-*d_6_*): δ (ppm): 179.1, 175.6, 138.4, 132.6, 129.1, 128.2, 127.4, 127.2, 126.6, 126.0, 120.4, 116.1, 114.5, 105.6, 13.2.

*4-(3,5-Difluorophenyl)-3-methyl-1-phenyl-1,8-dihydro-5H-pyrazolo[4',3':5,6]pyrido[2,3-d]pyrimidine-5,7(6H)-dione* ***(13a).***

White solid; Mp: 290-292 ˚C; FT-IR (KBr, cm^-1^): 3446, 3202, 2925, 2854, 1713; ^1^H NMR (400 MHz, DMSO-*d_6_*): δ (ppm): 11.28 (s, 2H), 8.25 (d, *J* = 7.8 Hz, 2H), 7.57 (t, *J* = 8.0 Hz, 2H), 7.42 – 7.34 (m, 2H), 7.18 (d, *J* = 5.7 Hz, 2H), 1.88 (s, 3H);^13^C NMR (100 MHz, DMSO-*d_6_*): δ (ppm): 163.0, 162.9, 161.5, 160.6, 160.5, 153.0, 151.3, 150.9, 150.5, 149.9, 147.3, 144.6, 139.6, 138.4, 129.2, 126.0, 120.4, 112.8, 111.4, 111.2, 103.8, 103.2, 13.8.

*4-(3,4-Difluorophenyl)-3-methyl-1-phenyl-1,8-dihydro-5H-pyrazolo[4',3':5,6]pyrido[2,3-d]pyrimidine-5,7(6H)-dione* ***(14a).***

White solid; Mp: >300 ˚C; FT-IR (KBr, cm^-1^): 3468, 3190, 2929, 1724, 1710; ^1^H NMR (400 MHz, DMSO-*d_6_*): δ (ppm): 11.82 (s, 1H), 11.29 (s, 1H), 8.24 (d, *J* = 7.7 Hz, 2H), 7.62 – 7.51 (m, 4H), 7.35 (t, *J* = 7.4 Hz, 1H), 7.26 – 7.20 (m, 1H), 1.87 (d, *J* = 11.1 Hz, 3H); ^13^C NMR (100 MHz, DMSO-*d_6_*): δ (ppm):161.5, 152.8, 150.3, 149.8, 148.0, 147.8, 147.6, 144.7, 138.3, 133.2, 129.1, 126.0, 124.7, 120.3, 117.3, 117.1, 116.9, 116.7, 113.3, 103.8, 14.0.

*4-(2,6-Dichlorophenyl)-3-methyl-1-phenyl-1,8-dihydro-5H-pyrazolo[4',3':5,6]pyrido[2,3-d]pyrimidine-5,7(6H)-dione* ***(15a).***

White solid; Mp: >300 ˚C; FT-IR (KBr, cm^-1^): 3357, 3078, 2854, 1717, 1694; ^1^H NMR (400 MHz, DMSO-*d_6_*): δ (ppm):12.06 (s, 1H), 11.44 (s, 1H), 8.26 (d, *J* = 7.8 Hz, 2H), 7.67 (d, *J* = 7.6 Hz, 2H), 7.62 – 7.54 (m, 3H), 7.38 (t, *J* = 7.4 Hz, 1H), 1.86 (s, 3H); ^13^C NMR (100 MHz, DMSO-*d_6_*): δ (ppm): 161.1, 153.0, 150.5, 150.1, 144.0, 143.8, 138.2, 133.7, 131.9, 130.9, 129.2, 127.9, 126.3, 120.5, 111.8, 103.6, 12.6.

*3-Methyl-4-(naphthalen-2-yl)-1-phenyl-1,8-dihydro-5H-pyrazolo[4',3':5,6]pyrido[2,3-d]pyrimidine-5,7(6H)-dione* ***(16a).***

White solid; Mp: >300 ˚C; FT-IR (KBr, cm^-1^): 3468, 3423, 3069, 1722, 1702; ^1^H NMR (400 MHz, DMSO-*d_6_*): δ (ppm): ^1^H NMR (400 MHz, DMSO-*d_6_*) δ 11.85 (s, 1H), 11.16 (s, 1H), 8.28 (d, *J* = 7.5 Hz, 2H), 8.00 (dd, *J* = 13.6, 7.4 Hz, 3H), 7.89 (s, 1H), 7.65 – 7.53 (m, 4H), 7.51 (d, *J* = 8.0 Hz, 1H), 7.42 – 7.31 (m, 1H), 1.68 (s, 3H); ^13^C NMR (100 MHz, DMSO-*d_6_*): δ (ppm): 161.6, 150.5, 149.9, 144.9, 140.0, 138.5, 133.6, 132.3, 132.1, 129.1, 127.9, 127.6, 126.7, 126.4, 126.1, 126.0, 120.4, 115.0, 113.6, 103.9, 14.0.

*3-Methyl-1-phenyl-4-(p-tolyl)-1,8-dihydro-5H-pyrazolo[4',3':5,6]pyrido[2,3-d]pyrimidine-5,7(6H)-dione****(17a).*** *and 3-Methyl-1-phenyl-4-(p-tolyl)-1,4,8,9-tetrahydro-5H-pyrazolo[4',3':5,6]pyrido[2,3-d]pyrimidine-5,7(6H)-dione* ***(17a).***

White solid; Mp: >300 ˚C; FT-IR (KBr, cm^-1^): 3185, 3049, 2926, 2857, 1717; ^1^H NMR (400 MHz, DMSO-*d_6_*): δ (ppm): 11.86 (s, 1H), 11.24 (s, 1H), 10.74 (s, 1H), 10.13 (s, 1H), 9.04 (s, 1H), 8.35 (d, *J* = 7.7 Hz, 1H), 7.68 – 7.62 (m, 5H), 7.54 – 7.47 (m, *J* = 8.6, 3.3 Hz, 1H), 7.44 (t, *J* = 7.4 Hz, 1H), 7.38 (d, *J* = 7.8 Hz, 1H), 7.31 (d, *J* = 8.0 Hz, 1H), 7.23 (d, *J* = 8.0 Hz, 2H), 7.14 (d, *J* = 7.9 Hz, 2H), 4.99 (s, 1H), 2.51 (s, 2H), 2.32 (s, 4H), 1.97 (s, 4H), 1.87 (s, 2H).

*3-Methyl-4-(p-tolyl)-1,4,8,9-tetrahydro-5H-pyrazolo[4',3':5,6]pyrido[2,3-d]pyrimidine-5,7(6H)-dione* ***(1b).***

White solid; Mp: >300 ˚C; FT-IR (KBr, cm^-1^): 3341, 3221, 2923, 1726; ^1^H NMR (400 MHz, DMSO-*d_6_*): δ (ppm): 11.90 (s, 1H), 10.54 (s, 1H), 10.03 (s, 1H), 8.81 (s, 1H), 7.11 (q, *J* = 8.1 Hz, 4H), 4.92 (s, 1H), 2.30 (s, 3H), 1.98 (s, 3H);^13^C NMR (100 MHz, DMSO-*d_6_*): δ (ppm): 162.9, 150.0, 146.7, 145.2, 144.3, 135.1, 134.4, 128.3, 127.1, 102.4, 86.7, 34.1, 20.5, 9.4.

*3-Methyl-4-(4-nitrophenyl)-1,4,8,9-tetrahydro-5H-pyrazolo[4',3':5,6]pyrido[2,3-d]pyrimidine-5,7(6H)-dione* ***(2b).***

White solid; Mp: >300 ˚C; FT-IR (KBr, cm^-1^): 3468, 3345, 3091, 1723, 1709; ^1^H NMR (400 MHz, DMSO-*d_6_*): δ (ppm): 11.96 (s, 1H), 10.57 (s, 1H), 10.08 (s, 1H), 8.92 (s, 1H), 8.11 (d, *J* = 8.7 Hz, 2H), 7.46 (d, *J* = 8.7 Hz, 2H), 5.07 (s, 1H), 1.89 (s, 3H); ^13^C NMR (100 MHz, DMSO-*d_6_*): δ (ppm): 162.9, 154.7, 149.9, 147.1, 145.5, 145.0, 135.8, 128.5, 123.3, 100.9, 85.6, 34.8, 9.4.

*3-Methyl-4-(3-nitrophenyl)-1,4,8,9-tetrahydro-5H-pyrazolo[4',3':5,6]pyrido[2,3-d]pyrimidine-5,7(6H)-dione* ***(3b).***

White solid; Mp: >300 ˚C; FT-IR (KBr, cm^-1^): 3402, 3216, 2917, 1701, 1657; ^1^H NMR (400 MHz, DMSO-*d_6_*): δ (ppm):11.97 (s, 1H), 10.57 (s, 1H), 10.09 (s, 1H), 8.94 (s, 1H), 8.00 (d, *J* = 6.7 Hz, 2H), 7.66 (d, *J* = 7.7 Hz, 1H), 7.53 (t, *J* = 8.2 Hz, 1H), 5.11 (s, 1H), 1.88 (s, 3H);^13^C NMR (100 MHz, DMSO-*d_6_*): δ (ppm):163.0, 150.0, 149.3, 147.4, 147.1, 145.15 135.8, 134.2, 129.4, 121.6, 120., 101.1, 85.7, 34.5, 9.3.

*4-(4-Hydroxyphenyl)-3-methyl-1,4,8,9-tetrahydro-5H-pyrazolo[4',3':5,6]pyrido[2,3-d]pyrimidine-5,7(6H)-dione* ***(4b).***

White solid; Mp: >300 ˚C; FT-IR (KBr, cm^-1^): 3393, 3239, 2903, 2814, 1702; ^1^H NMR (400 MHz, DMSO-*d_6_*): δ (ppm): 11.81 (s, 1H), 10.47 (s, 1H), 9.93 (s, 1H), 9.08 (s, 1H), 8.69 (s, 1H), 6.94 (d, *J* = 8.5 Hz, 2H), 6.58 (d, *J* = 8.5 Hz, 2H), 4.77 (s, 1H), 1.90 (s, 3H); ^13^C NMR (100 MHz, DMSO-*d_6_*): δ (ppm): 163.0, 155.0, 150.0, 146.5, 145.1, 137.8, 135.2, 128.0, 114.5, 102.8, 87.0, 33.6, 9.3.

*4-(4-Isopropylphenyl)-3-methyl-1,4,8,9-tetrahydro-5H-pyrazolo[4',3':5,6]pyrido[2,3-d]pyrimidine-5,7(6H)-dione* ***(5b).***

White solid; Mp: >300 ˚C; FT-IR (KBr, cm^-1^): 3342, 3221, 3044, 2877, 1721; ^1^H NMR (400 MHz, DMSO-*d_6_*): δ (ppm): 11.84 (s, 1H), 10.49 (s, 1H), 9.98 (s, 1H), 8.74 (s, 1H), 7.07 (s, 4H), 4.85 (s, 1H), 2.85 – 2.73 (m, 1H), 1.91 (s, 3H), 1.15 (d, *J* = 6.9 Hz, 6H); ^13^C NMR (100 MHz, DMSO-*d_6_*): δ (ppm): 162.9, 150.0, 146.8, 145.4, 144.6, 135.2, 126.9, 125.7, 102.5, 86.7, 34.0, 32.9, 23.9, 23.8, 9.4.

*3,6,8-Trimethyl-4-(p-tolyl)-1,4,8,9-tetrahydro-5H-pyrazolo[4',3':5,6]pyrido[2,3-d]pyrimidine-5,7(6H)-dione* ***(6b).***

White solid; Mp: >300 ˚C; FT-IR (KBr, cm^-1^): 3319, 3226, 2952, 2895, 1697; ^1^H NMR (400 MHz, DMSO-*d_6_*): δ (ppm): 12.00 (s, 1H), 9.85 (s, 1H), 7.15 (d, *J* = 8.0 Hz, 2H), 7.08 (d, *J* = 8.0 Hz, 2H), 5.04 (s, 1H), 3.55 (s, 3H), 3.15 (s, 3H), 2.29 (s, 3H), 2.01 (s, 3H); ^13^C NMR (100 MHz, DMSO-*d_6_*): δ (ppm): 160.7, 150.7, 146.7, 145.9, 144.6, 134.7, 134.3, 128.3, 127.1, 102.6, 87.3, 35.1, 30.0, 27.4, 20.5, 9.4.

*3,6,8-Trimethyl-4-(4-nitrophenyl)-1,4,8,9-tetrahydro-5H-pyrazolo[4',3':5,6]pyrido[2,3-d]pyrimidine-5,7(6H)-dione* ***(7b).***

White solid; Mp: >300 ˚C; FT-IR (KBr, cm^-1^): 3566, 3252, 3081, 1711, 1697; ^1^H NMR (400 MHz, DMSO-*d_6_*): δ (ppm):12.05 (s, 1H), 9.95 (s, 1H), 8.10 (d, *J* = 8.7 Hz, 2H), 7.48 (d, *J* = 8.7 Hz, 2H), 5.18 (s, 1H), 3.48 (s, 3H), 3.05 (s, 3H), 1.91 (s, 3H); ^13^C NMR (100 MHz, DMSO-*d_6_*): δ (ppm):160.8, 155.0, 150.7, 147.2, 145.7, 145.4, 135.4, 128.5, 123.2, 101.0, 86.1, 35.8, 30.11, 27.4, 9.3.

*3,6,8-Trimethyl-4-(3-nitrophenyl)-1,4,8,9-tetrahydro-5H-pyrazolo[4',3':5,6]pyrido[2,3-d]pyrimidine-5,7(6H)-dione* ***(8b).***

White solid; Mp: 225-227 ˚C; FT-IR (KBr, cm^-1^): 3554, 3220, 3084, 2926, 1686; ^1^H NMR (400 MHz, DMSO-*d_6_*): δ (ppm):12.06 (s, 1H), 9.95 (s, 1H), 8.03 (t, *J* = 2 Hz, 1H), 7.98 (ddd, *J* = 8.1, 2.3, 1.0 Hz, 1H), 7.70 – 7.66 (m, 1H), 7.51 (t, *J* = 7.9 Hz, 1H), 5.20 (s, 1H), 3.47 (s, 3H), 3.05 (s, 3H), 1.89 (s, 3H);^13^C NMR (100 MHz, DMSO-*d_6_*): δ (ppm): 160.9, 150.7, 149.5, 147.4, 147.1, 145.8, 134.3, 129.3, 121.7, 120.8, 101.3, 86.3, 35.5, 30.1, 27.4, 9.3.

*4-(2,6-Dichlorophenyl)-3,6,8-trimethyl-1,4,8,9-tetrahydro-5H-pyrazolo[4',3':5,6]pyrido[2,3-d]pyrimidine-5,7(6H)-dione* ***(9b).***

White solid; Mp: >300 ˚C; FT-IR (KBr, cm^-1^): 3552, 3471, 3221, 2957, 1686; ^1^H NMR (400 MHz, DMSO-*d_6_*): δ (ppm):11.98 (s, 1H), 9.94 (s, 1H), 7.44 (dd, *J* = 7.8, 1.4 Hz, 1H), 7.24 (dd, *J* = 8.0, 1.3 Hz, 1H), 7.18 (t, *J* = 7.9 Hz, 1H), 5.92 (s, 1H), 3.36 (s, 3H), 3.04 (s, 3H), 1.82 (s, 3H); ^13^C NMR (100 MHz, DMSO-*d_6_*): δ (ppm):160.4, 150.6, 147.9, 147.0, 138.6, 135.1, 134.7, 134.4, 130.1, 128.0, 127.8, 98.6, 84.3, 32.8, 30.0, 27.3, 9.1.

*4-(2-Chlorophenyl)-3,6,8-trimethyl-1,8-dihydro-5H-pyrazolo[4',3':5,6]pyrido[2,3-d]pyrimidine-5,7(6H)-dione* ***(10b).***

White solid; Mp: 256-258 ˚C; FT-IR (KBr, cm^-1^): 3148, 3120, 2959, 2852, 1720; ^1^H NMR (400 MHz, DMSO-*d_6_*): δ (ppm): 13.66 (s, 1H), 7.60 (dd, *J* = 7.9, 0.9 Hz, 1H), 7.51 (td, *J* = 7.7, 1.7 Hz, 1H), 7.45 (td, *J* = 7.4, 1.2 Hz, 1H), 7.30 (dd, *J* = 7.5, 1.6 Hz, 1H), 3.66 (s, 3H), 3.19 (s, 3H), 1.72 (s, 3H); ^13^C NMR (100 MHz, DMSO-*d_6_*): δ (ppm): 160.0, 151.9, 150.8, 150.7, 146.8, 135.9, 130.5, 129.5, 128.9, 128.6, 126.7, 110.6, 103.1, 30.0, 27.9, 13.0, 12.9.

*4-(4-Bromophenyl)-3,6,8-trimethyl-1,4,8,9-tetrahydro-5H-pyrazolo[4',3':5,6]pyrido[2,3-d]pyrimidine-5,7(6H)-dione* ***(11b).***

White solid; Mp: 289-291 ˚C; FT-IR (KBr, cm^-1^): 3313, 3221, 2924, 2857, 1698; ^1^H NMR (400 MHz, DMSO-*d_6_*): δ (ppm): 12.00 (s, 1H), 9.85 (s, 1H), 7.39 (d, *J* = 8.4 Hz, 2H), 7.15 (d, *J* = 8.4 Hz, 2H), 5.00 (s, 1H), 3.46 (s, 3H), 3.06 (s, 3H), 1.92 (s, 3H);^13^C NMR (100 MHz, DMSO-*d_6_*): δ (ppm): 160.8, 150.7, 146.8, 136.3, 130.6, 130.5, 129.5, 129.4, 120.9, 118.5, 111.0, 103.0, 101.8, 86.7, 35.1, 30.0, 27.9, 27.4.

*4,4',4''-(((1,3,5-Triazine-2,4,6-triyl)tris(oxy))tris(benzene-4,1-diyl))tris(3-methyl-1-phenyl-1,8-dihydro-5H-pyrazolo[4',3':5,6]pyrido[2,3-d]pyrimidine-5,7(6H)-dione)* ***(1c).***

Brown solid; Mp: >300 ˚C; FT-IR (KBr, cm^-1^): 3509, 3405, 3170, 2924, 1720; ^1^H NMR (400 MHz, DMSO-*d_6_*): δ (ppm): 11.81 (s, 1H), 11.20 (d, *J* = 18.5 Hz, 1H), 8.35 – 8.15 (m, 2H), 7.68 – 7.24 (m, *J* = 24.5, 16.6 Hz, 7H), 1.85 (d, 3H).

*4,4'-(1,3-Phenylene)bis(3-methyl-1-phenyl-1,8-dihydro-5H-pyrazolo[4',3':5,6]pyrido[2,3-d]pyrimidine-5,7(6H)-dione)* ***(2c).***

White solid; Mp: >300 ˚C; FT-IR (KBr, cm^-1^): 3416, 3207, 2925, 2854, 1726, 1702; ^1^H NMR (400 MHz, DMSO-*d_6_*): δ (ppm):11.82 (s, 1H), 11.19 (s, 1H), 8.25 (d, *J* = 7.7 Hz, 2H), 7.66 – 7.60 (m, 1H), 7.56 (t, *J* = 7.9 Hz, 2H), 7.46 (dd, *J* = 7.6, 1.4 Hz, 1H), 7.35 (d, *J* = 14.7 Hz, 1H), 7.29 (t, *J* = 10.4 Hz, 1H), 2.07 (s, 3H); ^13^C NMR (100 MHz, DMSO-*d_6_*): δ (ppm): 172.1, 166.9, 148.9, 145.3, 144.2, 139.4, 136.4, 135.6, 128.9, 128.8, 128.4, 127.9, 127.9, 125.2, 120.9, 102.0, 13.7.

*^1^H-NMR Spectrum of 4-(3,5-dimdthyl-1,7-diphenyl-1,4,7,8-tetrahydrodipyrazolo[3,4:4,3-e]pyridine-4-yl)-2-ethoxyphenol* ***(W1).***

White solid; Mp: >300 ˚C ; ^1^H NMR (400 MHz, DMSO-*d*_6_) δ 8.92 (s, 1H), 7.65 (d, *J* = 7.5 Hz, 4H), 7.53 (t, *J* = 7.9 Hz, 4H), 7.35 (t, *J* = 7.4 Hz, 2H), 6.90 – 6.83 (m, 2H), 6.70 (d, *J* = 6.6 Hz, 1H), 5.29 (s, 1H), 4.04 (q, *J* = 6.9 Hz, 2H), 1.96 (s, 6H), 1.39 (s, 3H).

*
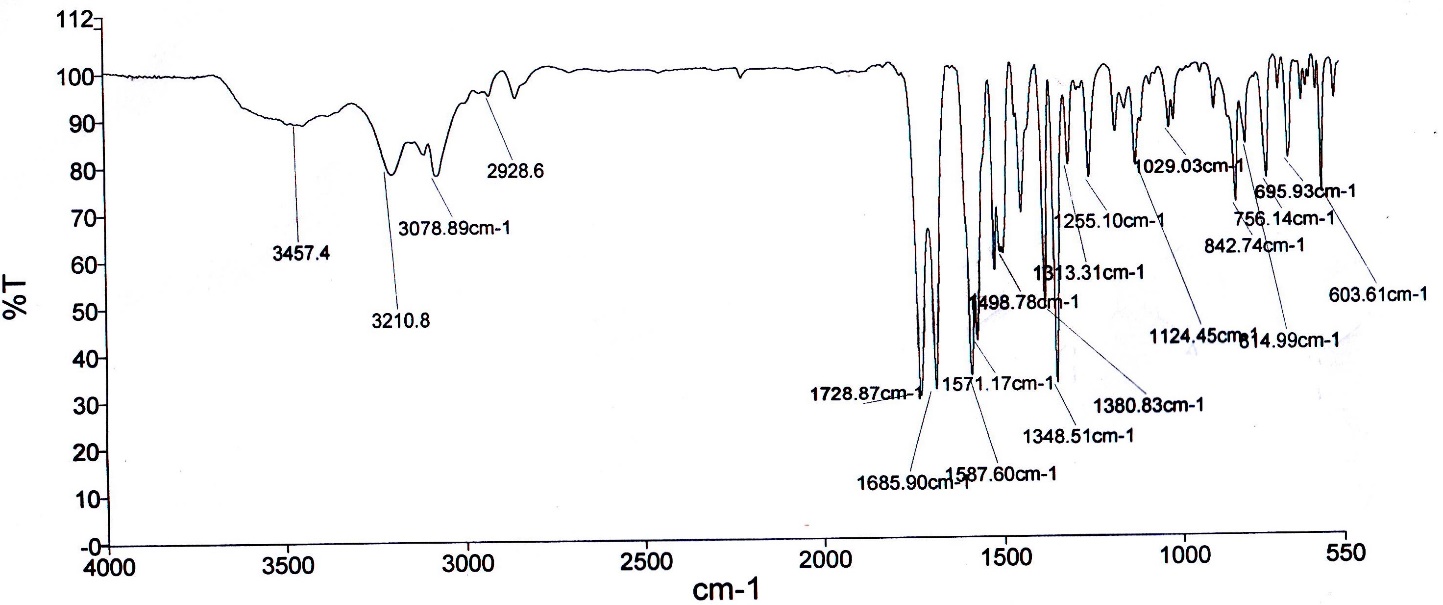
*

FT-IR Spectrum of 3-methyl-4-(4-nitrophenyl)-1-phenyl-1,8-dihydro-5H-pyrazolo[4',3':5,6]pyrido[2,3-d]pyrimidine-5,7(6H)-dione


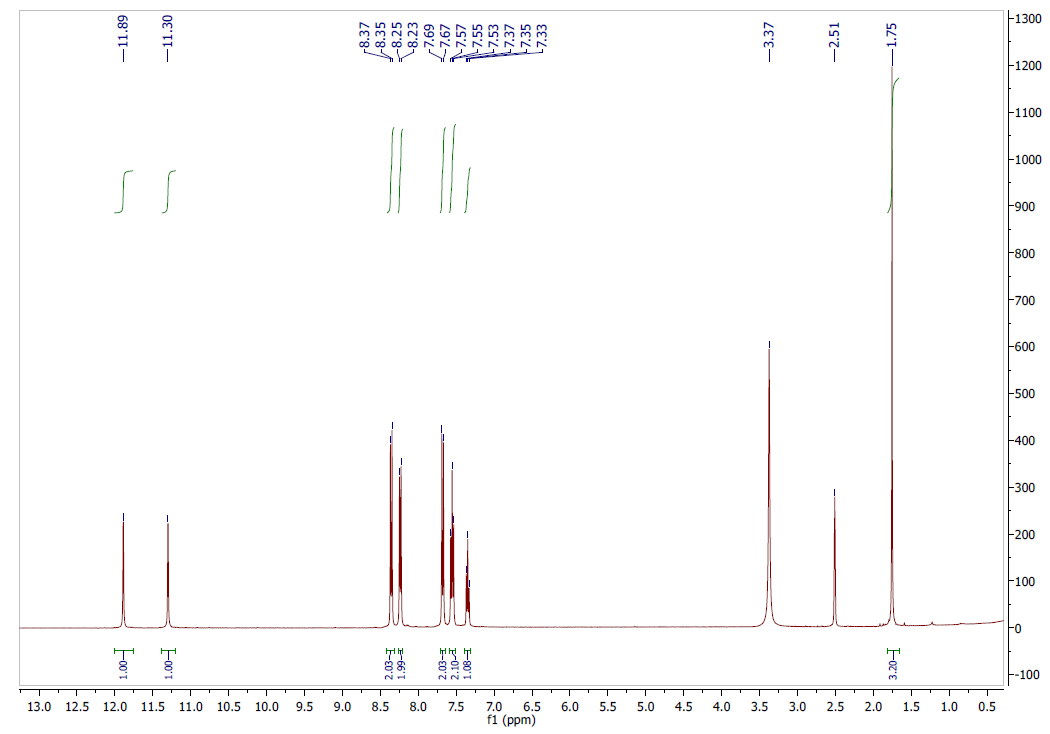
^1^H-NMR Spectrum of 3-methyl-4-(4-nitrophenyl)-1-phenyl-1,8-dihydro-5H-pyrazolo[4',3':5,6]pyrido[2,3-d]pyrimidine-5,7(6H)-dione


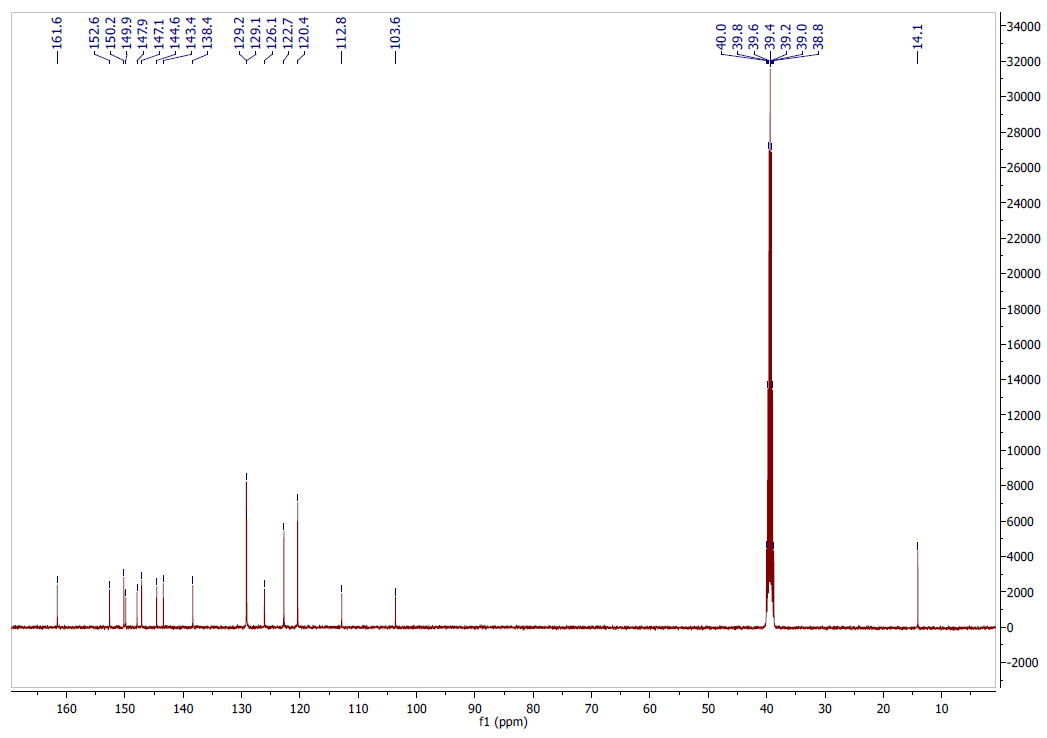
^13^C-NMR Spectrum of 3-methyl-4-(4-nitrophenyl)-1-phenyl-1,8-dihydro-5H-pyrazolo[4',3':5,6]pyrido[2,3-d]pyrimidine-5,7(6H)-dione

*
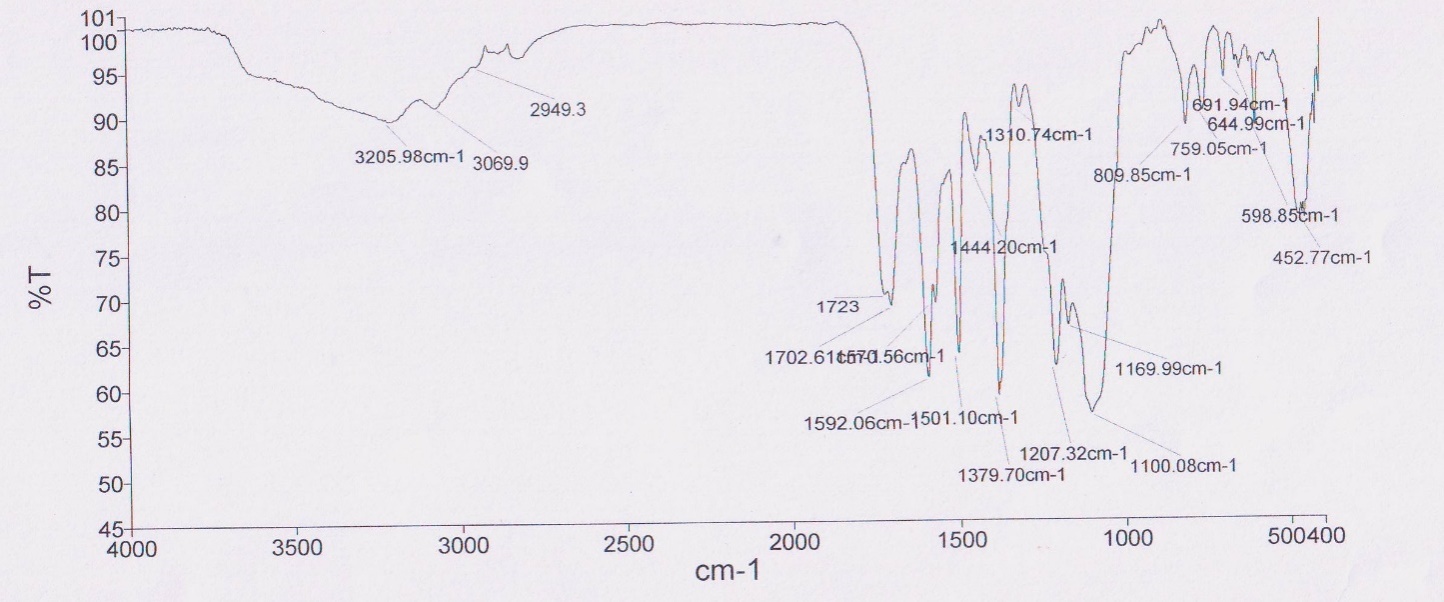
*

FT-IR Spectrum of 3-methyl-4-(2-nitrophenyl)-1-phenyl-1,8-dihydro-5H-pyrazolo[4',3':5,6]pyrido[2,3-d]pyrimidine-5,7(6H)-dione


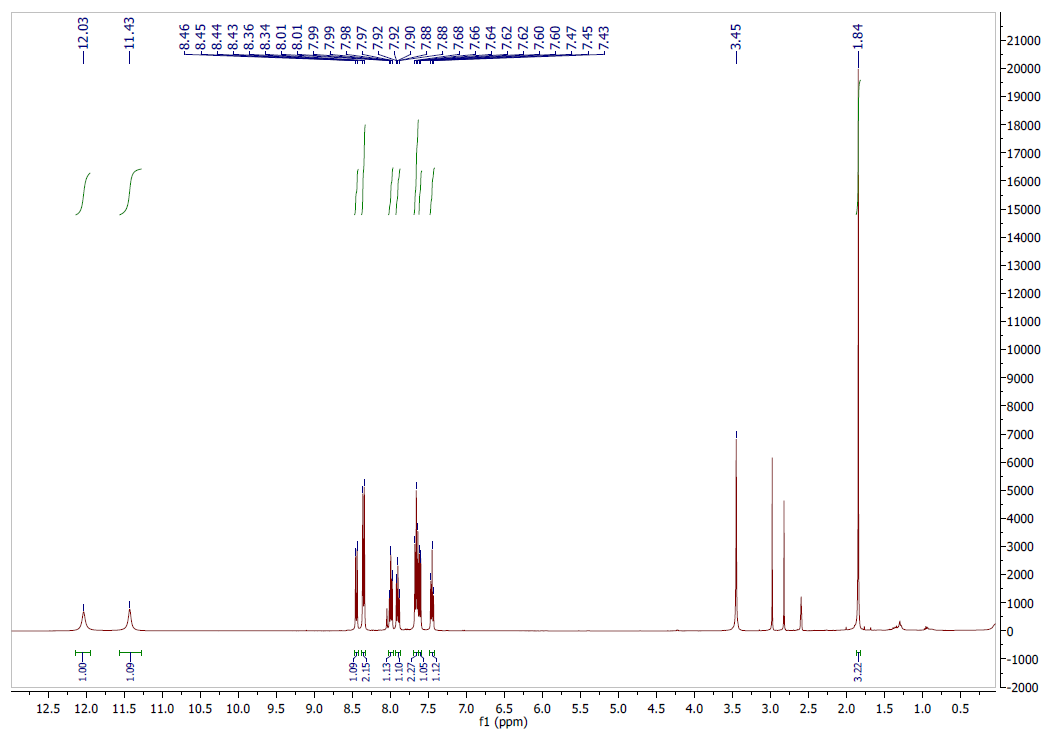
^1^H-NMR Spectrum of 3-methyl-4-(2-nitrophenyl)-1-phenyl-1,8-dihydro-5H-pyrazolo[4',3':5,6]pyrido[2,3-d]pyrimidine-5,7(6H)-dione


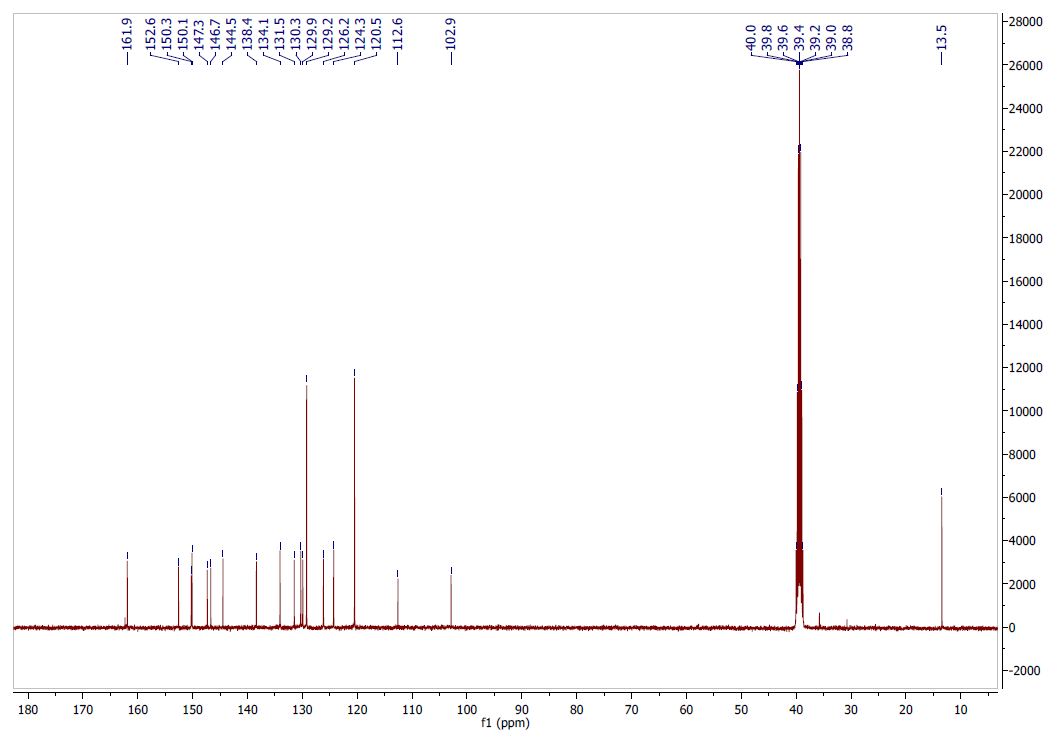
^13^C-NMR Spectrum of 3-methyl-4-(2-nitrophenyl)-1-phenyl-1,8-dihydro-5H-pyrazolo[4',3':5,6]pyrido[2,3-d]pyrimidine-5,7(6H)-dione

*
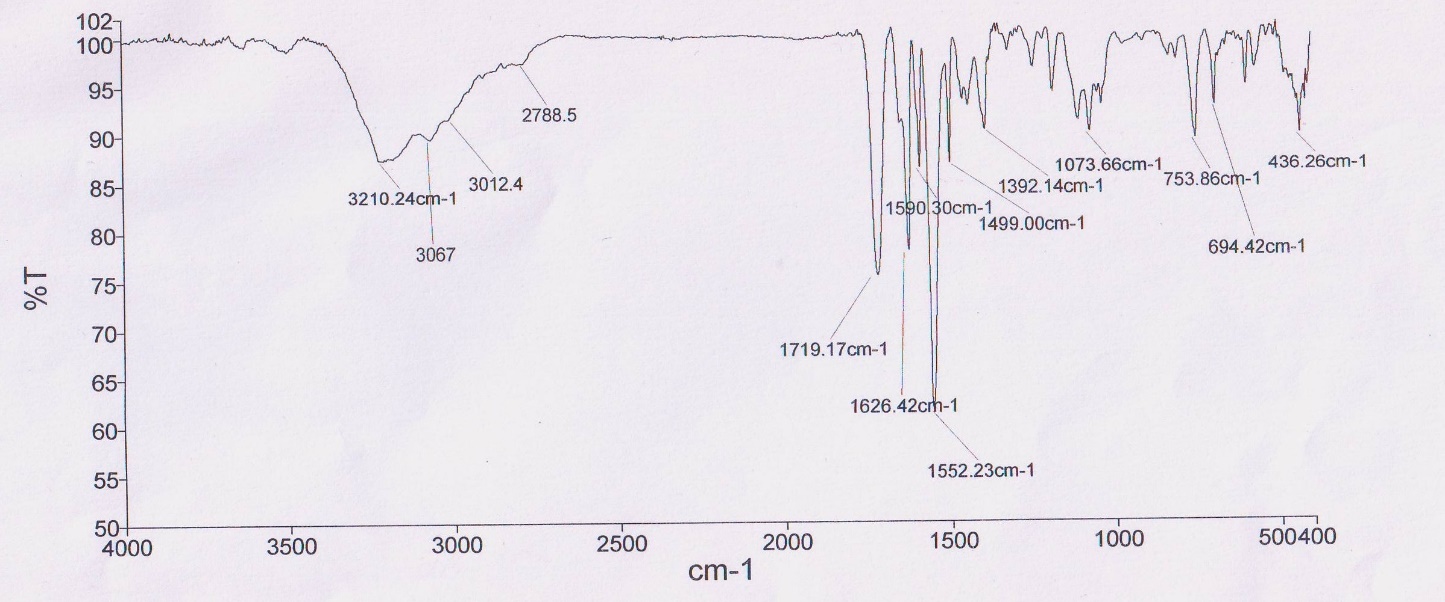
*

FT-IR Spectrum of 4-(2-chlorophenyl)-3-methyl-1-phenyl-1,4,8,9-tetrahydro-5H-pyrazolo[4',3':5,6]pyrido[2,3-d]pyrimidine-5,7(6H)-dione

*
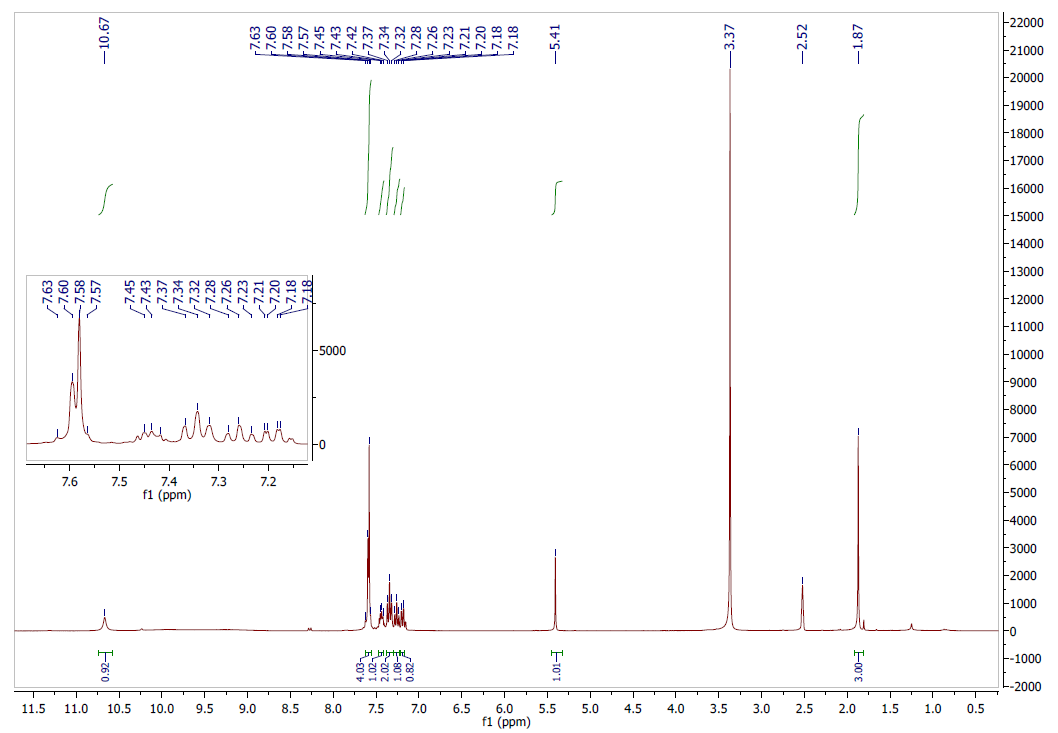
*

^1^H-NMR Spectrum of 4-(2-chlorophenyl)-3-methyl-1-phenyl-1,4,8,9-tetrahydro-5H-pyrazolo[4',3':5,6]pyrido[2,3-d]pyrimidine-5,7(6H)-dione

*
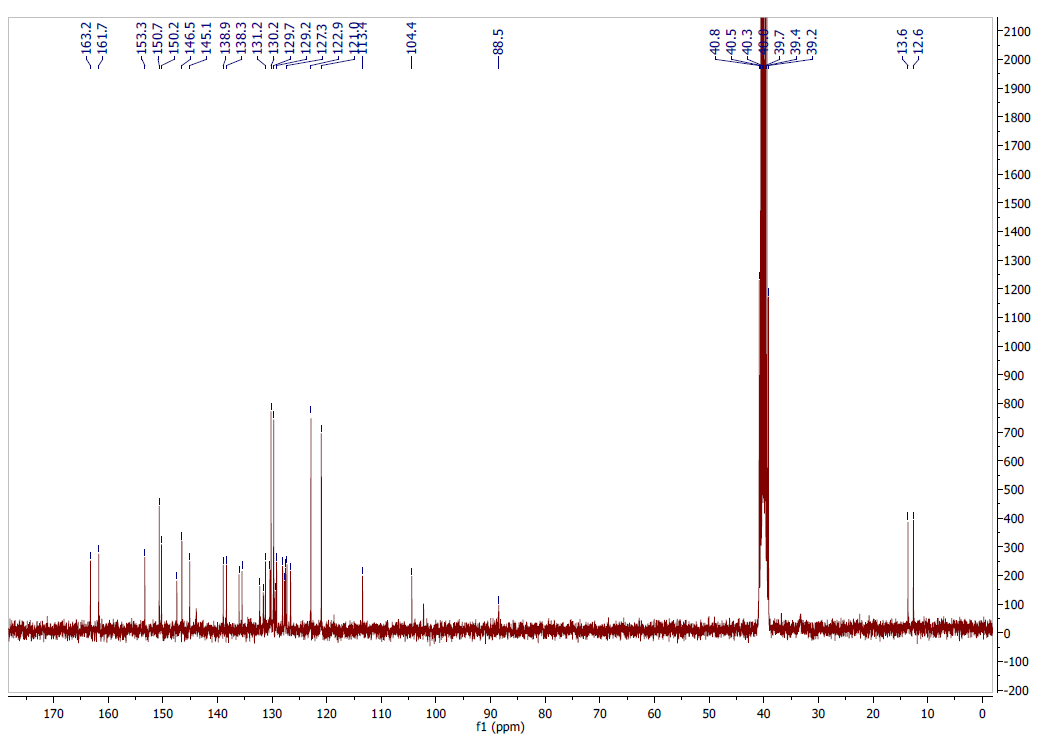
*

^13^C-NMR Spectrum of 4-(2-chlorophenyl)-3-methyl-1-phenyl-1,4,8,9-tetrahydro-5H-pyrazolo[4',3':5,6]pyrido[2,3-d]pyrimidine-5,7(6H)-dione

*
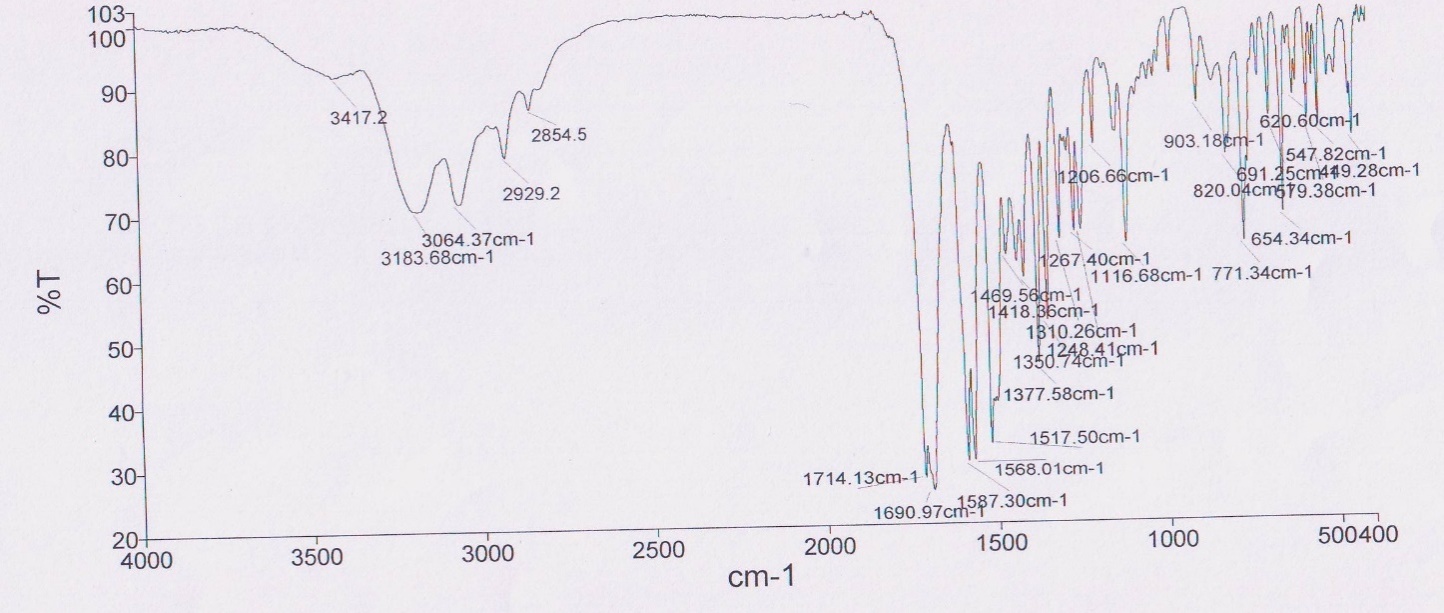
*

FT-IR Spectrum of 3-methyl-4-(3-nitrophenyl)-1-phenyl-1,8-dihydro-5H-pyrazolo[4',3':5,6]pyrido[2,3-d]pyrimidine-5,7(6H)-dione

*
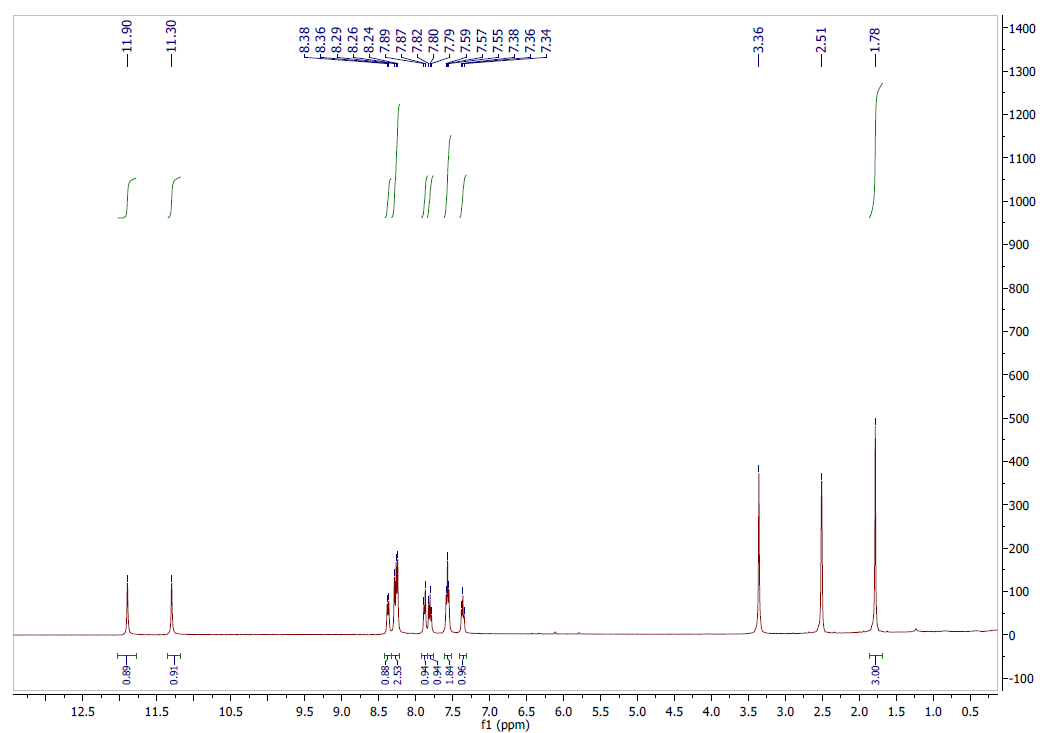
*

^1^H-NMR Spectrum of 3-methyl-4-(3-nitrophenyl)-1-phenyl-1,8-dihydro-5H-pyrazolo[4',3':5,6]pyrido[2,3-d]pyrimidine-5,7(6H)-dione

*
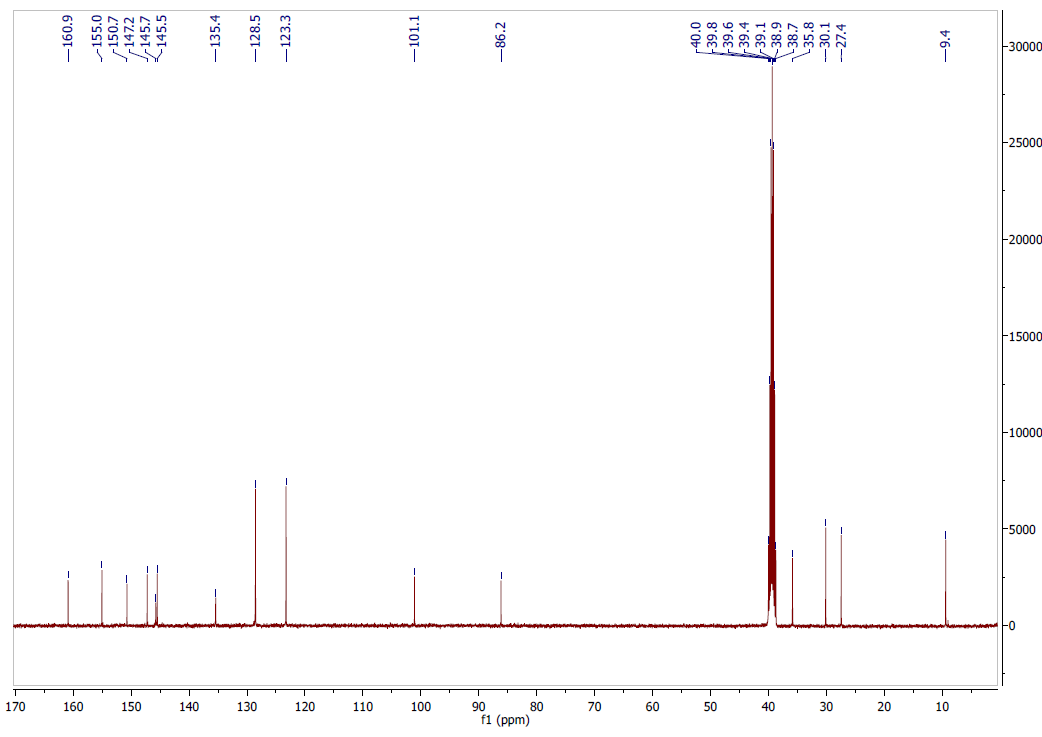
*

^13^C-NMR Spectrum of 3-methyl-4-(3-nitrophenyl)-1-phenyl-1,8-dihydro-5H-pyrazolo[4',3':5,6]pyrido[2,3-d]pyrimidine-5,7(6H)-dione

*
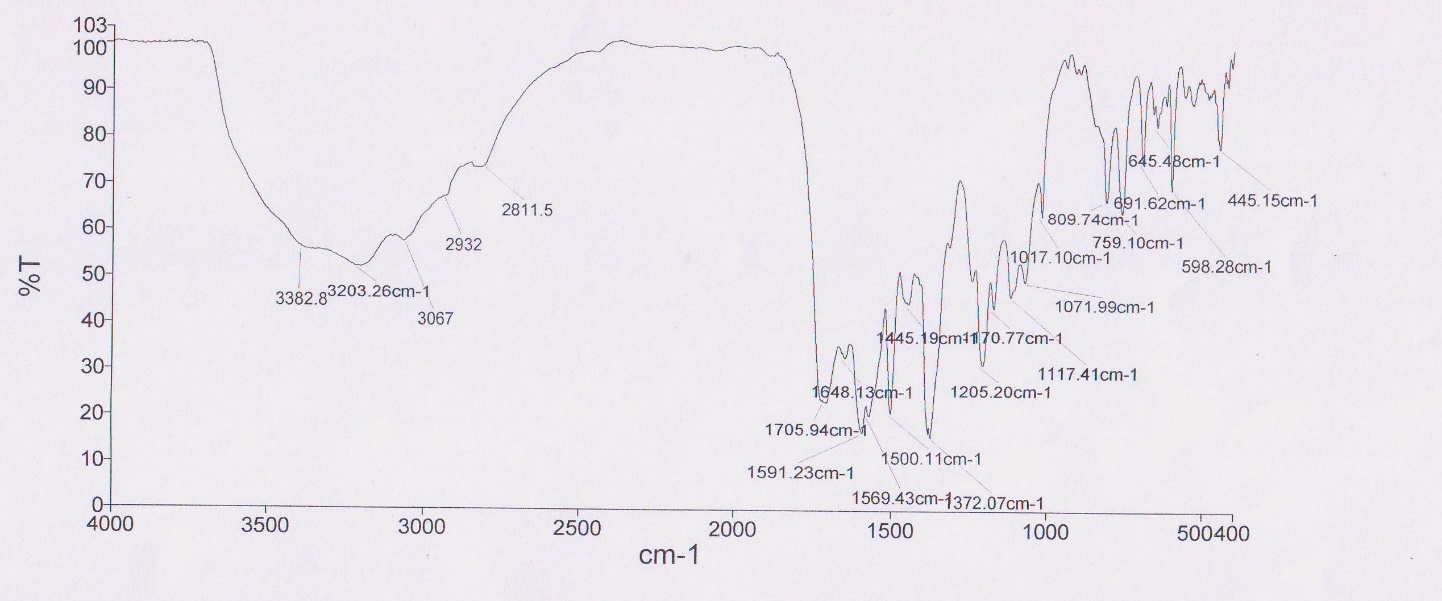
*

FT-IR Spectrum of 4-(2-hydroxy-3-methoxyphenyl)-3-methyl-1-phenyl-1,8-dihydro-5H-pyrazolo[4',3':5,6]pyrido[2,3-d]pyrimidine-5,7(6H)-dione

*
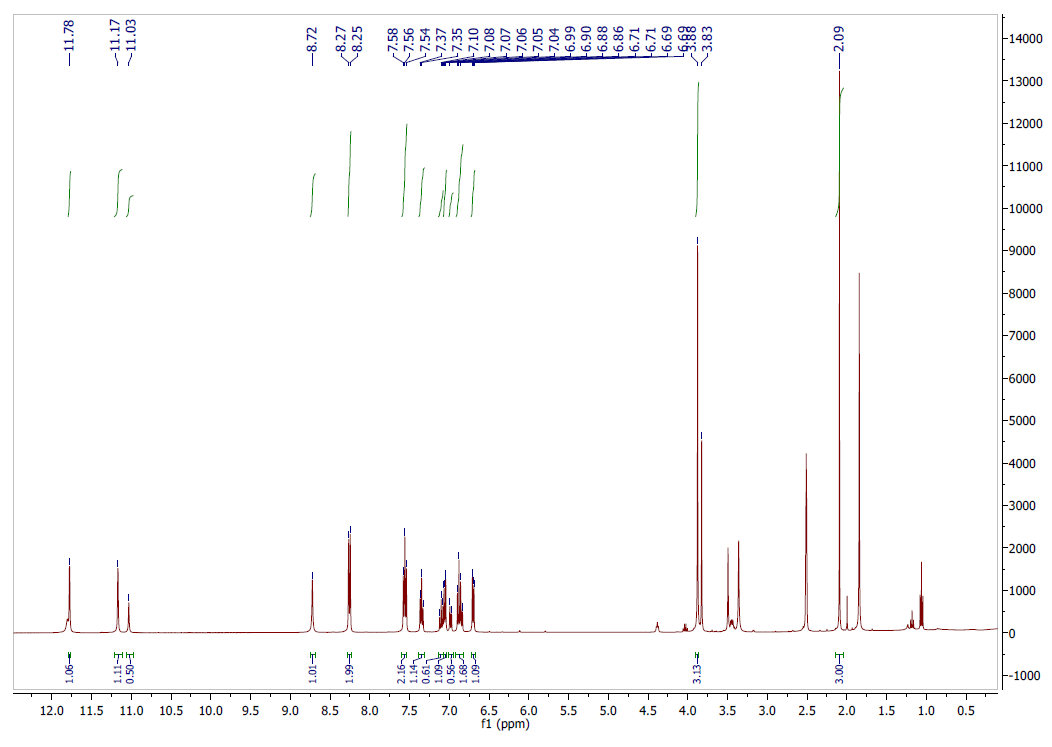
*

^1^H-NMR Spectrum of 4-(2-hydroxy-3-methoxyphenyl)-3-methyl-1-phenyl-1,8-dihydro-5H-pyrazolo[4',3':5,6]pyrido[2,3-d]pyrimidine-5,7(6H)-dione

*
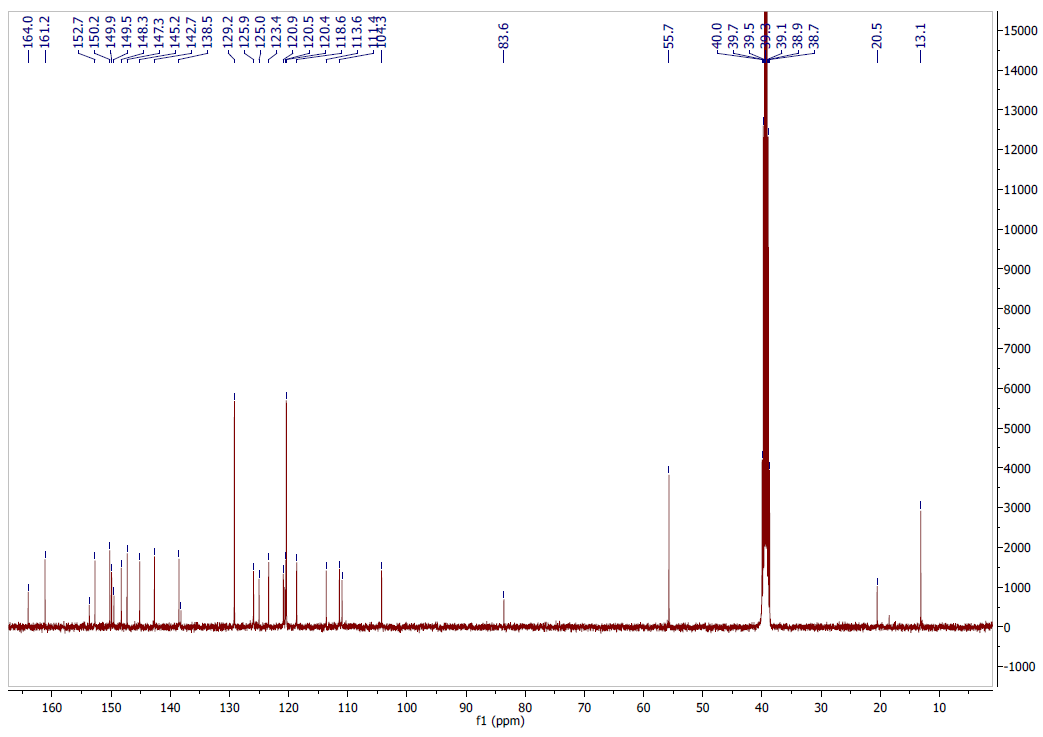
*

^13^C-NMR Spectrum of 4-(2-hydroxy-3-methoxyphenyl)-3-methyl-1-phenyl-1,8-dihydro-5H-pyrazolo[4',3':5,6]pyrido[2,3-d]pyrimidine-5,7(6H)-dione

*
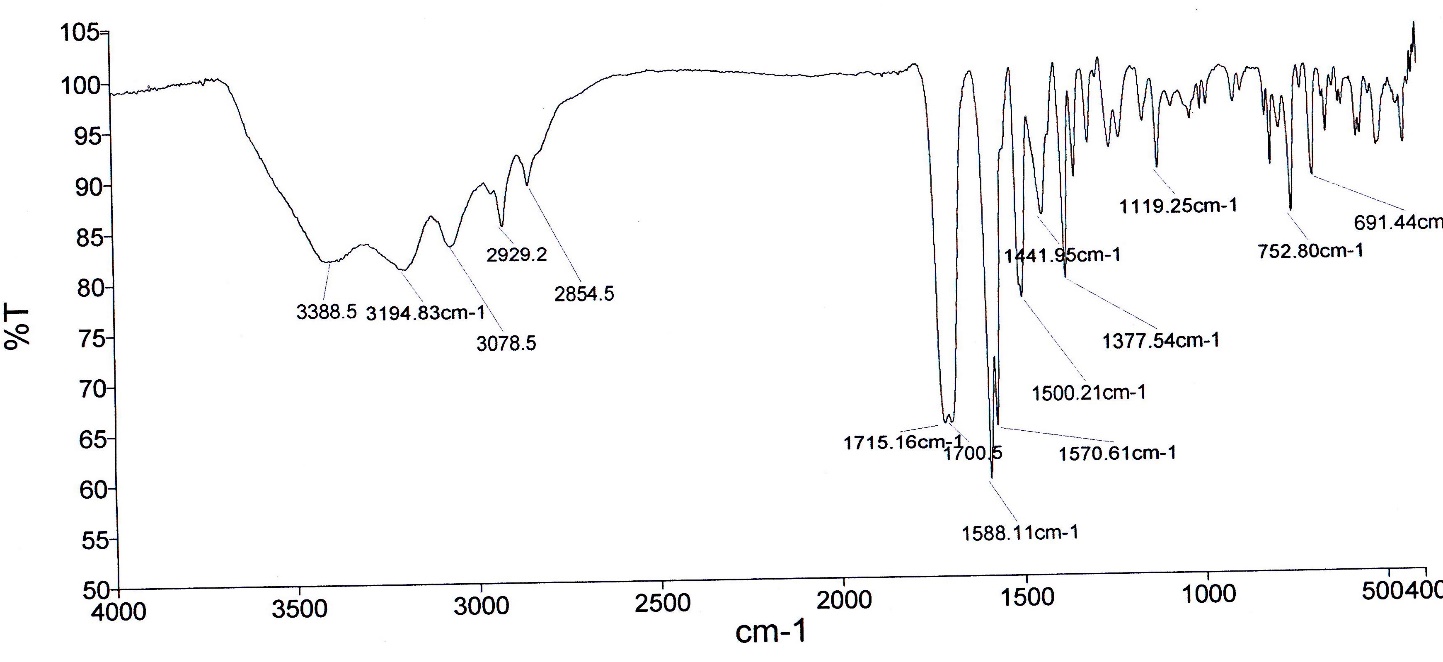
*

FT-IR Spectrum of 4-(3-hydroxyphenyl)-3-methyl-1-phenyl-1,8-dihydro-5H-pyrazolo[4',3':5,6]pyrido[2,3-d]pyrimidine-5,7(6H)-dione

*
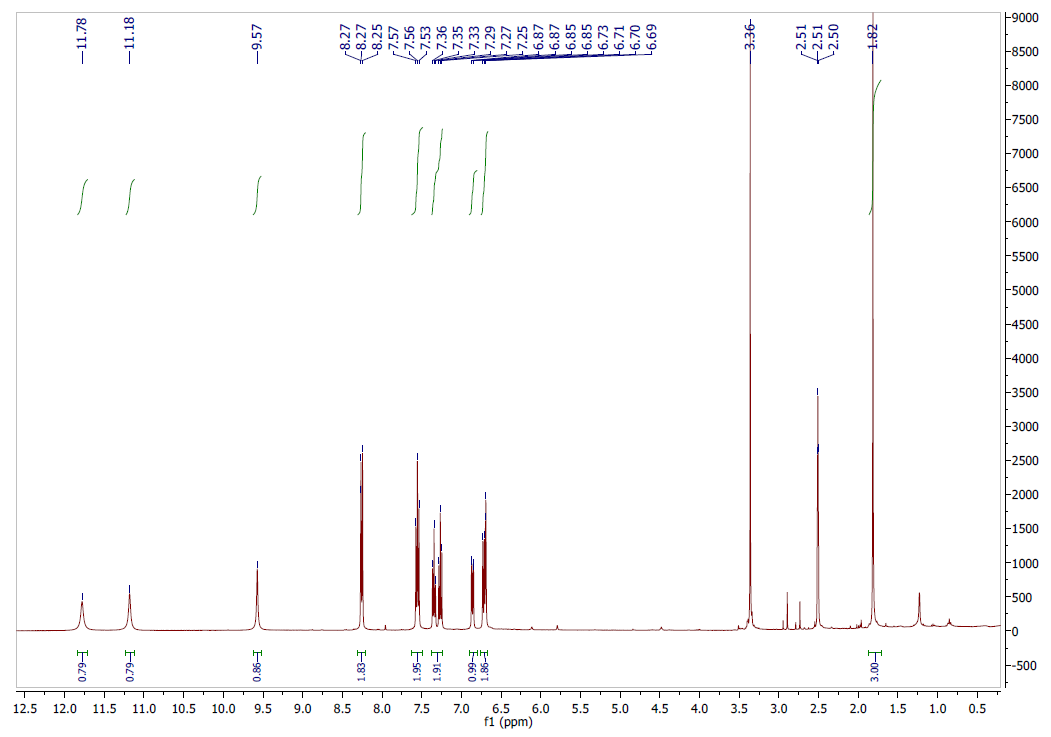
*

^1^H-NMR Spectrum of 4-(3-hydroxyphenyl)-3-methyl-1-phenyl-1,8-dihydro-5H-pyrazolo[4',3':5,6]pyrido[2,3-d]pyrimidine-5,7(6H)-dione

*
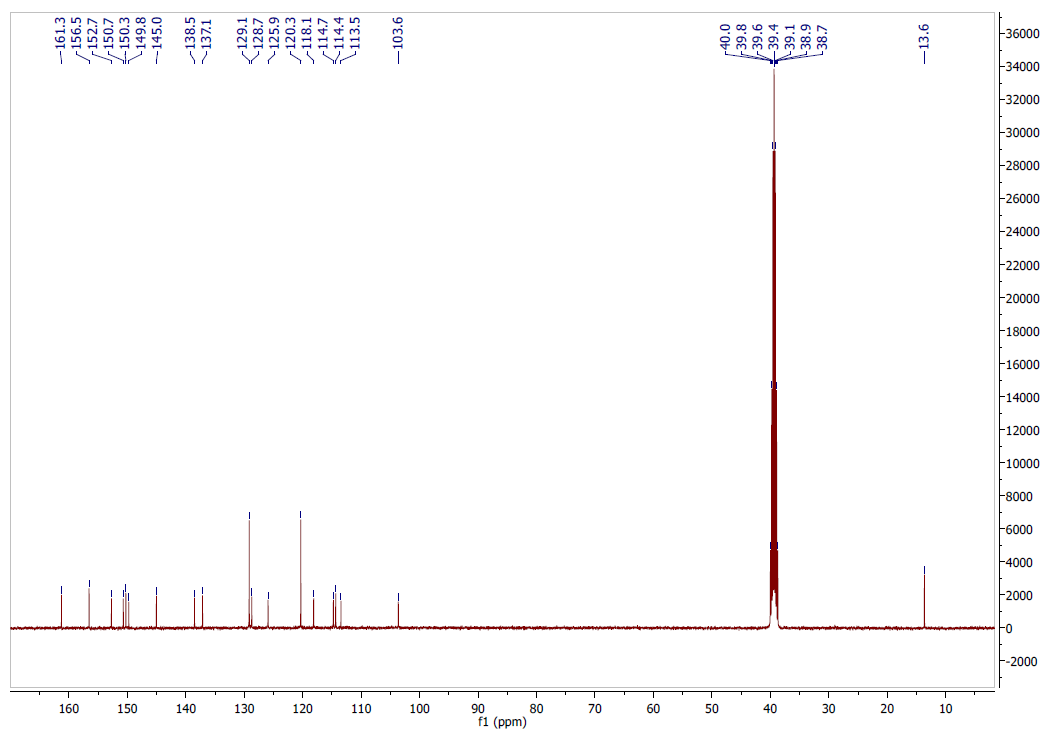
*

^13^C-NMR Spectrum of 4-(3-hydroxyphenyl)-3-methyl-1-phenyl-1,8-dihydro-5H-pyrazolo[4',3':5,6]pyrido[2,3-d]pyrimidine-5,7(6H)-dione

*
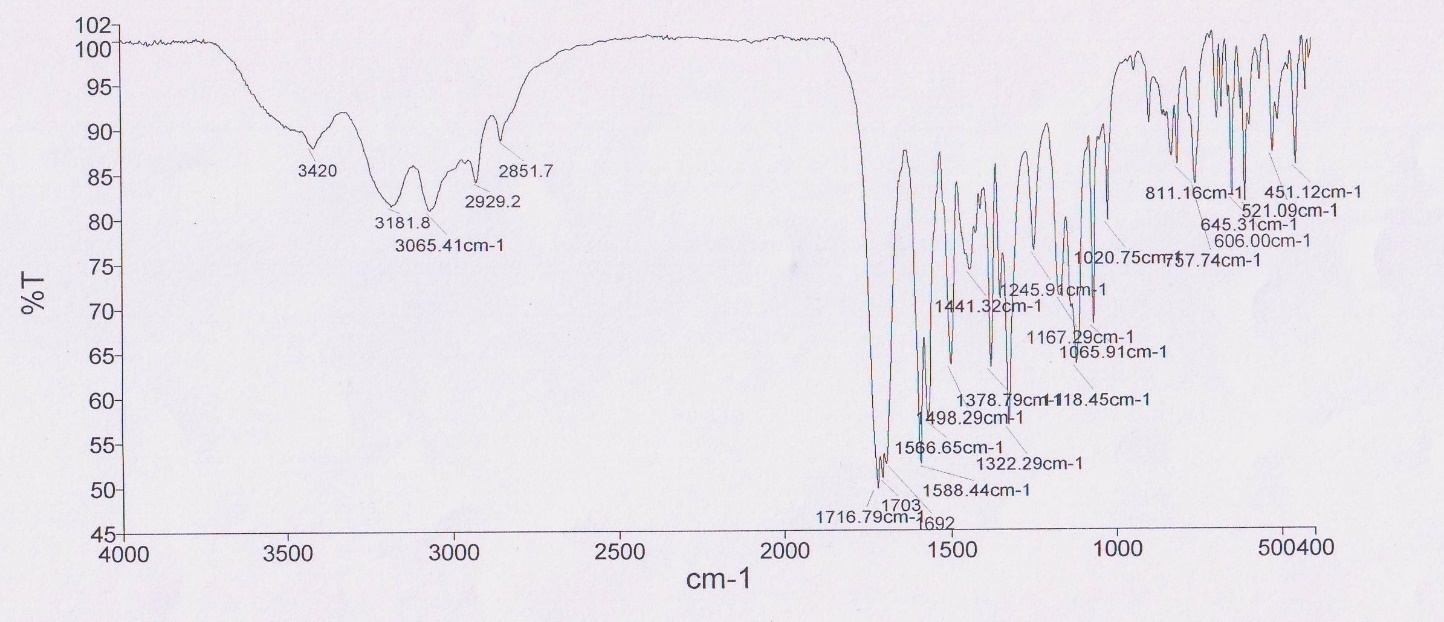
*

FT-IR Spectrum of 3-methyl-1-phenyl-4-(4-(trifluoromethyl)phenyl)-1,8-dihydro-5H-pyrazolo[4',3':5,6]pyrido[2,3-d]pyrimidine-5,7(6H)-dione

*
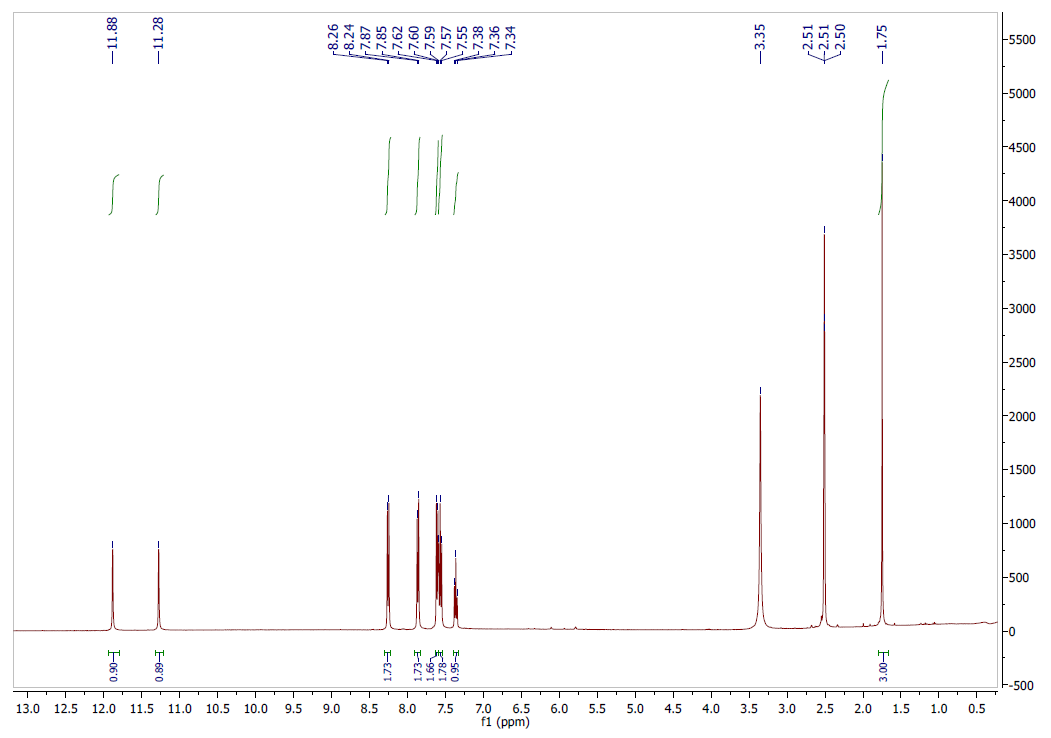
*

^1^H-NMR Spectrum of 3-methyl-1-phenyl-4-(4-(trifluoromethyl)phenyl)-1,8-dihydro-5H-pyrazolo[4',3':5,6]pyrido[2,3-d]pyrimidine-5,7(6H)-dione

*
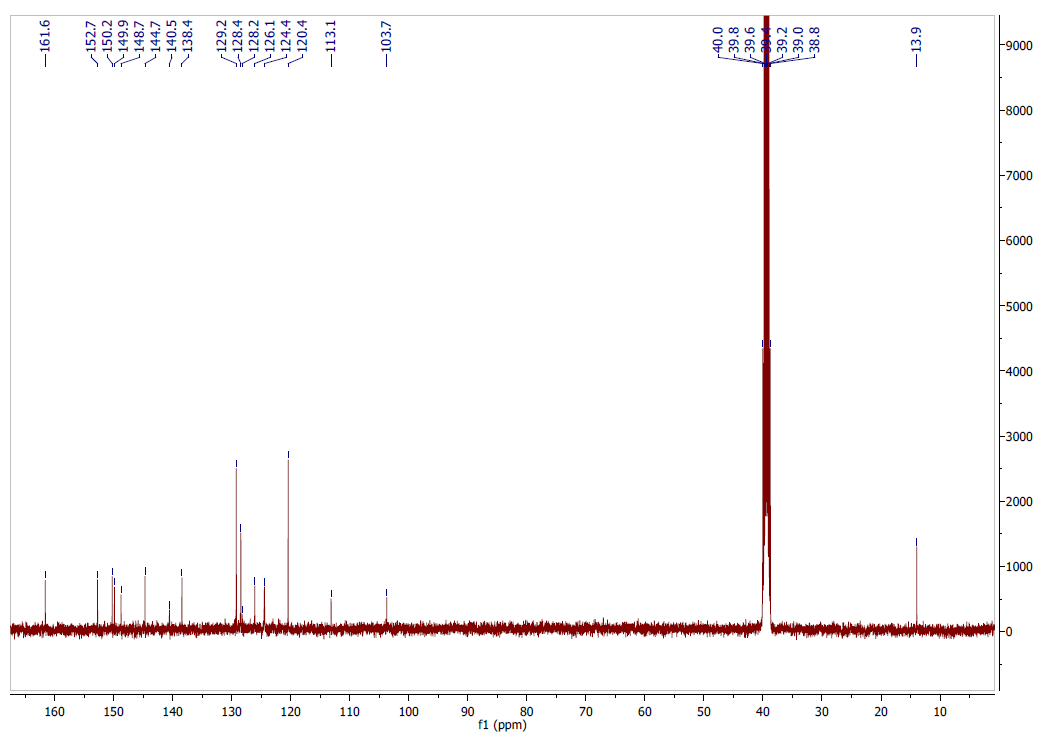
*

^13^C-NMR Spectrum of 3-methyl-1-phenyl-4-(4-(trifluoromethyl)phenyl)-1,8-dihydro-5H-pyrazolo[4',3':5,6]pyrido[2,3-d]pyrimidine-5,7(6H)-dione

*
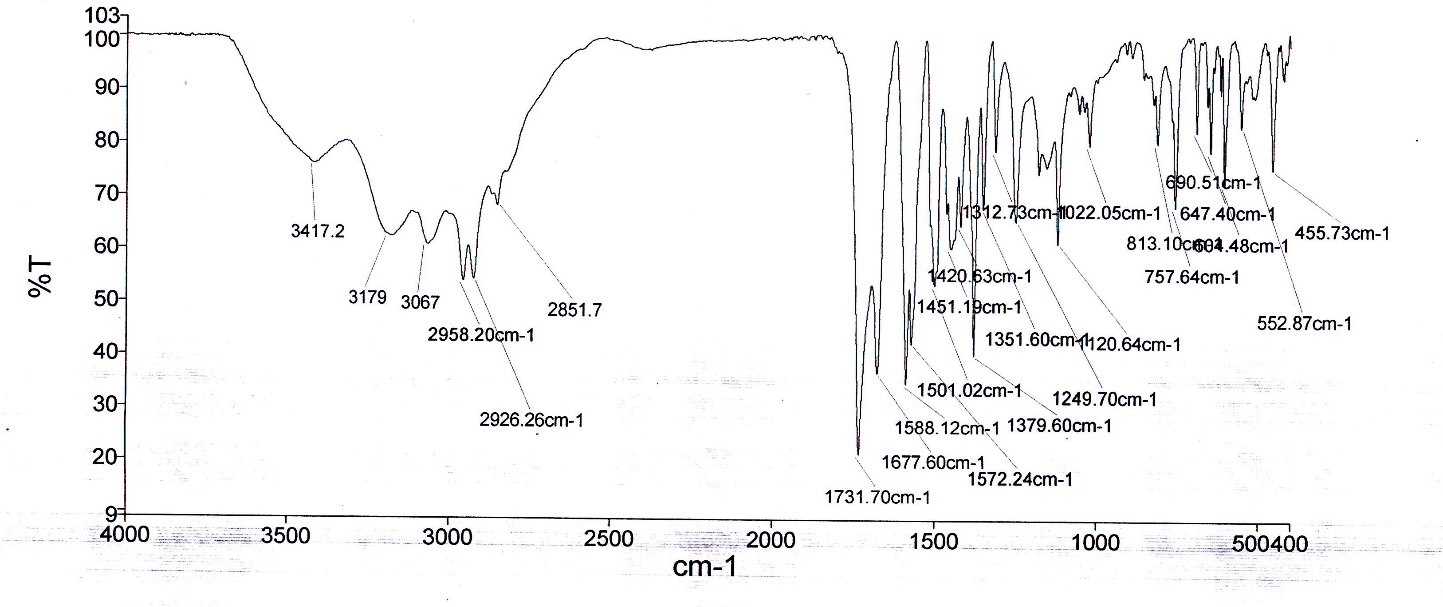
*

FT-IR Spectrum of 4-(4-isopropylphenyl)-3-methyl-1-phenyl-1,8-dihydro-5H-pyrazolo[4',3':5,6]pyrido[2,3-d]pyrimidine-5,7(6H)-dione

*
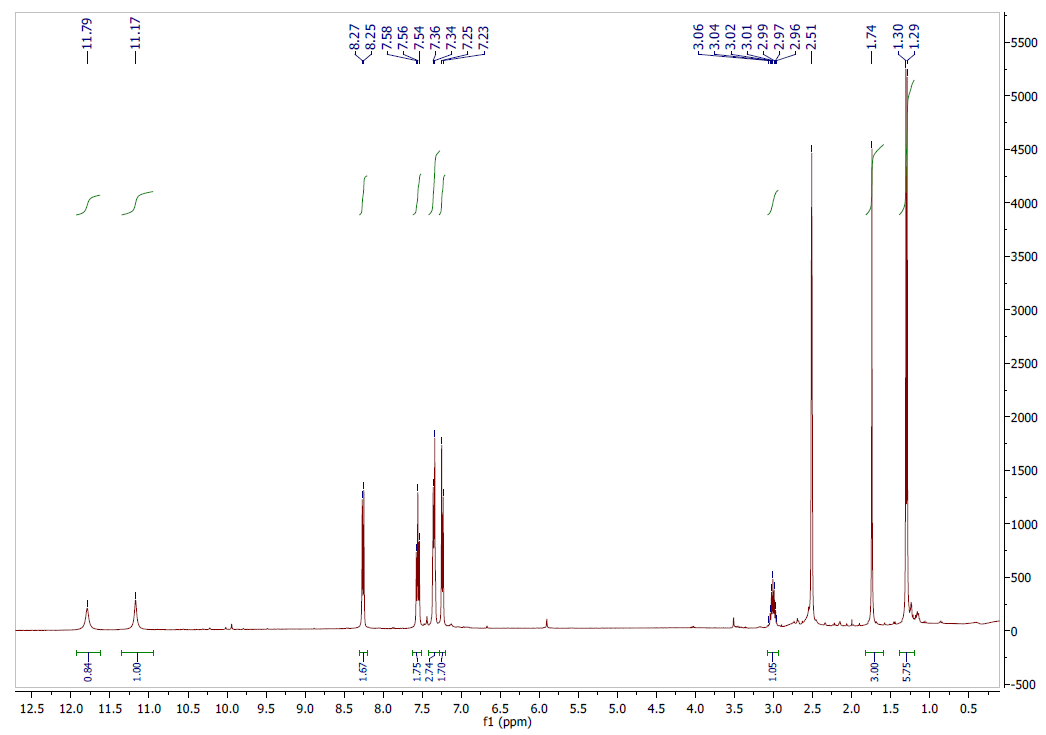
*

^1^H-NMR Spectrum of 4-(4-isopropylphenyl)-3-methyl-1-phenyl-1,8-dihydro-5H-pyrazolo[4',3':5,6]pyrido[2,3-d]pyrimidine-5,7(6H)-dione

*
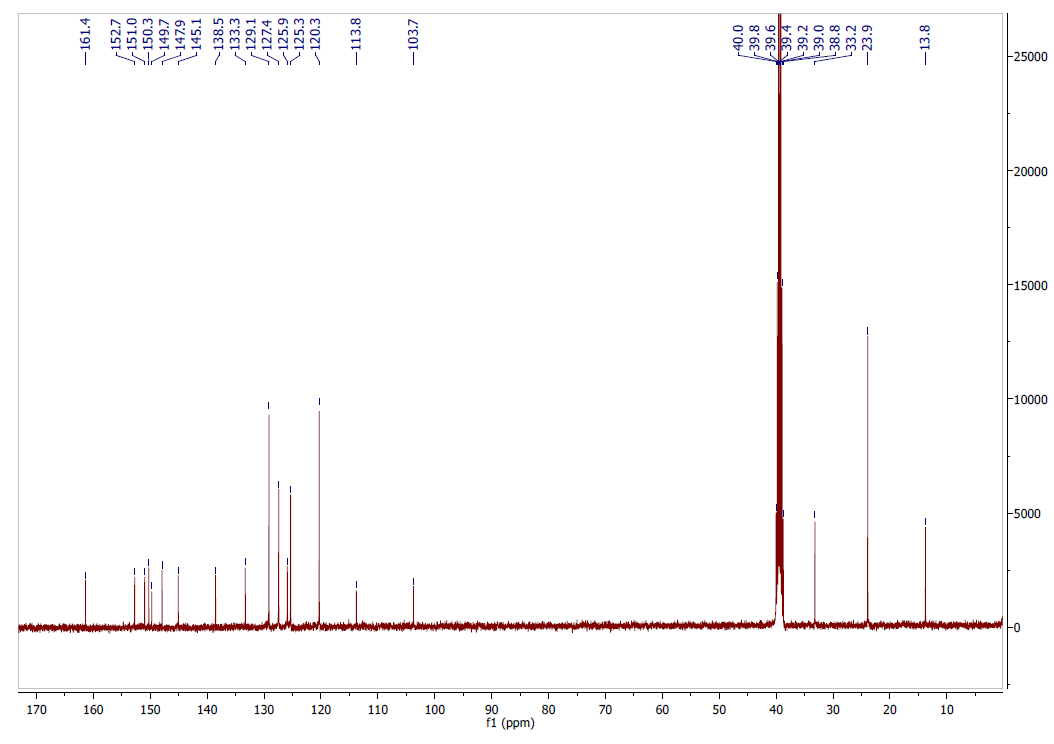
*

^13^C-NMR Spectrum of 4-(4-isopropylphenyl)-3-methyl-1-phenyl-1,8-dihydro-5H-pyrazolo[4',3':5,6]pyrido[2,3-d]pyrimidine-5,7(6H)-dione

*
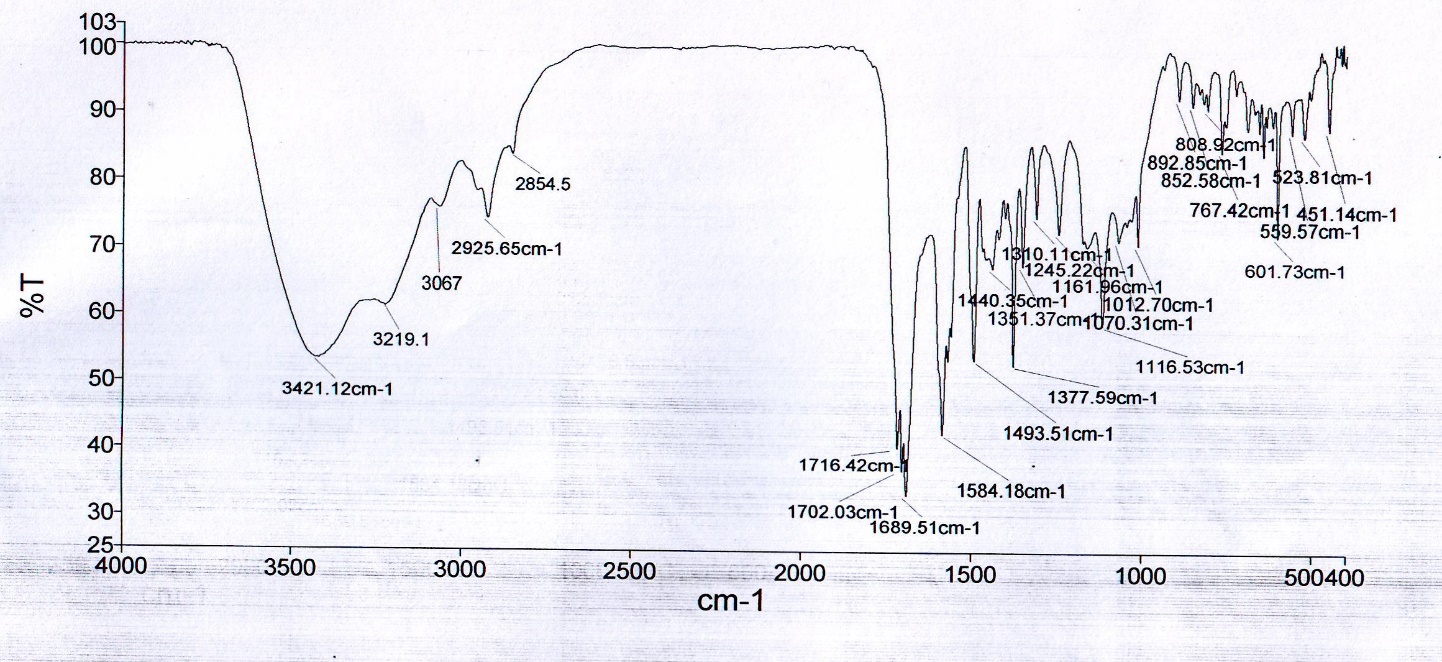
*

FT-IR Spectrum of 4-(4-bromophenyl)-3-methyl-1-phenyl-1,8-dihydro-5H-pyrazolo[4',3':5,6]pyrido[2,3-d]pyrimidine-5,7(6H)-dione

*
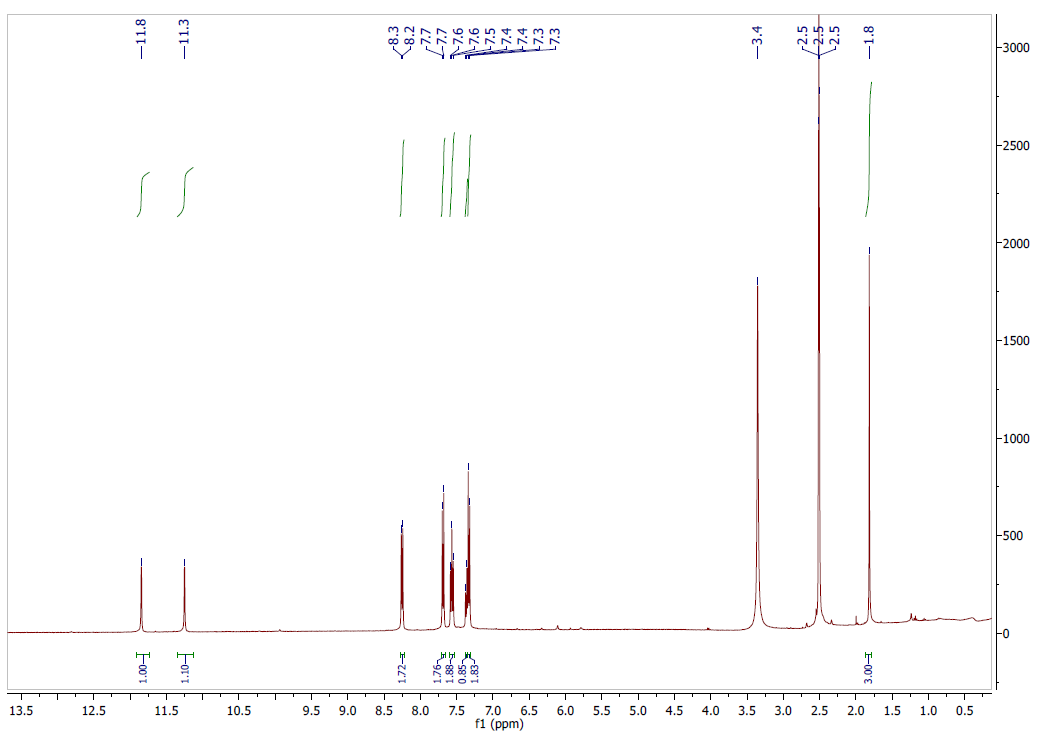
*

^1^H-NMR Spectrum of 4-(4-bromophenyl)-3-methyl-1-phenyl-1,8-dihydro-5H-pyrazolo[4',3':5,6]pyrido[2,3-d]pyrimidine-5,7(6H)-dione

*
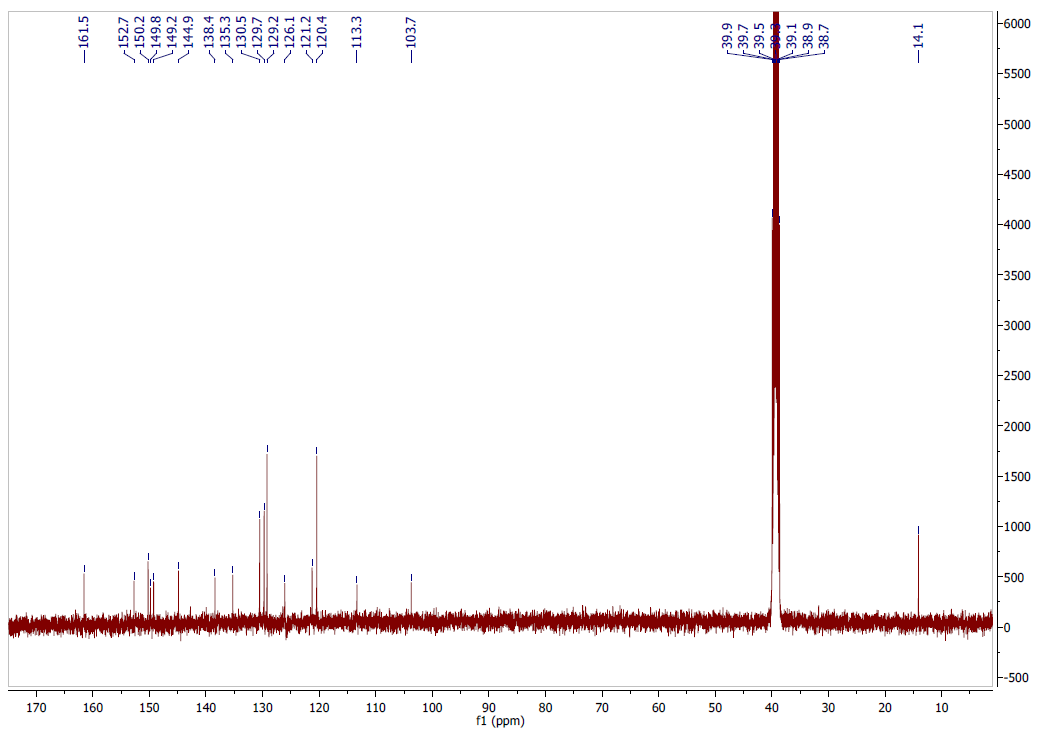
*

^13^C-NMR Spectrum of 4-(4-bromophenyl)-3-methyl-1-phenyl-1,8-dihydro-5H-pyrazolo[4',3':5,6]pyrido[2,3-d]pyrimidine-5,7(6H)-dione

*
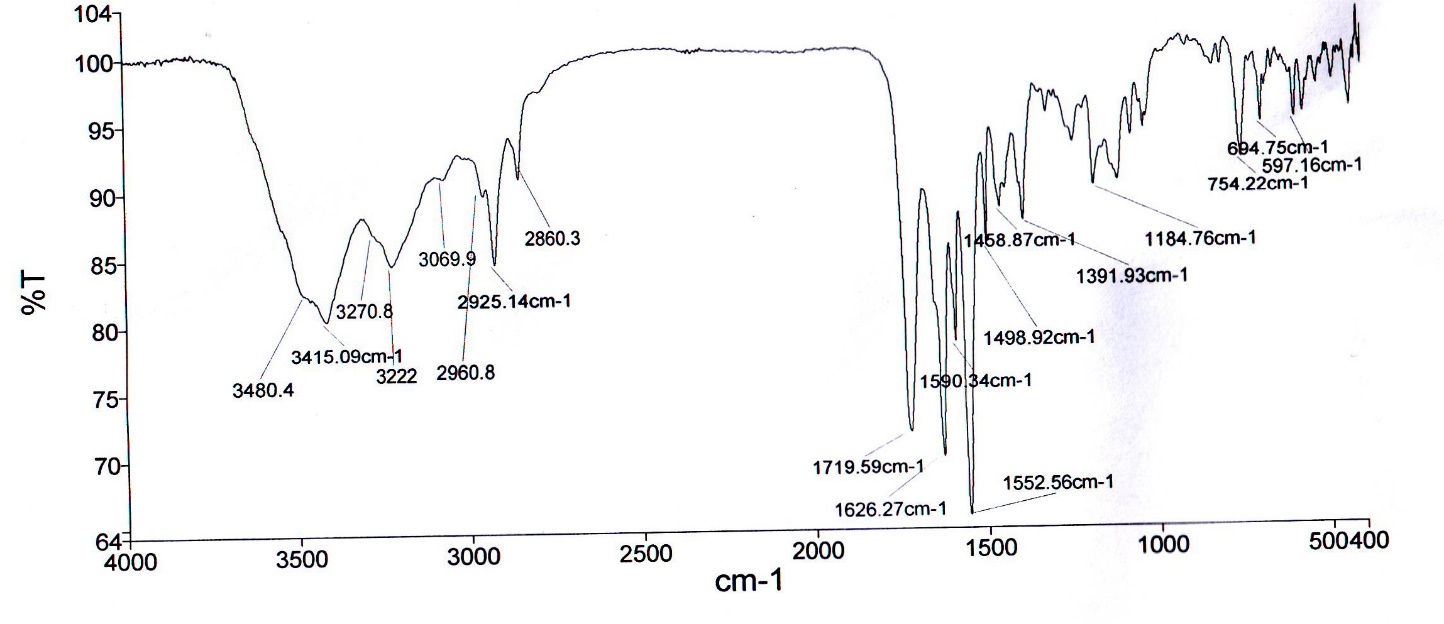
*

FT-IR Spectrum of 4-(4-chlorophenyl)-3-methyl-1-phenyl-1,8-dihydro-5H-pyrazolo[4',3':5,6]pyrido[2,3-d]pyrimidine-5,7(6H)-dione

*
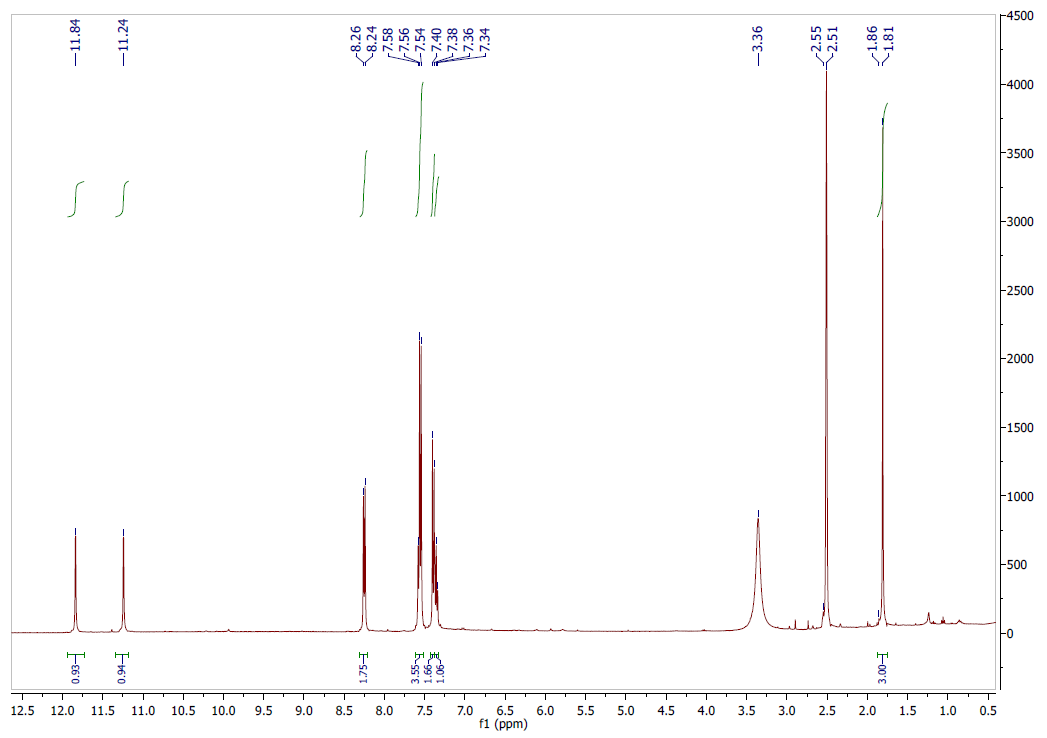
*

^1^H-NMR Spectrum of 4-(4-chlorophenyl)-3-methyl-1-phenyl-1,8-dihydro-5H-pyrazolo[4',3':5,6]pyrido[2,3-d]pyrimidine-5,7(6H)-dione

*
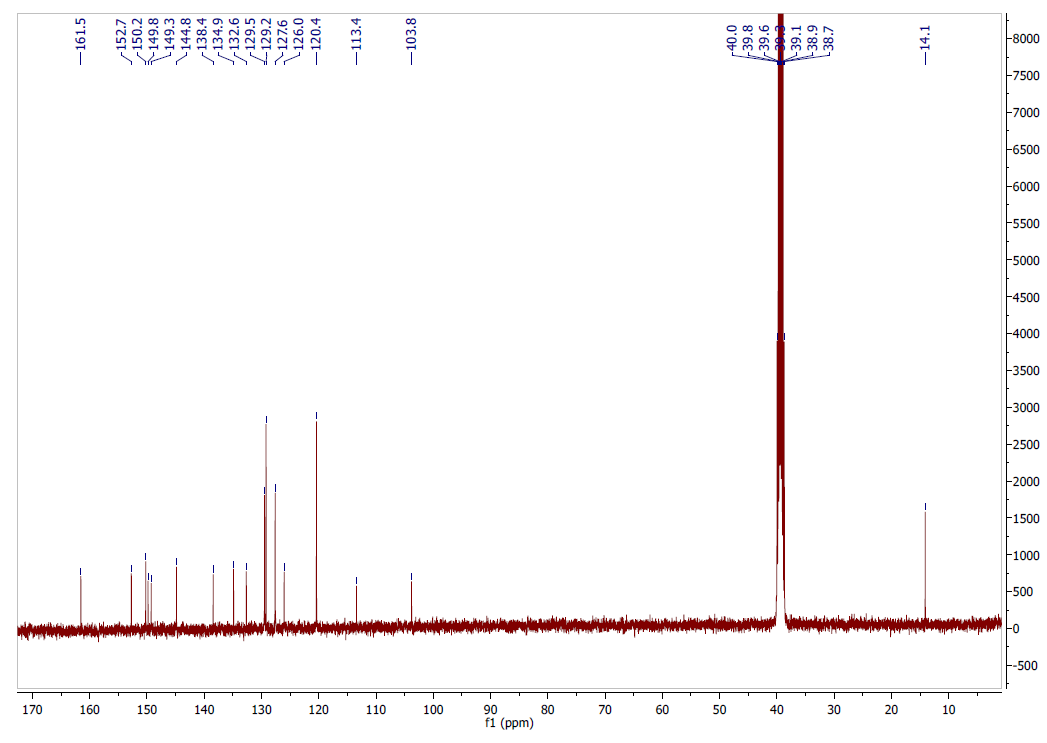
*

^13^C-NMR Spectrum of 4-(4-chlorophenyl)-3-methyl-1-phenyl-1,8-dihydro-5H-pyrazolo[4',3':5,6]pyrido[2,3-d]pyrimidine-5,7(6H)-dione

*
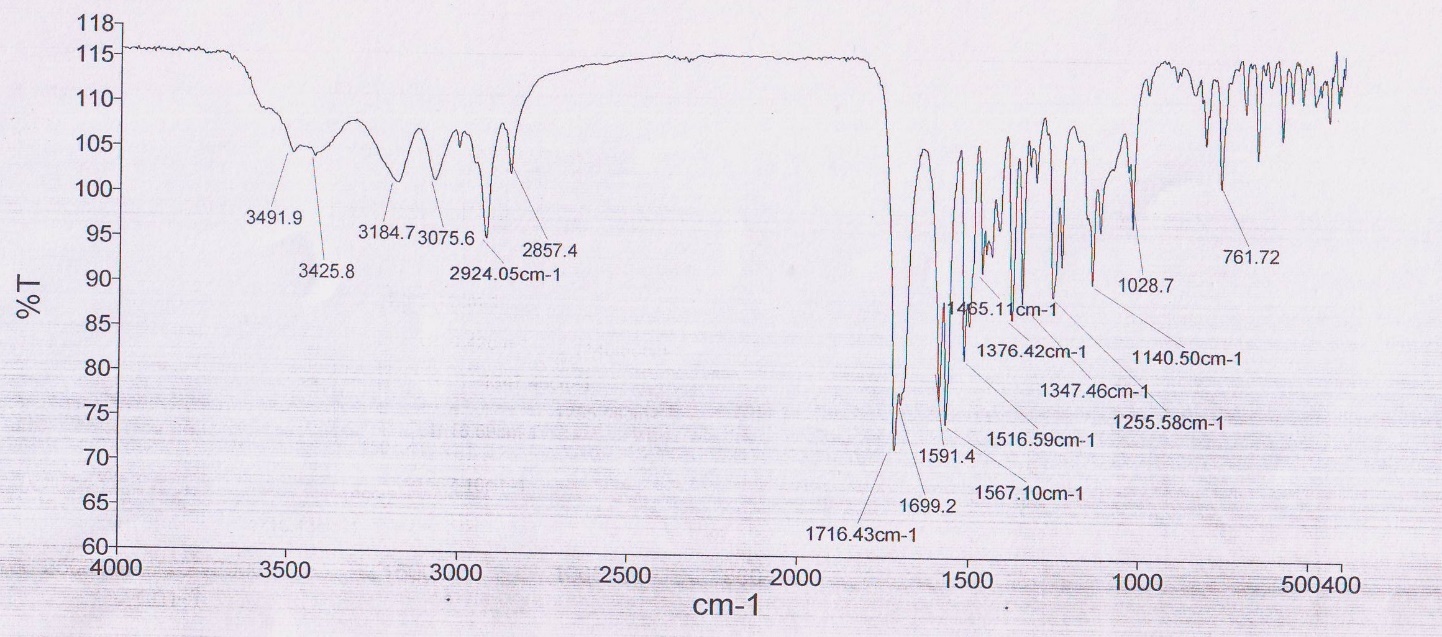
*

FT-IR Spectrum of 4-(3,4-dimethoxyphenyl)-3-methyl-1-phenyl-1,8-dihydro-5H-pyrazolo[4',3':5,6]pyrido[2,3-d]pyrimidine-5,7(6H)-dione

*
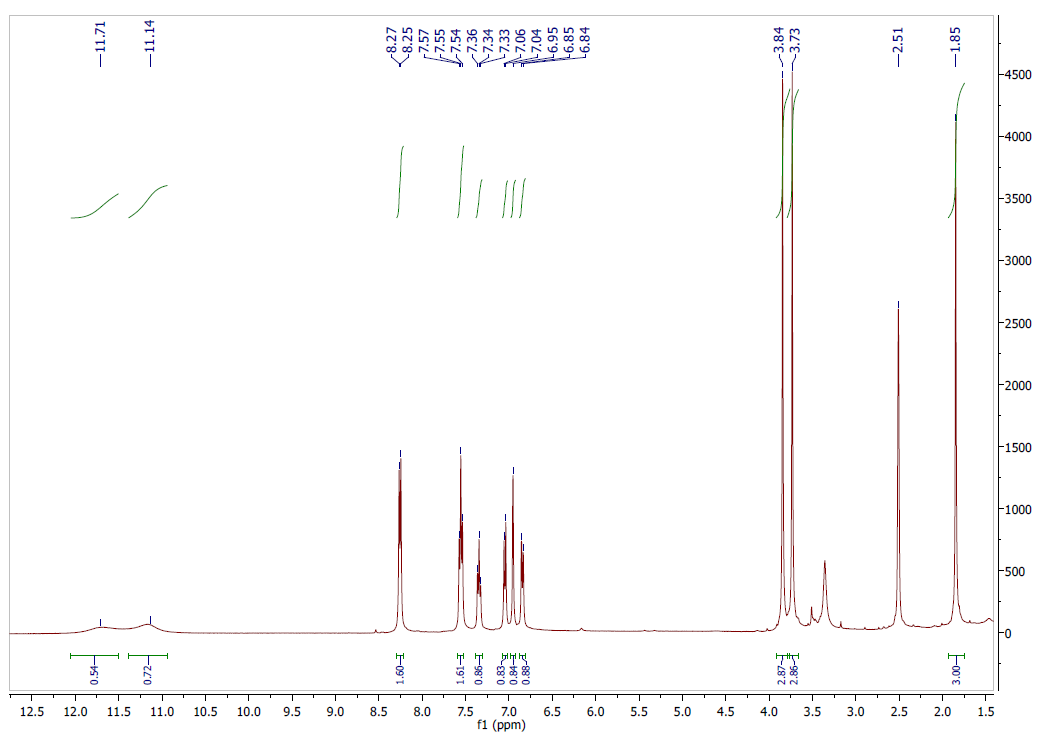
*

^1^H-NMR Spectrum of 4-(3,4-dimethoxyphenyl)-3-methyl-1-phenyl-1,8-dihydro-5H-pyrazolo[4',3':5,6]pyrido[2,3-d]pyrimidine-5,7(6H)-dione

*
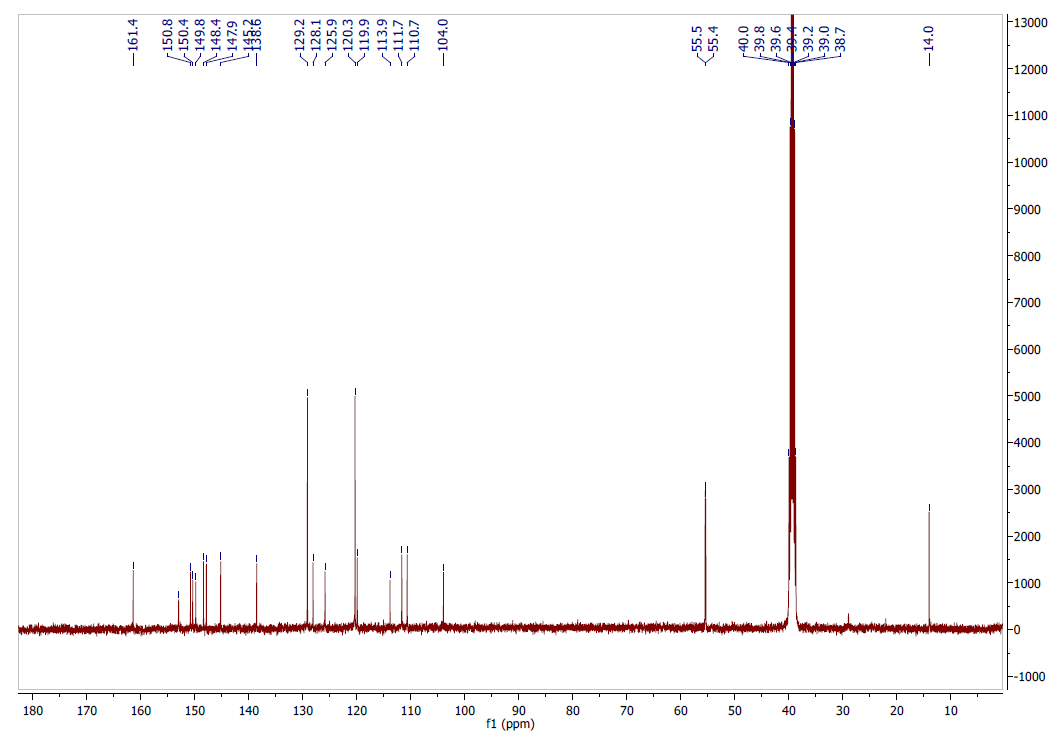
*

^13^C-NMR Spectrum of 4-(3,4-dimethoxyphenyl)-3-methyl-1-phenyl-1,8-dihydro-5H-pyrazolo[4',3':5,6]pyrido[2,3-d]pyrimidine-5,7(6H)-dione

*
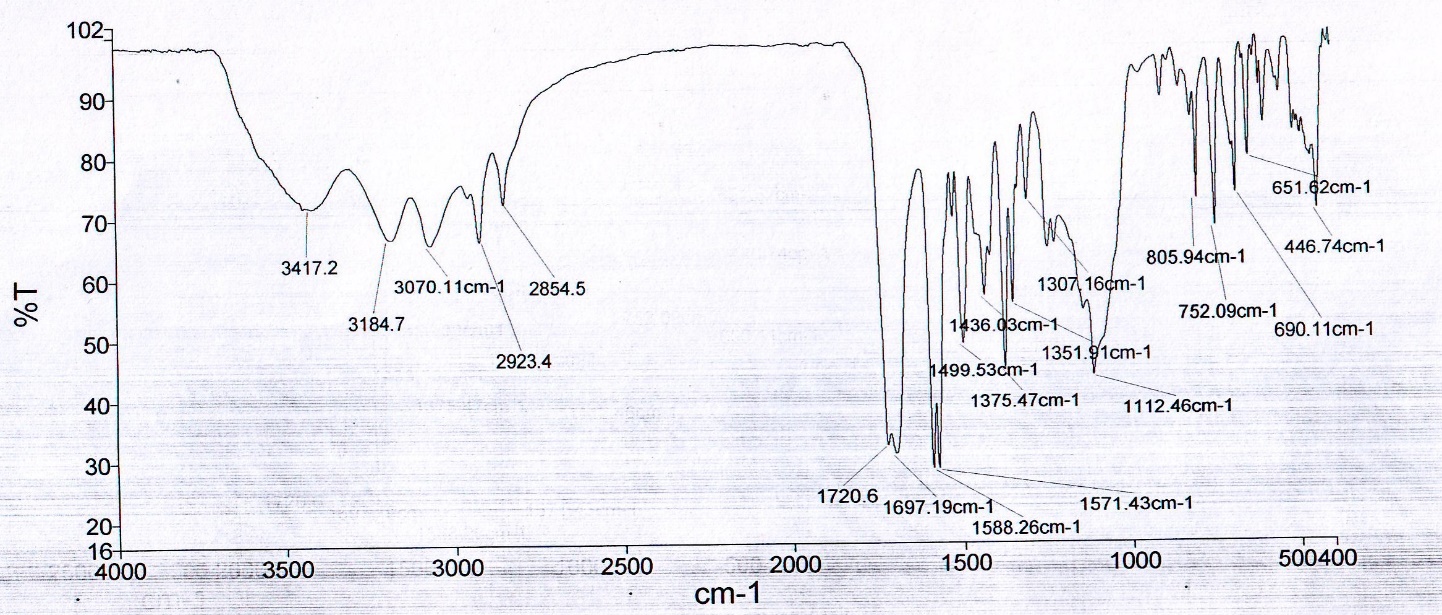
*

FT-IR Spectrum of 3-methyl-1-phenyl-4-(thiophen-2-yl)-1,8-dihydro-5H-pyrazolo[4',3':5,6]pyrido[2,3-d]pyrimidine-5,7(6H)-dione

*
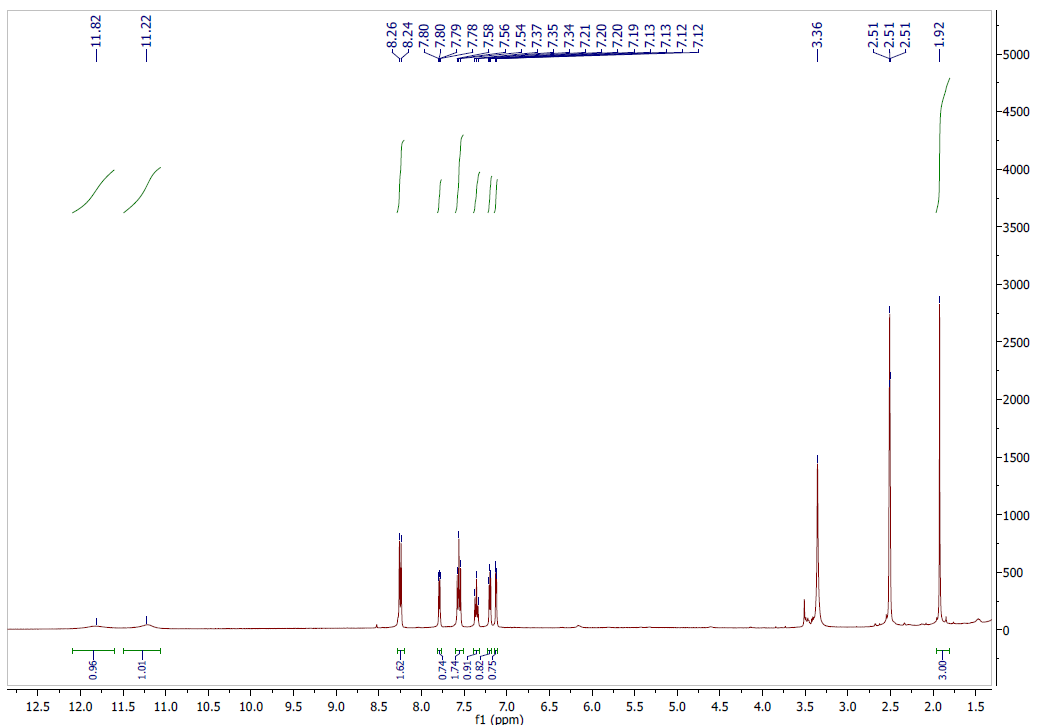
*

^1^H-NMR Spectrum of 3-methyl-1-phenyl-4-(thiophen-2-yl)-1,8-dihydro-5H-pyrazolo[4',3':5,6]pyrido[2,3-d]pyrimidine-5,7(6H)-dione

*
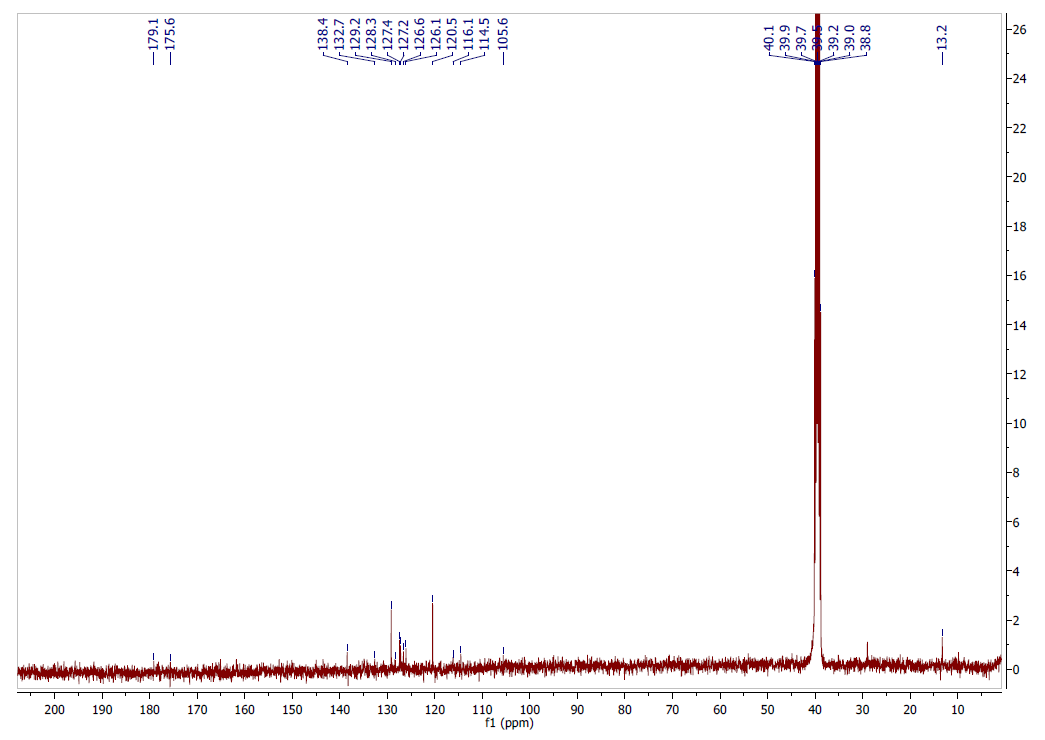
*

^13^C-NMR Spectrum of 3-methyl-1-phenyl-4-(thiophen-2-yl)-1,8-dihydro-5H-pyrazolo[4',3':5,6]pyrido[2,3-d]pyrimidine-5,7(6H)-dione

*
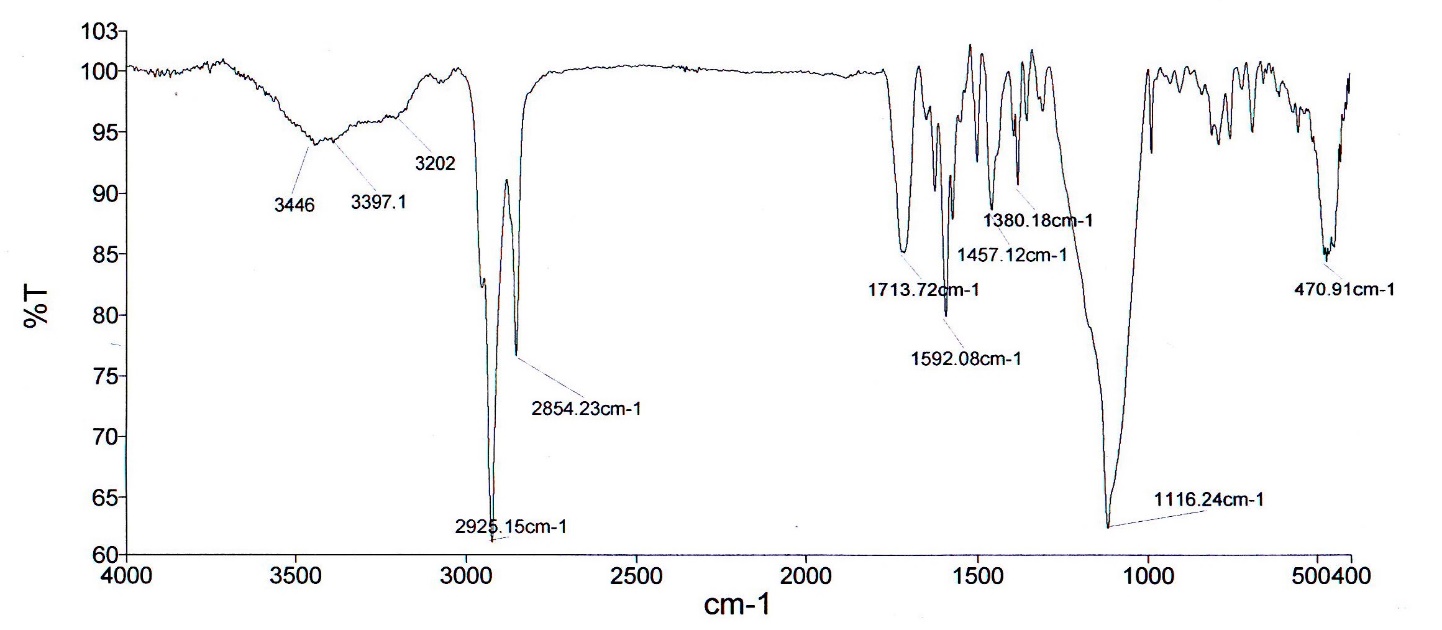
*

FT-IR Spectrum of 4-(3,5-difluorophenyl)-3-methyl-1-phenyl-1,8-dihydro-5H-pyrazolo[4',3':5,6]pyrido[2,3-d]pyrimidine-5,7(6H)-dione

*
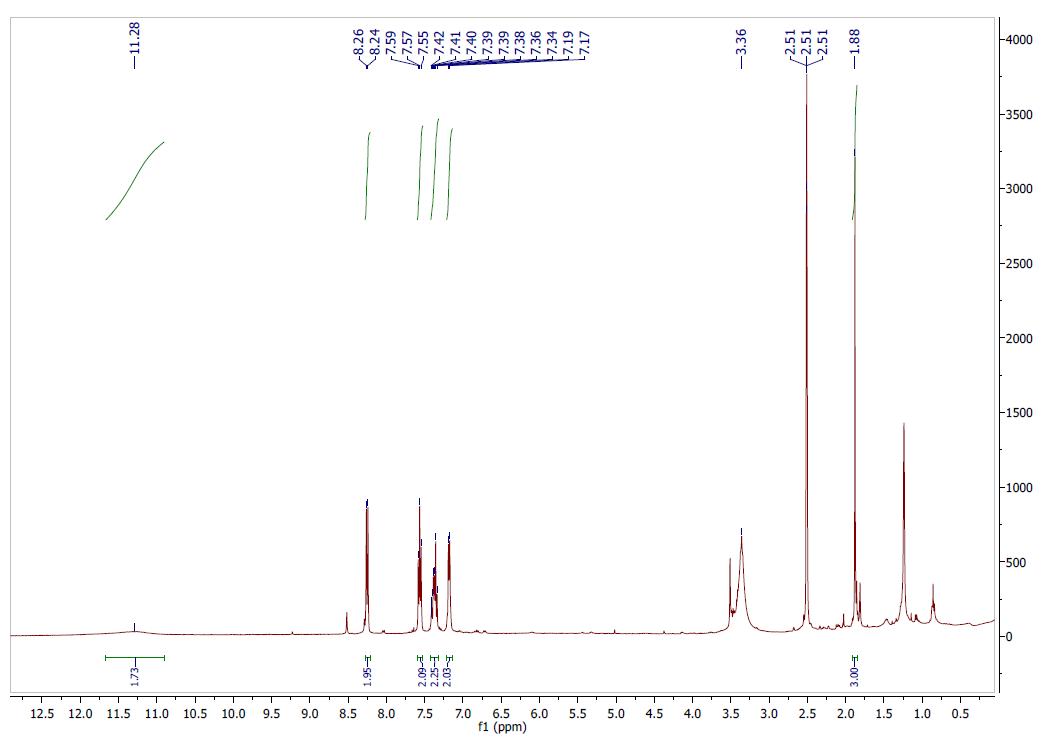
*

^1^H-NMR Spectrum of 4-(3,5-difluorophenyl)-3-methyl-1-phenyl-1,8-dihydro-5H-pyrazolo[4',3':5,6]pyrido[2,3-d]pyrimidine-5,7(6H)-dione

*
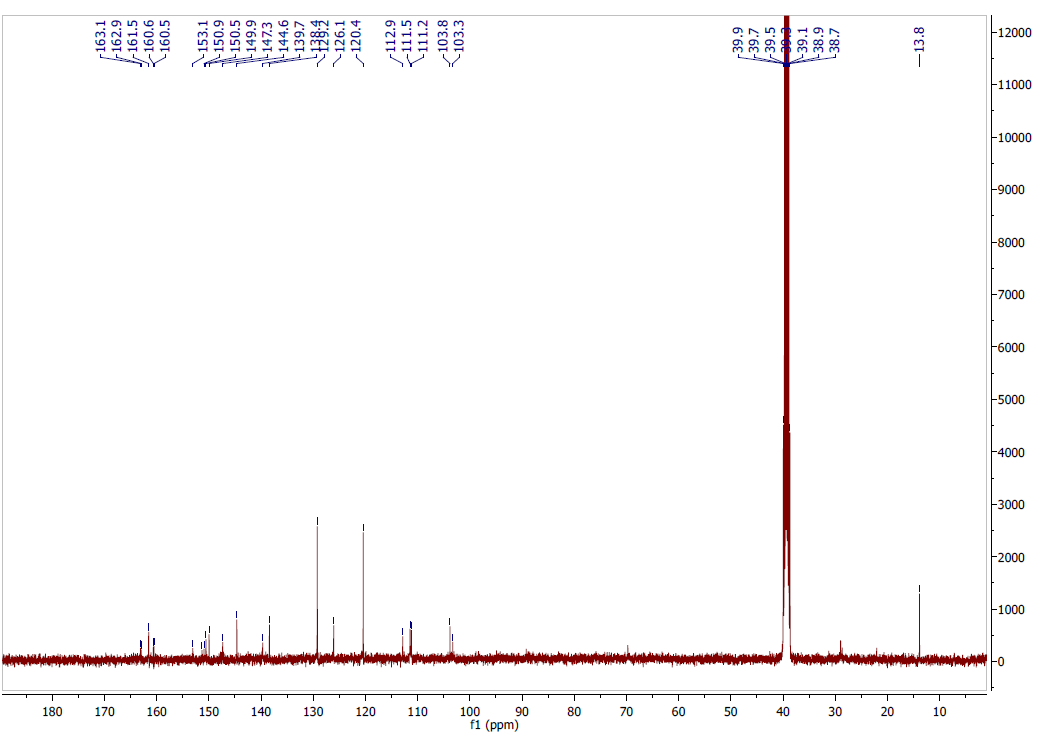
*

^13^C-NMR Spectrum of 4-(3,5-difluorophenyl)-3-methyl-1-phenyl-1,8-dihydro-5H-pyrazolo[4',3':5,6]pyrido[2,3-d]pyrimidine-5,7(6H)-dione

*
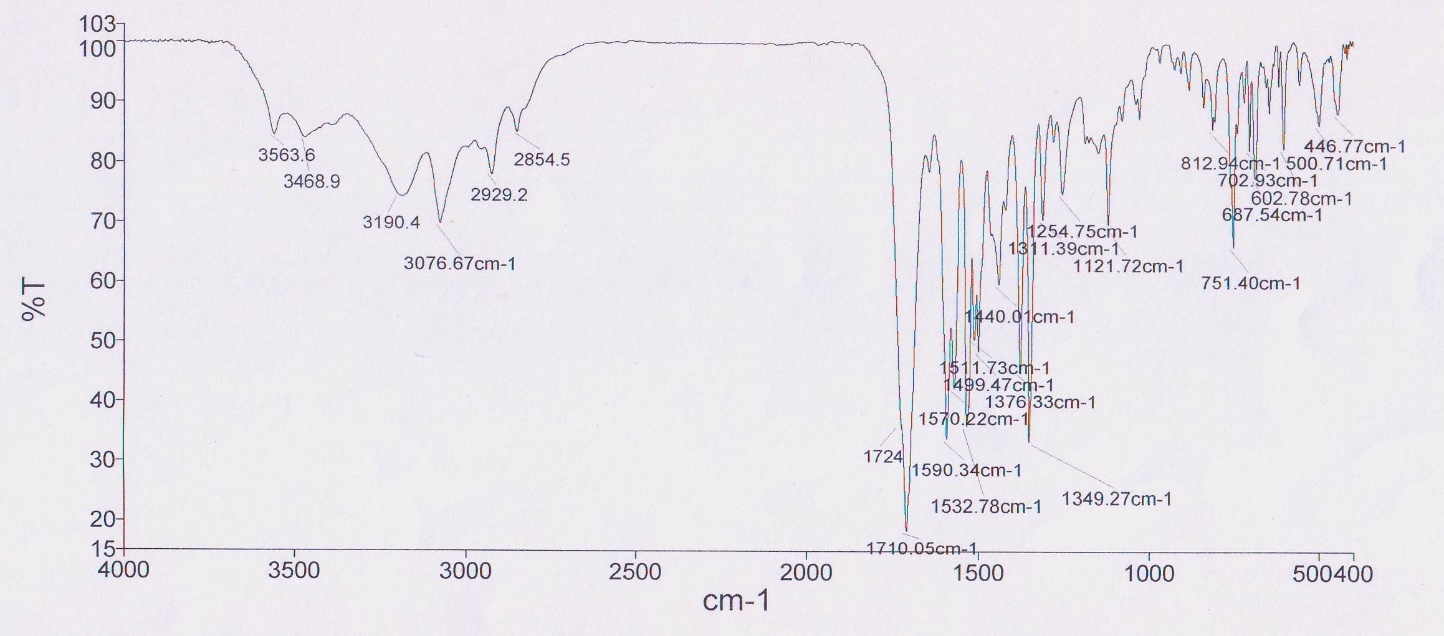
*

FT-IR Spectrum of 4-(3,4-difluorophenyl)-3-methyl-1-phenyl-1,8-dihydro-5H-pyrazolo[4',3':5,6]pyrido[2,3-d]pyrimidine-5,7(6H)-dione

*
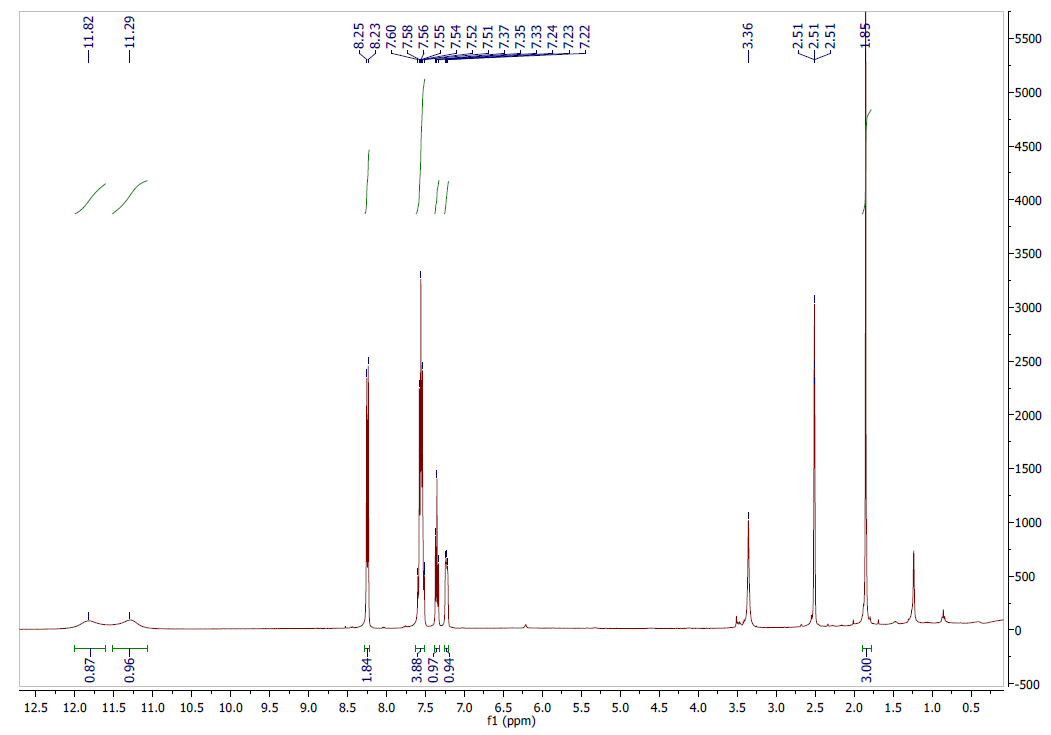
*

^1^H-NMR Spectrum of 4-(3,4-difluorophenyl)-3-methyl-1-phenyl-1,8-dihydro-5H-pyrazolo[4',3':5,6]pyrido[2,3-d]pyrimidine-5,7(6H)-dione

*
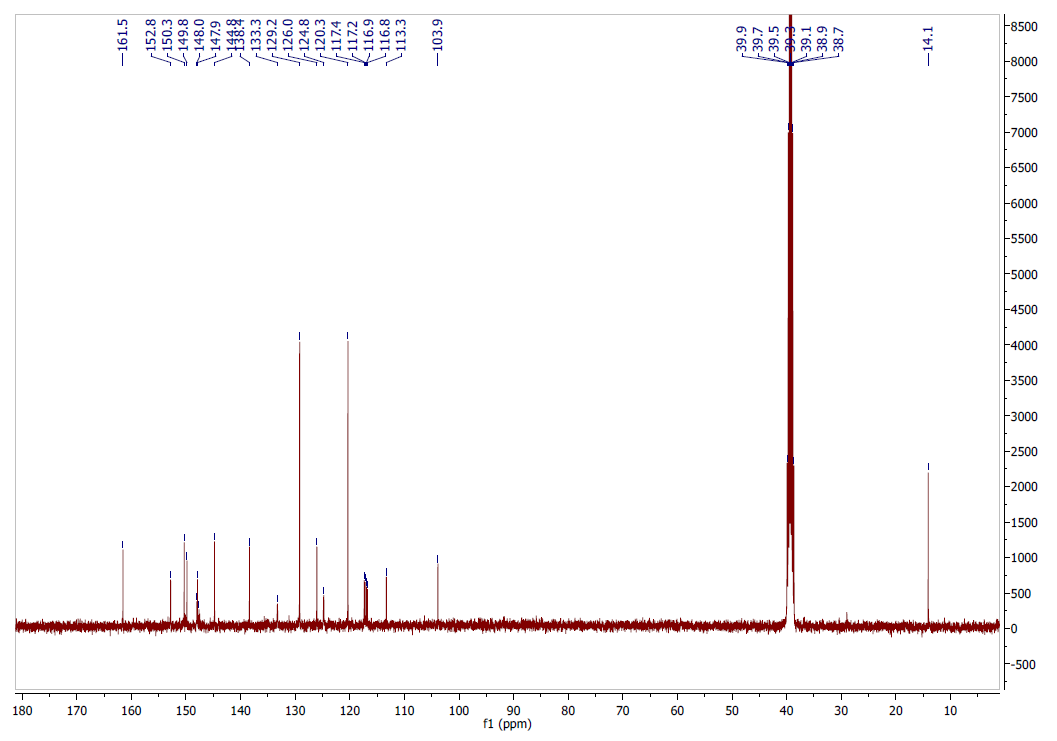
*

^13^C-NMR Spectrum of 4-(3,4-difluorophenyl)-3-methyl-1-phenyl-1,8-dihydro-5H-pyrazolo[4',3':5,6]pyrido[2,3-d]pyrimidine-5,7(6H)-dione

*L*

*
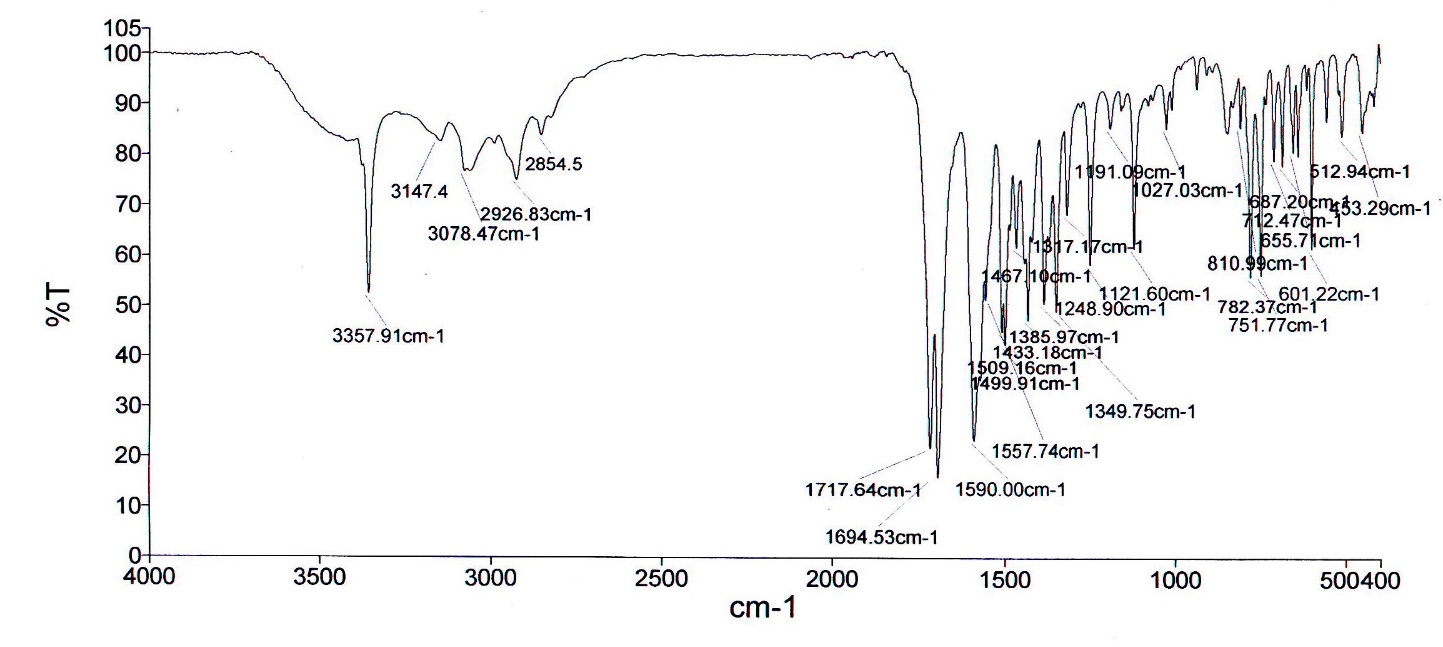
*

FT-IR Spectrum of 4-(2,6-dichlorophenyl)-3-methyl-1-phenyl-1,8-dihydro-5H-pyrazolo[4',3':5,6]pyrido[2,3-d]pyrimidine-5,7(6H)-dione

*
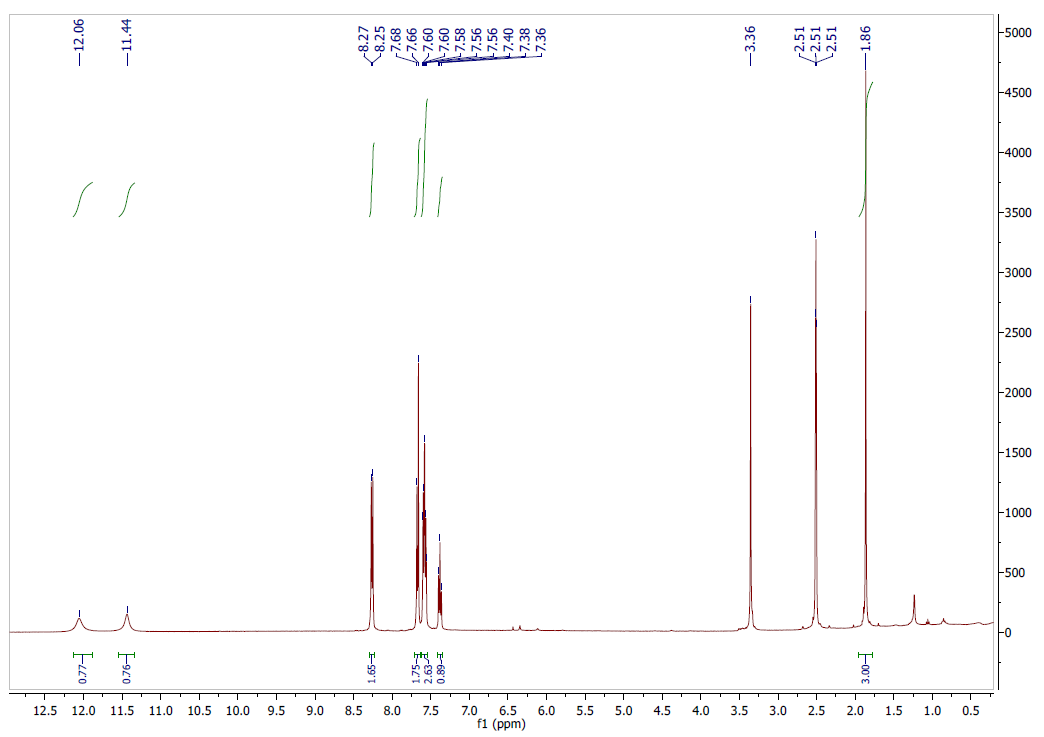
*

^1^H-NMR Spectrum of 4-(2,6-dichlorophenyl)-3-methyl-1-phenyl-1,8-dihydro-5H-pyrazolo[4',3':5,6]pyrido[2,3-d]pyrimidine-5,7(6H)-dione

*
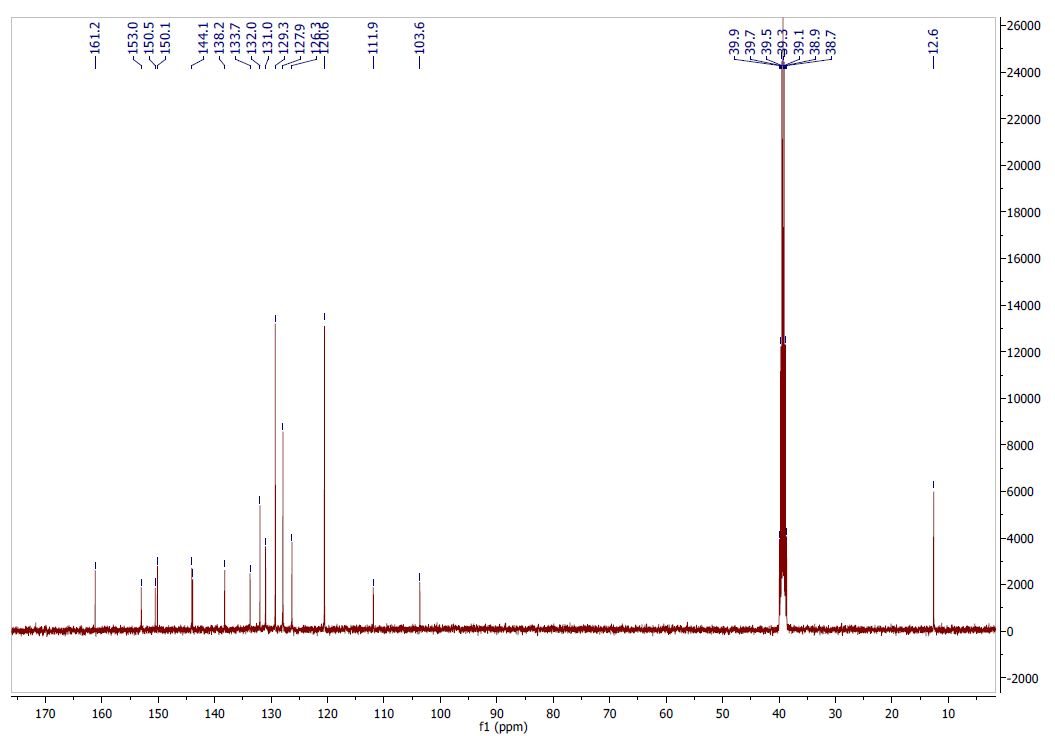
*

^13^C-NMR Spectrum of 4-(2,6-dichlorophenyl)-3-methyl-1-phenyl-1,8-dihydro-5H-pyrazolo[4',3':5,6]pyrido[2,3-d]pyrimidine-5,7(6H)-dione

*
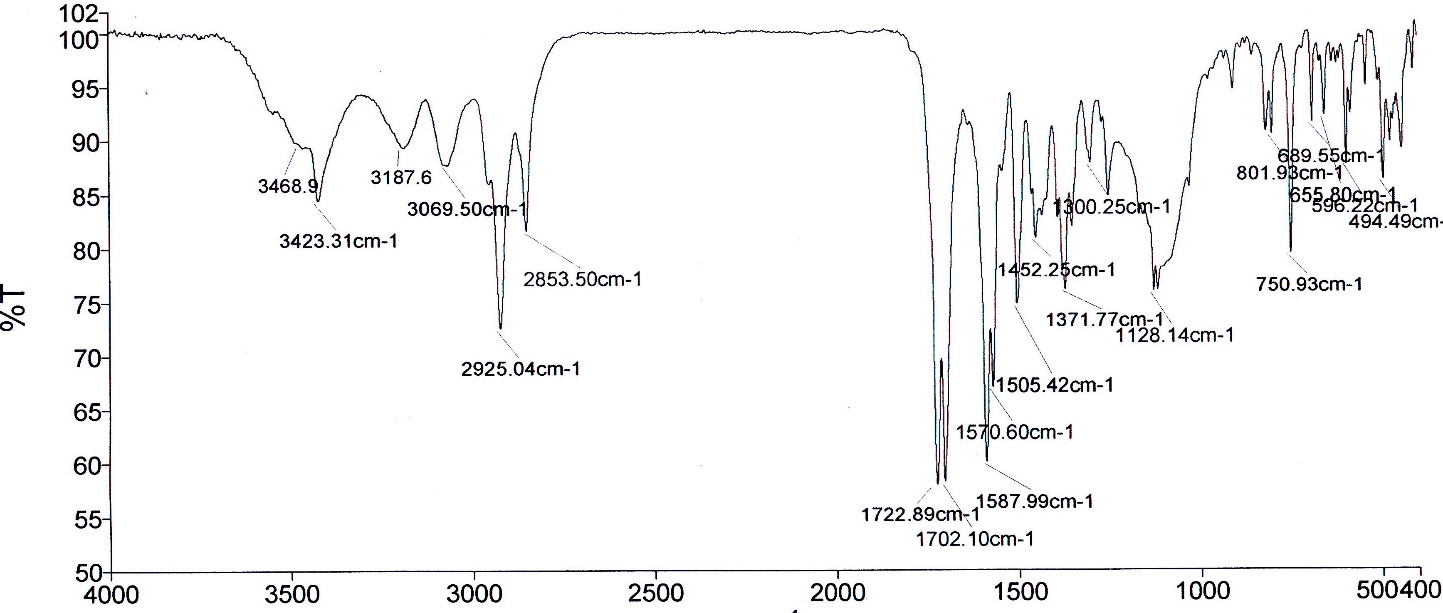
*

FT-IR Spectrum of 3-methyl-4-(naphthalen-2-yl)-1-phenyl-1,8-dihydro-5H-pyrazolo[4',3':5,6]pyrido[2,3-d]pyrimidine-5,7(6H)-dione

*
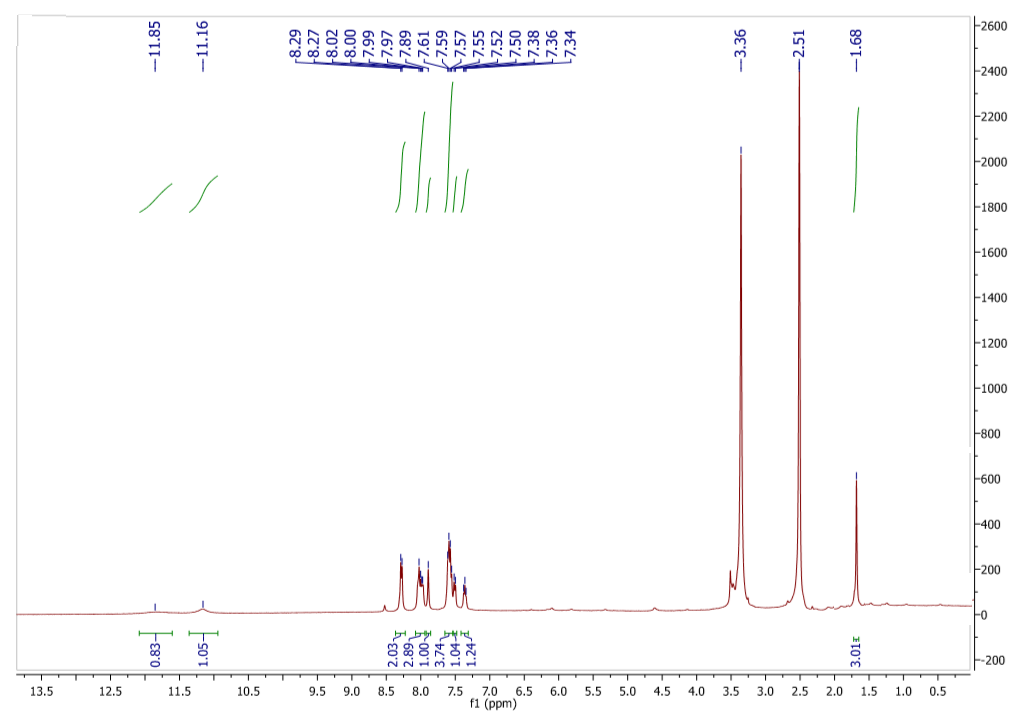
*

^1^H-NMR Spectrum of 3-methyl-4-(naphthalen-2-yl)-1-phenyl-1,8-dihydro-5H-pyrazolo[4',3':5,6]pyrido[2,3-d]pyrimidine-5,7(6H)-dione

*
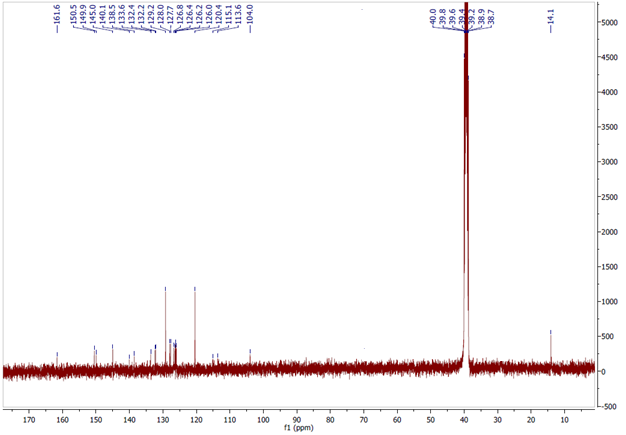
*

^13^C-NMR Spectrum of 3-methyl-4-(naphthalen-2-yl)-1-phenyl-1,8-dihydro-5H-pyrazolo[4',3':5,6]pyrido[2,3-d]pyrimidine-5,7(6H)-dione

*
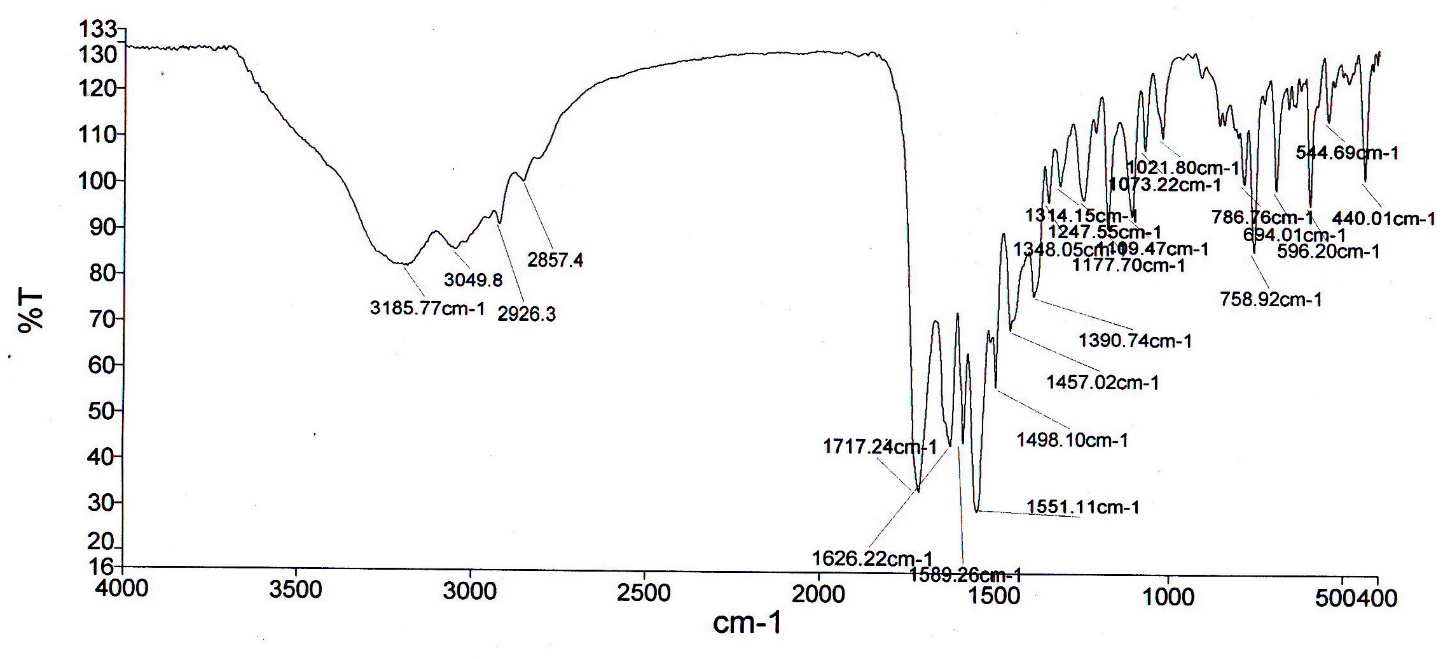
*

FT-IR Spectrum of 3-methyl-1-phenyl-4-(p-tolyl)-1,8-dihydro-5H-pyrazolo[4',3':5,6]pyrido[2,3-d]pyrimidine-5,7(6H)-dion

*L*

*
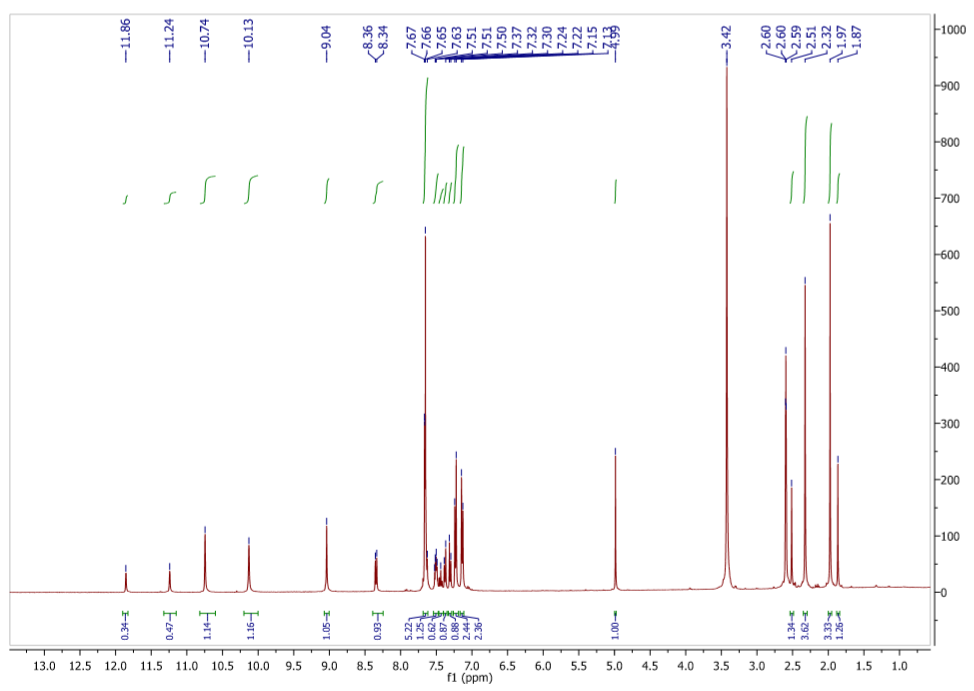
*

^1^H-NMR Spectrum of 3-methyl-1-phenyl-4-(p-tolyl)-1,8-dihydro-5H-pyrazolo[4',3':5,6]pyrido[2,3-d]pyrimidine-5,7(6H)-dion

*
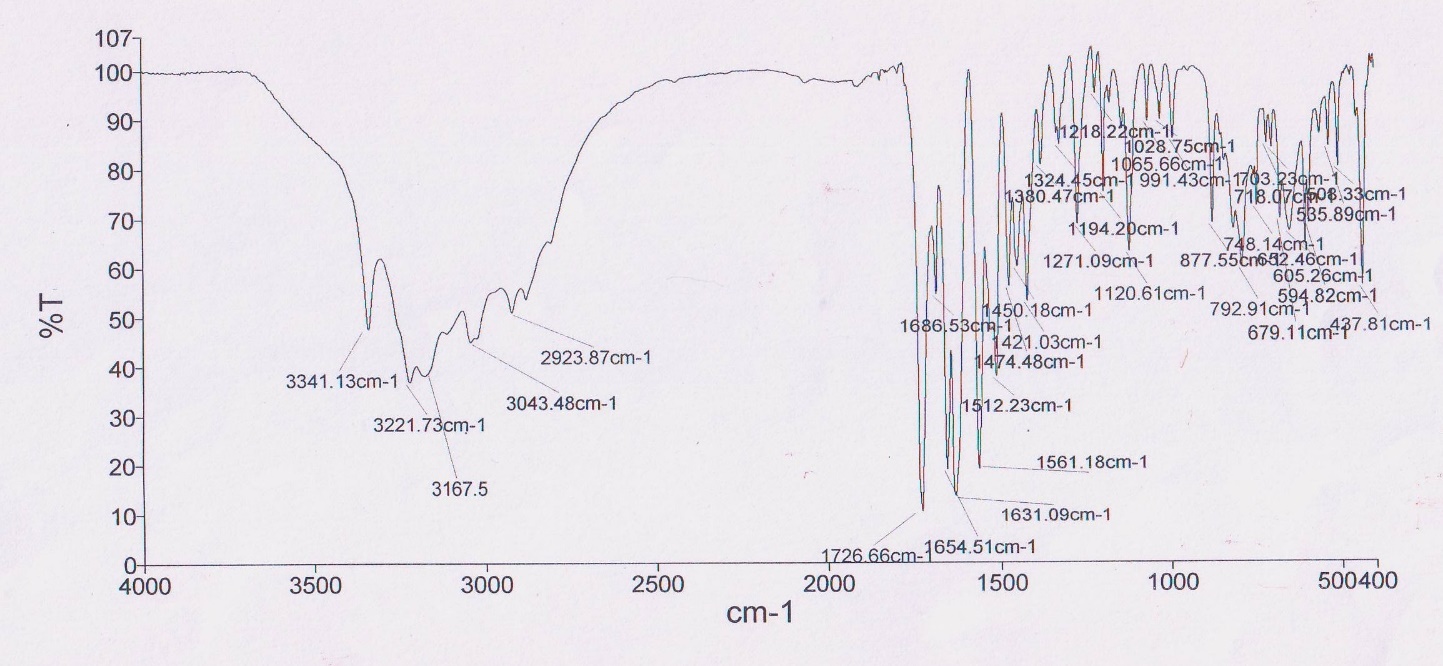
*

FT-IR Spectrum of 3-methyl-4-(p-tolyl)-1,4,8,9-tetrahydro-5H-pyrazolo[4',3':5,6]pyrido[2,3-d]pyrimidine-5,7(6H)-dione

*
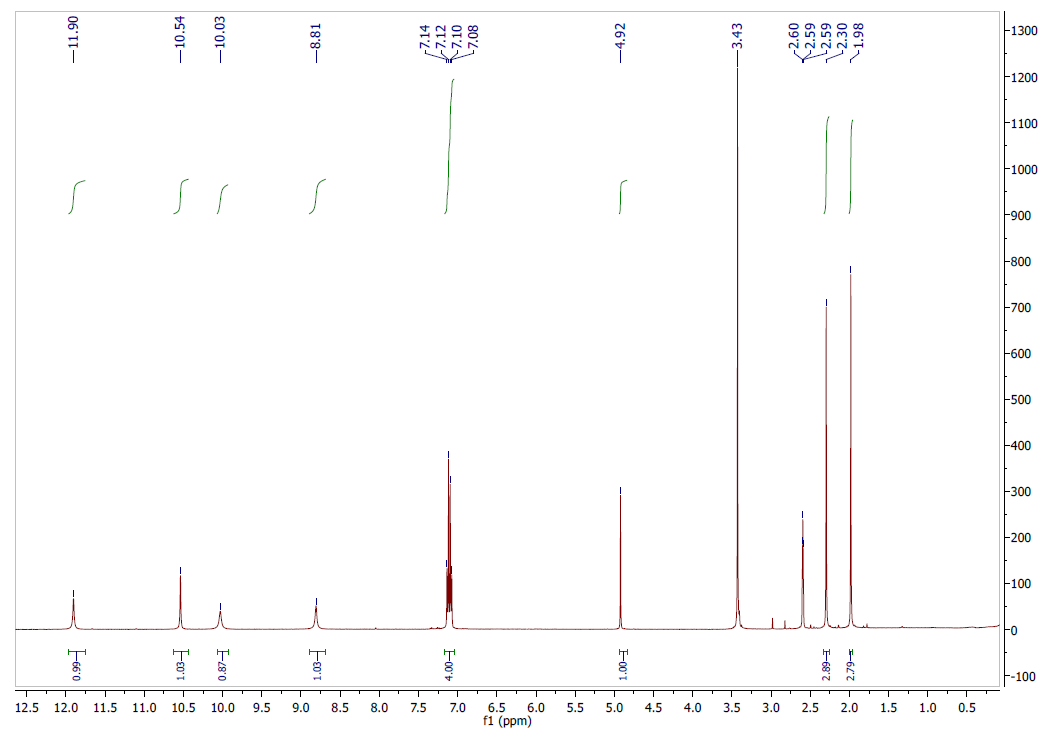
*

^1^H-NMR Spectrum of 3-methyl-4-(p-tolyl)-1,4,8,9-tetrahydro-5H-pyrazolo[4',3':5,6]pyrido[2,3-d]pyrimidine-5,7(6H)-dione

*
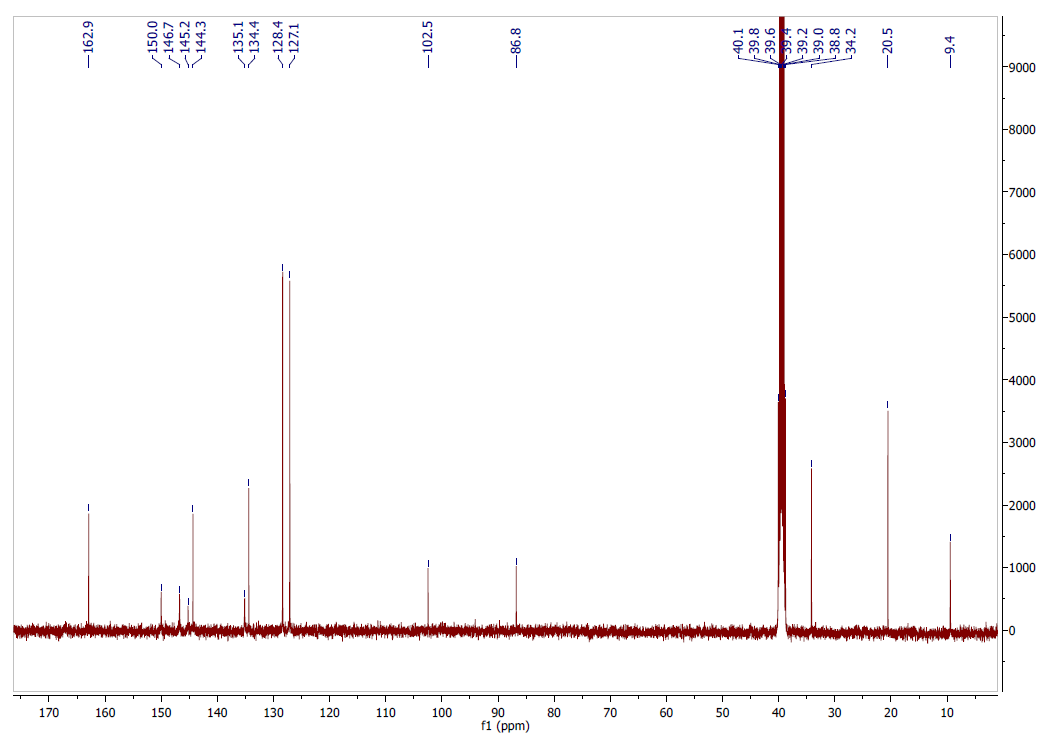
*

^13^C-NMR Spectrum of 3-methyl-4-(p-tolyl)-1,4,8,9-tetrahydro-5H-pyrazolo[4',3':5,6]pyrido[2,3-d]pyrimidine-5,7(6H)-dione

*
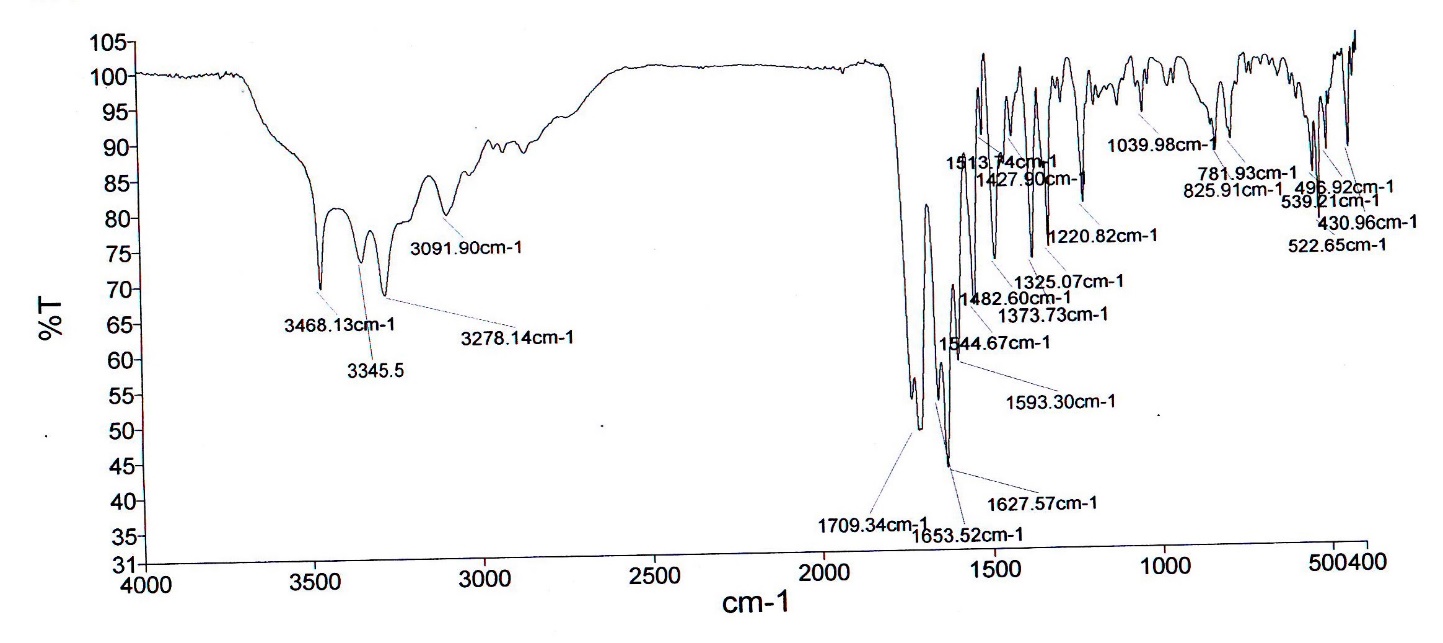
*

FT-IR Spectrum of 3-methyl-4-(4-nitrophenyl)-1,4,8,9-tetrahydro-5H-pyrazolo[4',3':5,6]pyrido[2,3-d]pyrimidine-5,7(6H)-dione

*
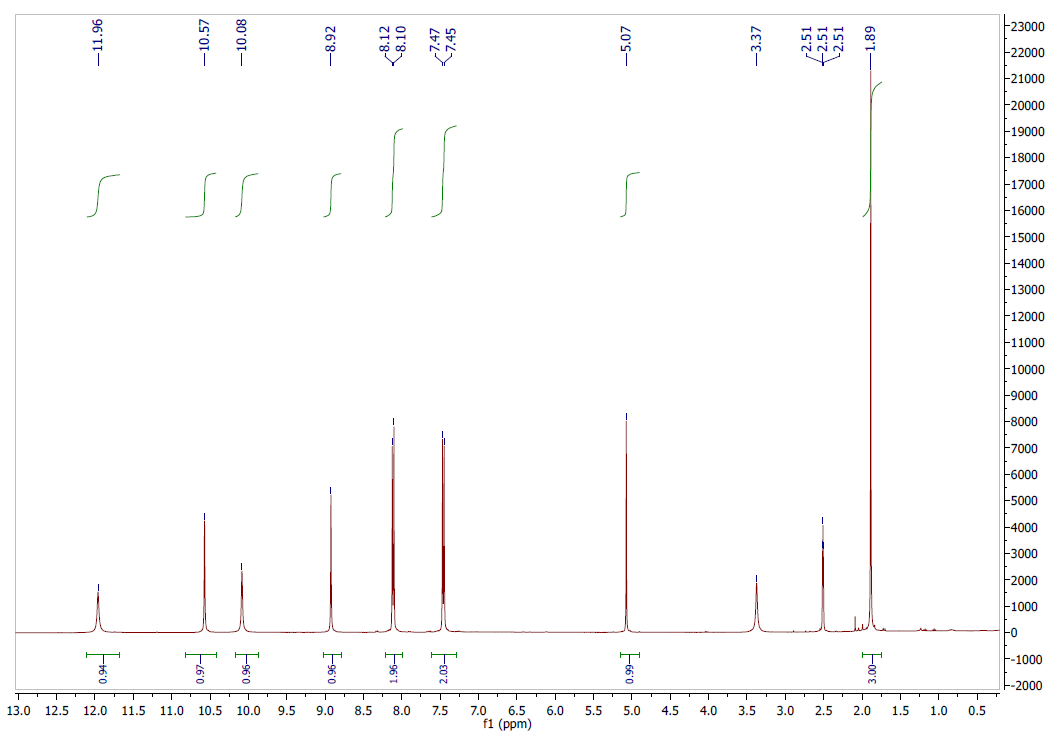
*

^1^H-NMR Spectrum of 3-methyl-4-(4-nitrophenyl)-1,4,8,9-tetrahydro-5H-pyrazolo[4',3':5,6]pyrido[2,3-d]pyrimidine-5,7(6H)-dione

*
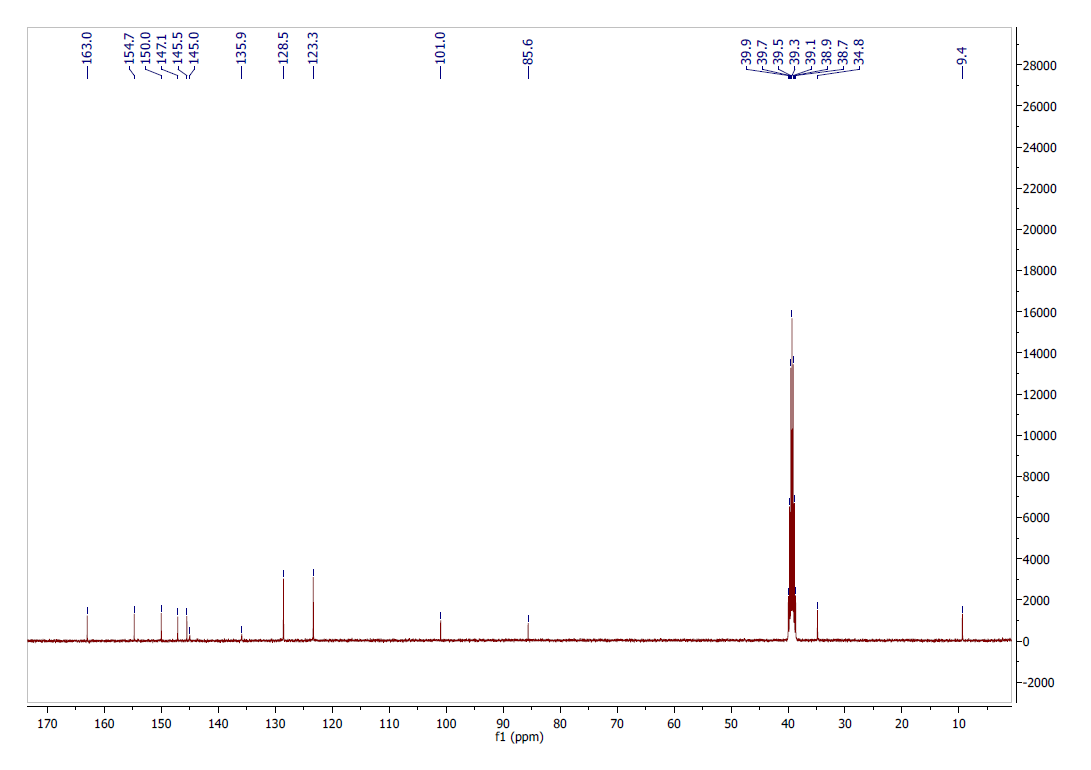
*

^13^C-NMR Spectrum of 3-methyl-4-(4-nitrophenyl)-1,4,8,9-tetrahydro-5H-pyrazolo[4',3':5,6]pyrido[2,3-d]pyrimidine-5,7(6H)-dione

*
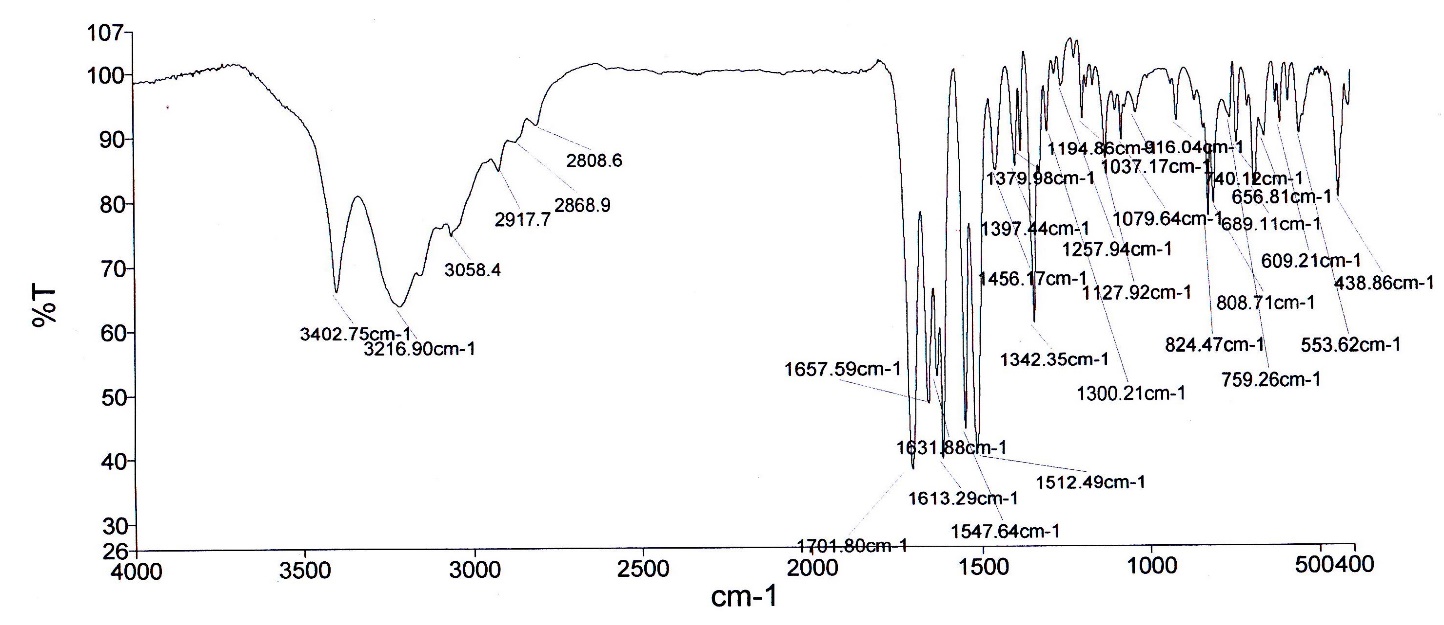
*

FT-IR Spectrum of
3-methyl-4-(3-nitrophenyl)-1,4,8,9-tetrahydro-5H-pyrazolo[4',3':5,6]pyrido[2,3-d]pyrimidine-5,7(6H)-dione

*
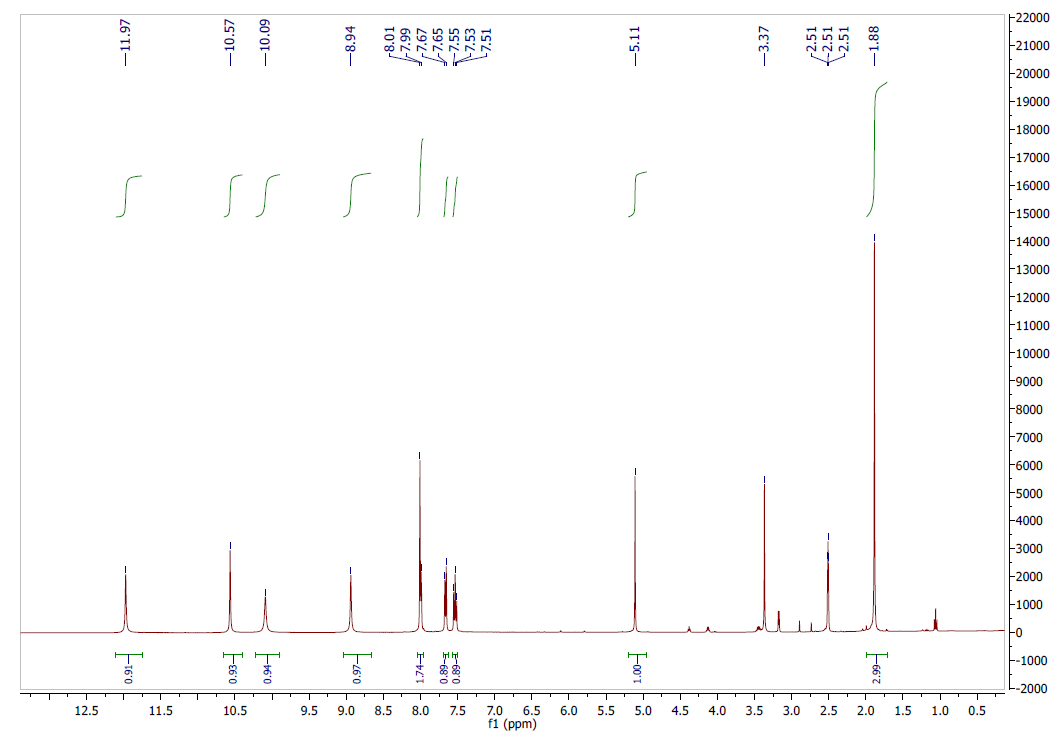
*

^1^H-NMR Spectrum of 3-methyl-4-(3-nitrophenyl)-1,4,8,9-tetrahydro-5H-pyrazolo[4',3':5,6]pyrido[2,3-d]pyrimidine-5,7(6H)-dione

*
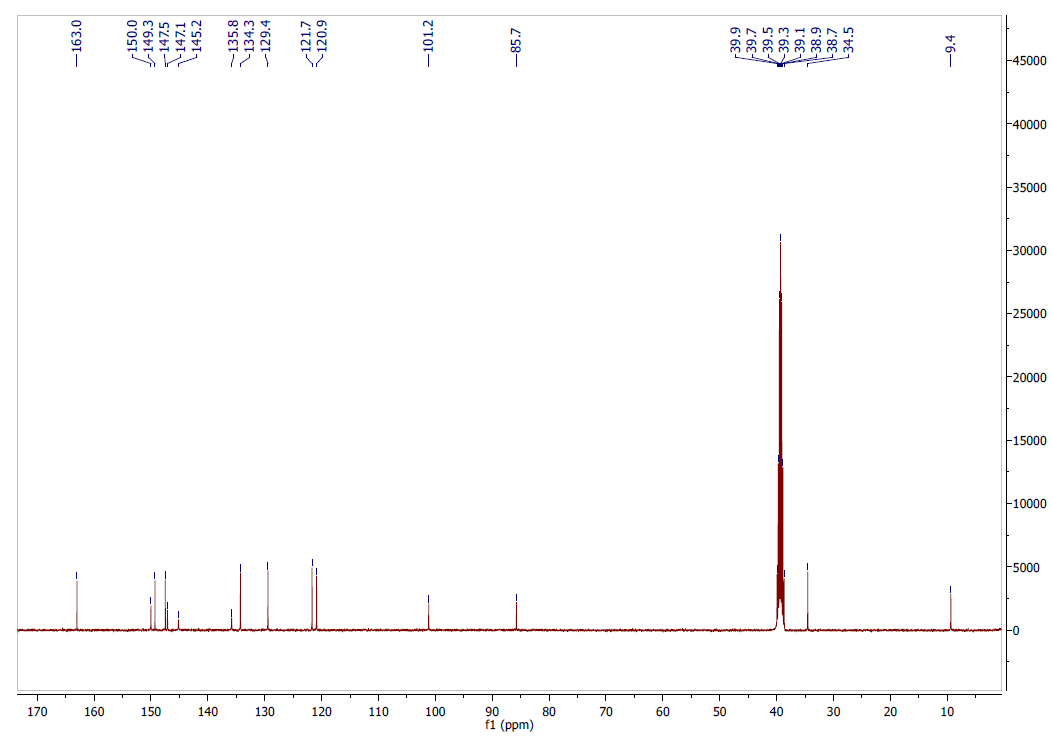
*

^13^C-NMR Spectrum of 3-methyl-4-(3-nitrophenyl)-1,4,8,9-tetrahydro-5H-pyrazolo[4',3':5,6]pyrido[2,3-d]pyrimidine-5,7(6H)-dione

*
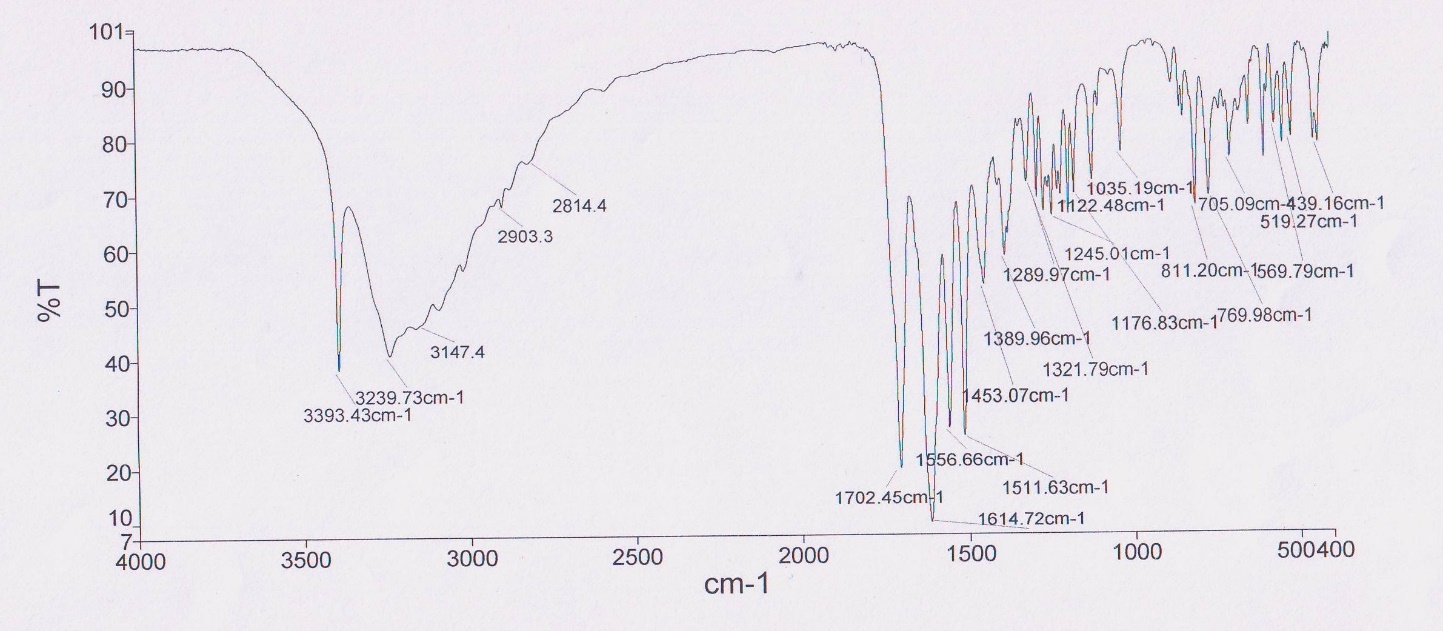
*

FT-IR Spectrum of 4-(4-hydroxyphenyl)-3-methyl-1,4,8,9-tetrahydro-5H-pyrazolo[4',3':5,6]pyrido[2,3-d]pyrimidine-5,7(6H)-dione

*
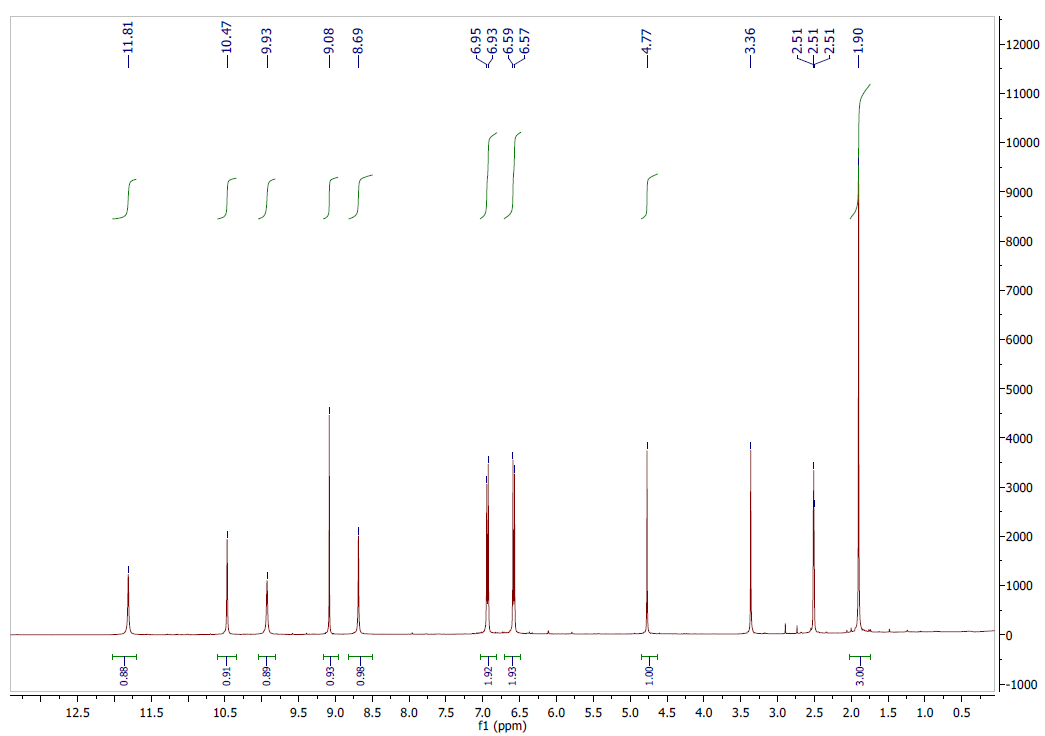
*

^1^H-NMR Spectrum of 4-(4-hydroxyphenyl)-3-methyl-1,4,8,9-tetrahydro-5H-pyrazolo[4',3':5,6]pyrido[2,3-d]pyrimidine-5,7(6H)-dione

*
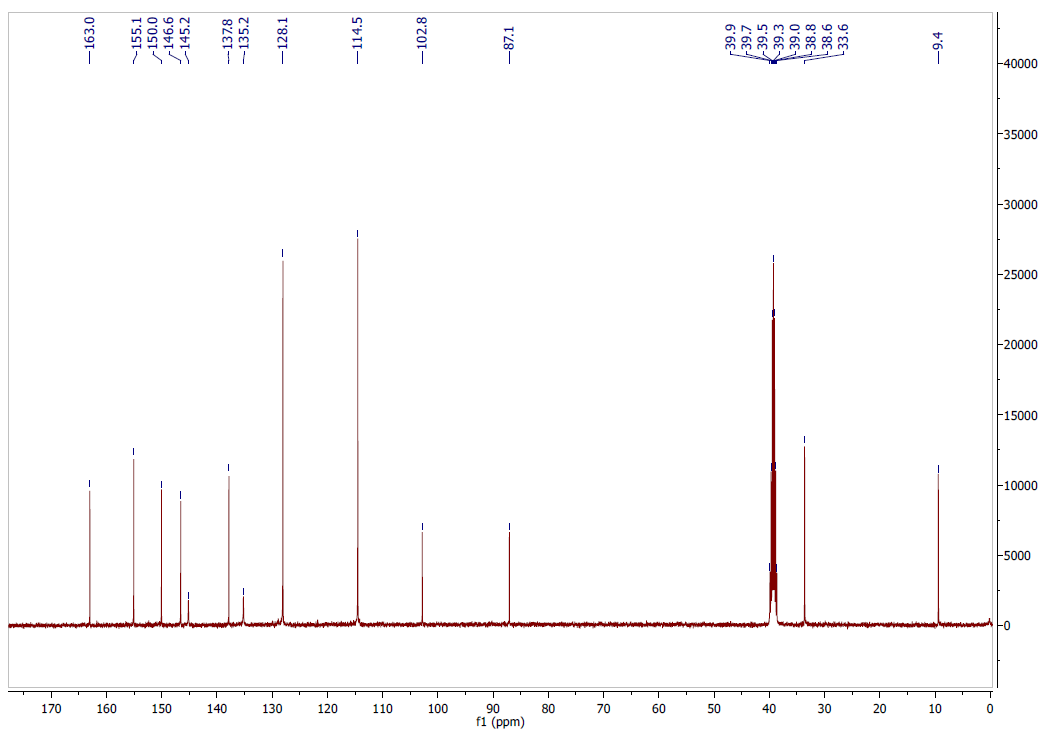
*

^13^C-NMR Spectrum of 4-(4-hydroxyphenyl)-3-methyl-1,4,8,9-tetrahydro-5H-pyrazolo[4',3':5,6]pyrido[2,3-d]pyrimidine-5,7(6H)-dione

*
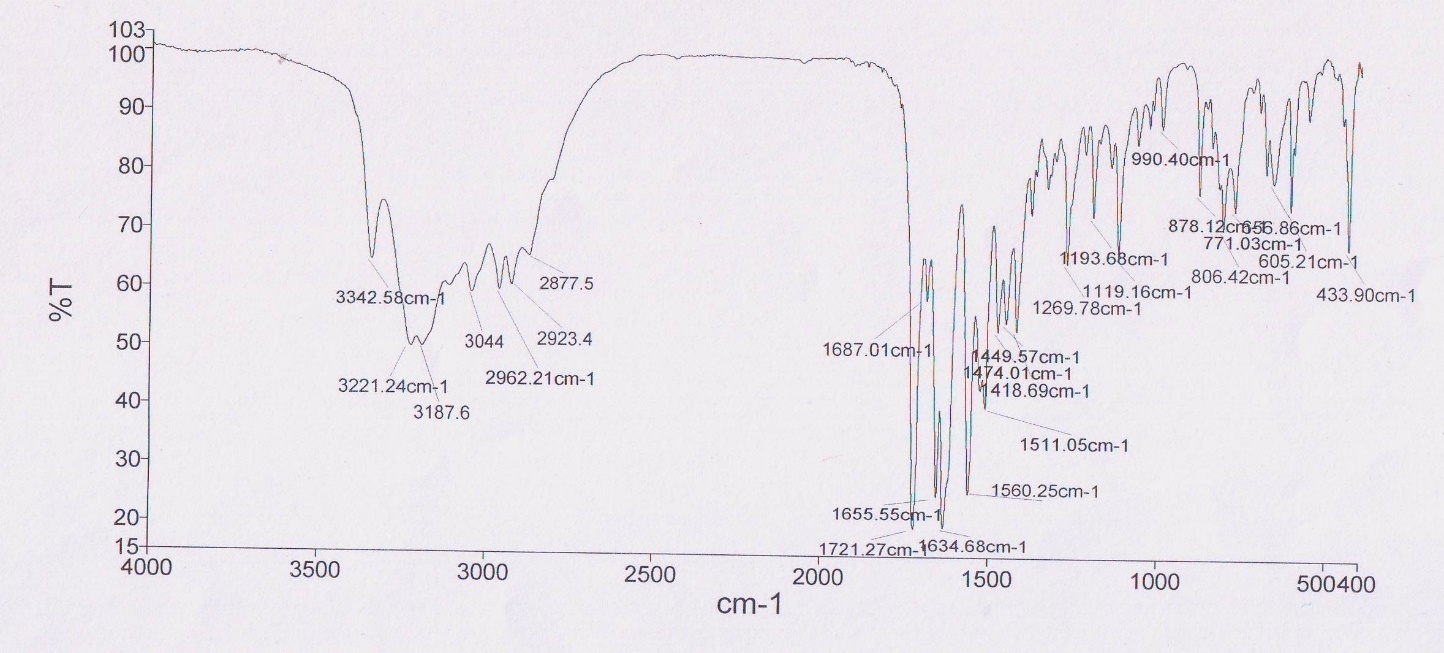
*

FT-IR Spectrum of 4-(4-isopropylphenyl)-3-methyl-1,4,8,9-tetrahydro-5H-pyrazolo[4',3':5,6]pyrido[2,3-d]pyrimidine-5,7(6H)-dione

*
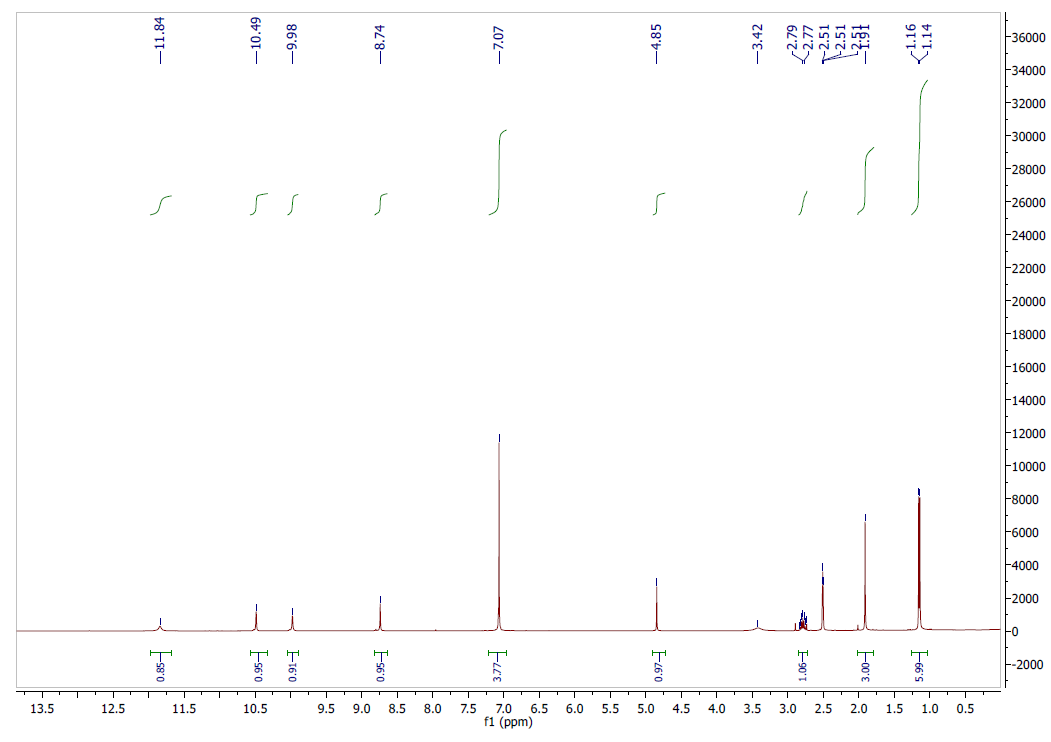
*

^1^H-NMR Spectrum of 4-(4-isopropylphenyl)-3-methyl-1,4,8,9-tetrahydro-5H-pyrazolo[4',3':5,6]pyrido[2,3-d]pyrimidine-5,7(6H)-dione

*
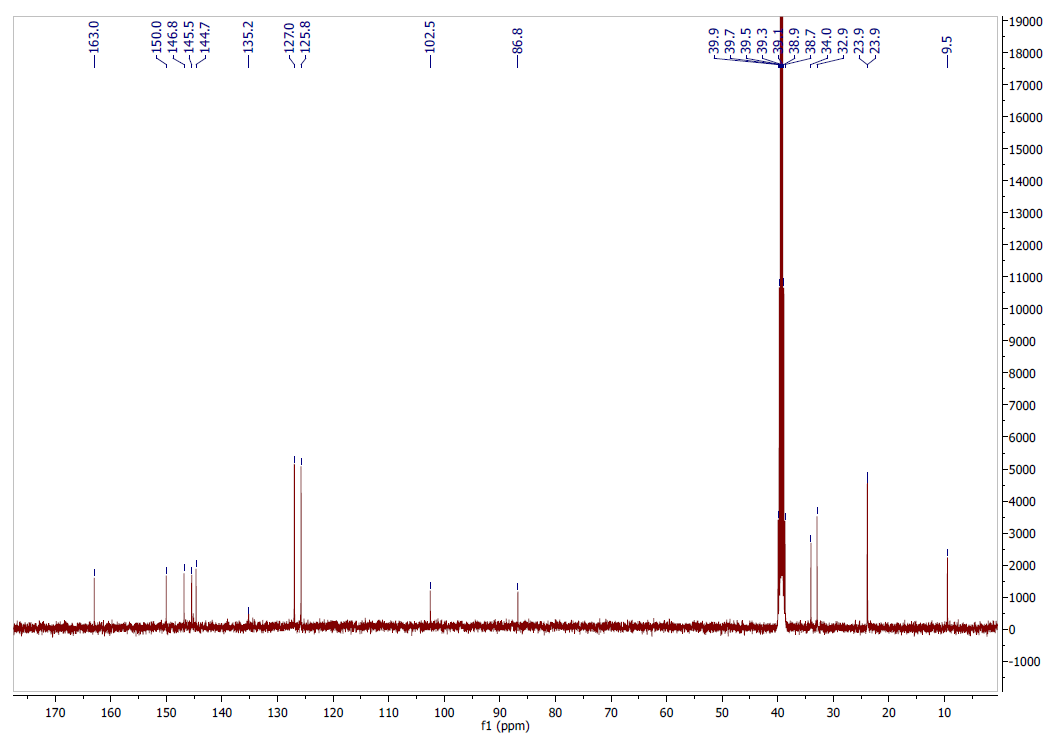
*

^13^C-NMR Spectrum of 4-(4-isopropylphenyl)-3-methyl-1,4,8,9-tetrahydro-5H-pyrazolo[4',3':5,6]pyrido[2,3-d]pyrimidine-5,7(6H)-dione

*
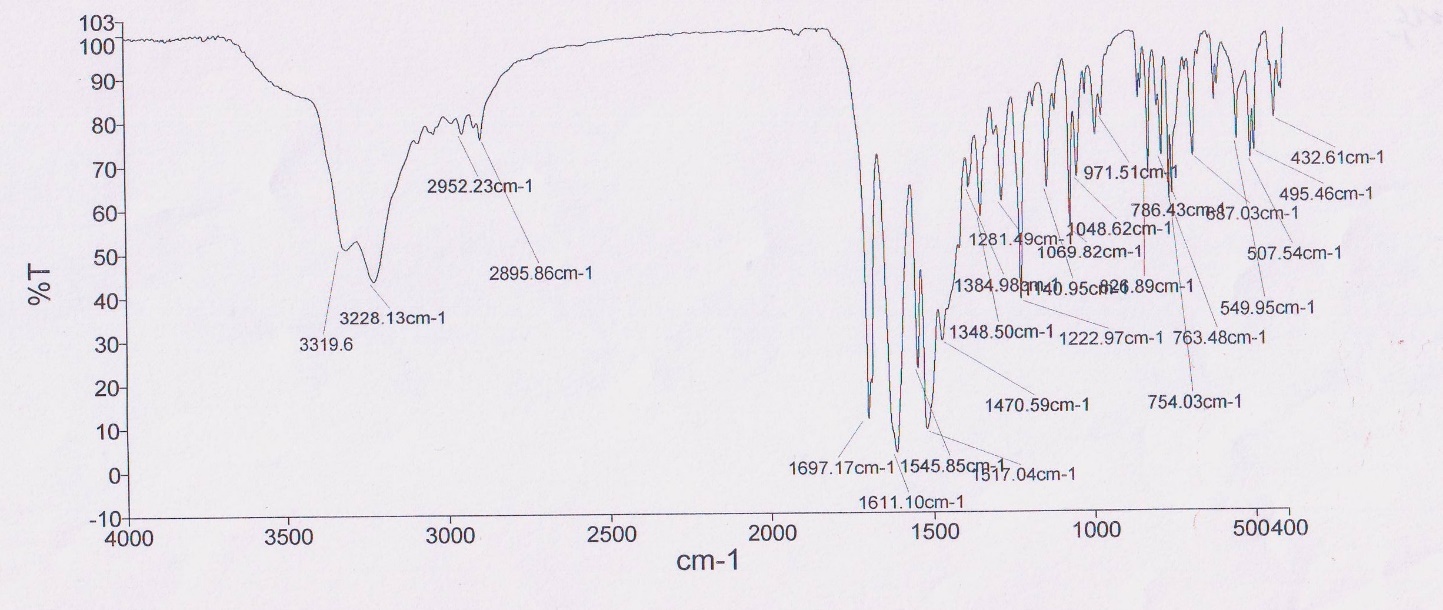
*

FT-IR Spectrum of 3,6,8-trimethyl-4-(p-tolyl)-1,4,8,9-tetrahydro-5H-pyrazolo[4',3':5,6]pyrido[2,3-d]pyrimidine-5,7(6H)-dione

*
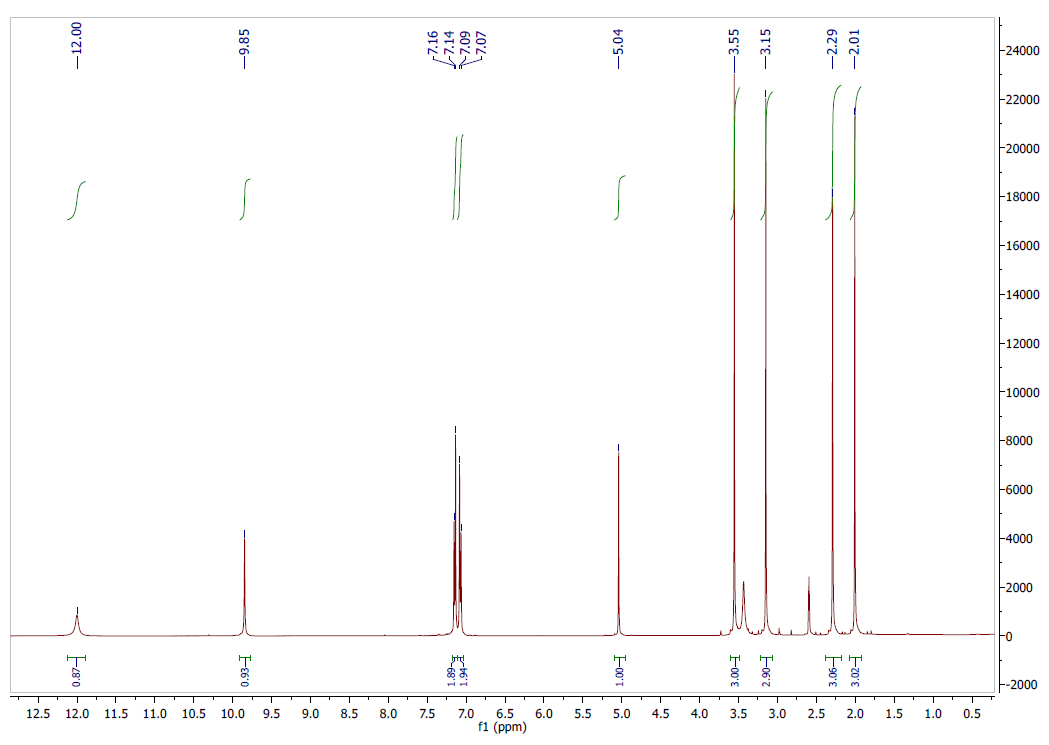
*

^1^H-NMR Spectrum of 3,6,8-trimethyl-4-(p-tolyl)-1,4,8,9-tetrahydro-5H-pyrazolo[4',3':5,6]pyrido[2,3-d]pyrimidine-5,7(6H)-dione

*
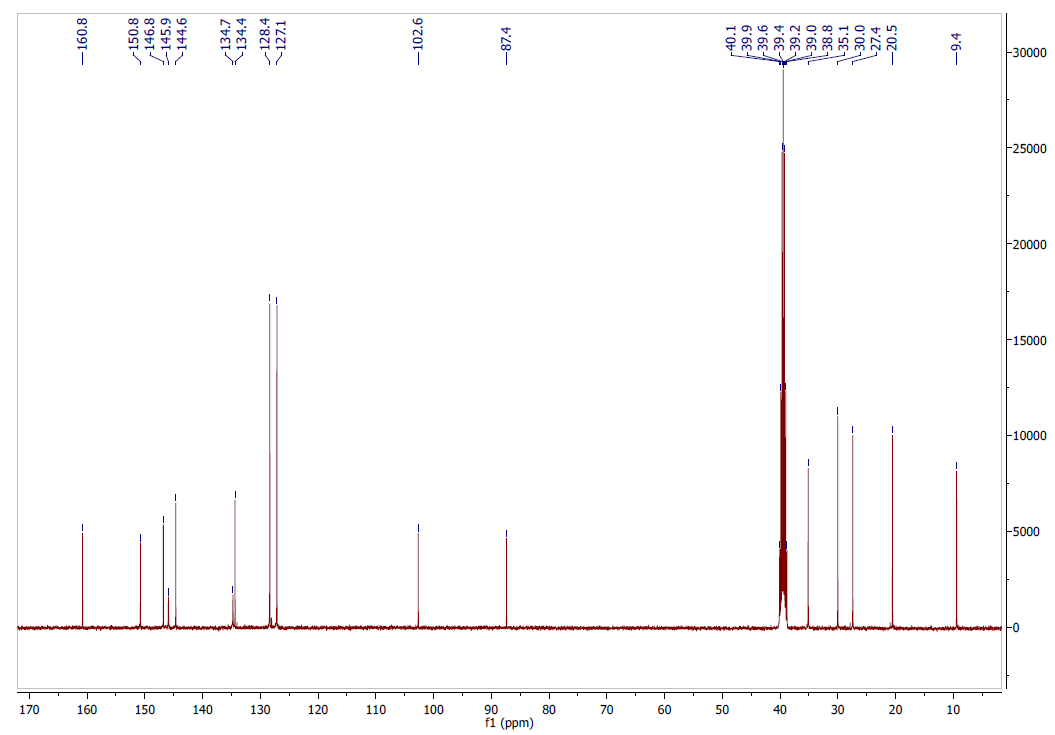
*

^13^C-NMR Spectrum of 3,6,8-trimethyl-4-(p-tolyl)-1,4,8,9-tetrahydro-5H-pyrazolo[4',3':5,6]pyrido[2,3-d]pyrimidine-5,7(6H)-dione

*
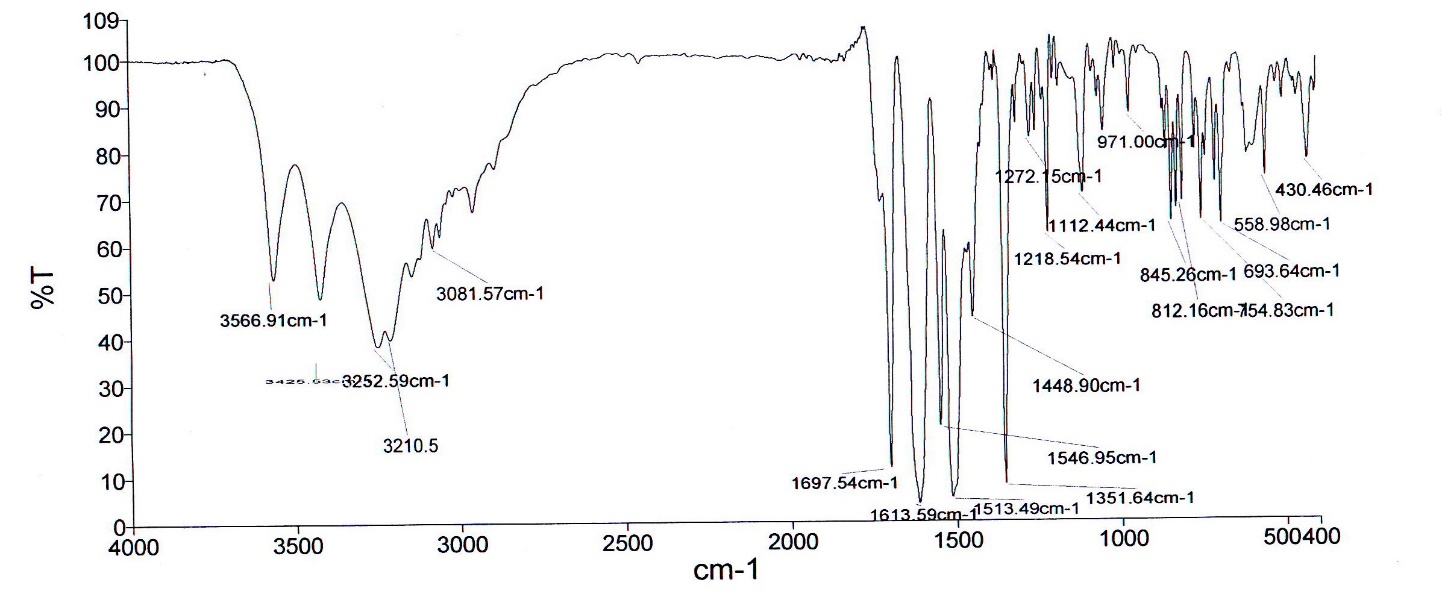
*

FT-IR Spectrum of 3,6,8-trimethyl-4-(4-nitrophenyl)-1,4,8,9-tetrahydro-5H-pyrazolo[4',3':5,6]pyrido[2,3-d]pyrimidine-5,7(6H)-dione

*
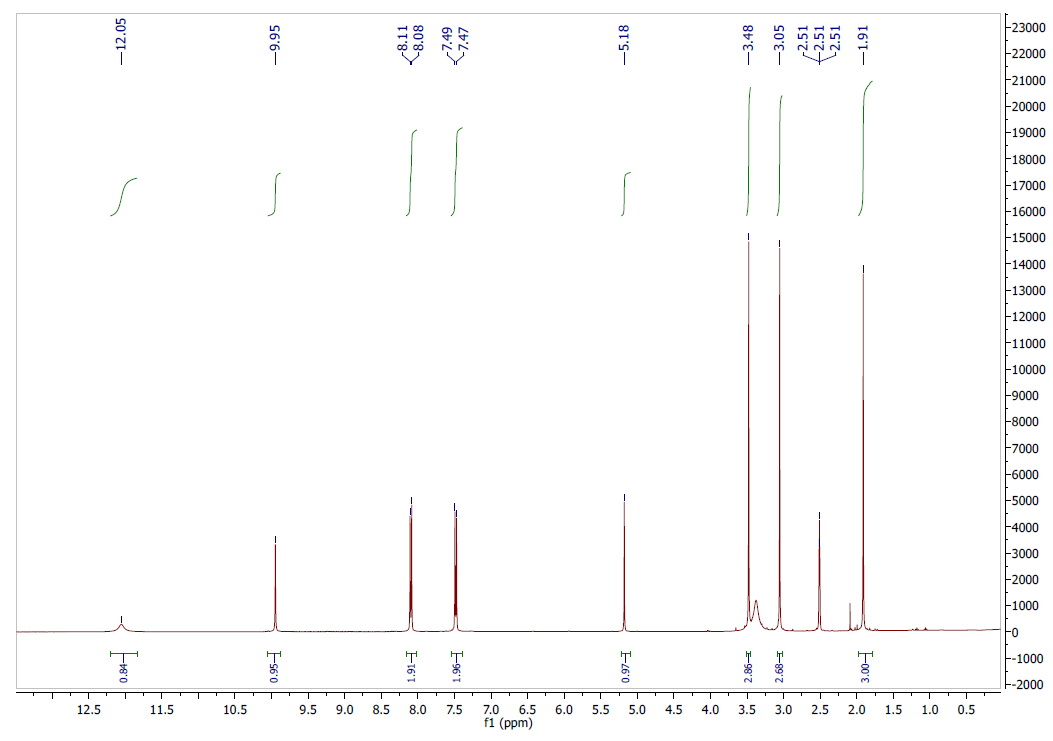
*

^1^H-NMR Spectrum of 3,6,8-trimethyl-4-(4-nitrophenyl)-1,4,8,9-tetrahydro-5H-pyrazolo[4',3':5,6]pyrido[2,3-d]pyrimidine-5,7(6H)-dione

*
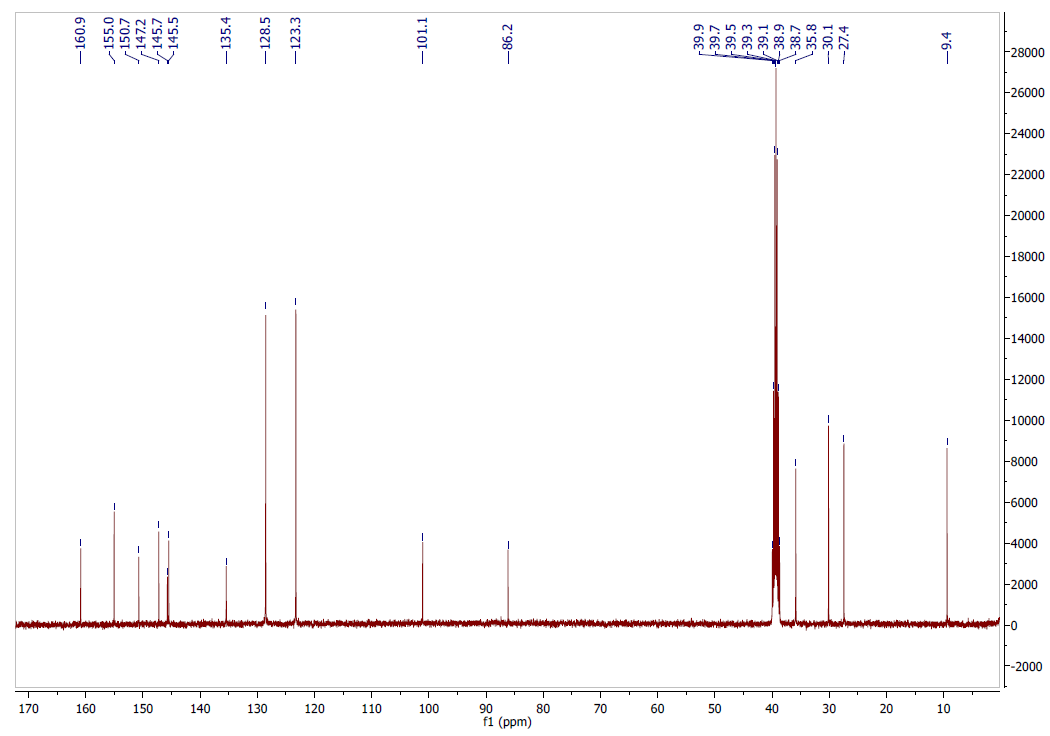
*

^13^C-NMR Spectrum of 3,6,8-trimethyl-4-(4-nitrophenyl)-1,4,8,9-tetrahydro-5H-pyrazolo[4',3':5,6]pyrido[2,3-d]pyrimidine-5,7(6H)-dione

*
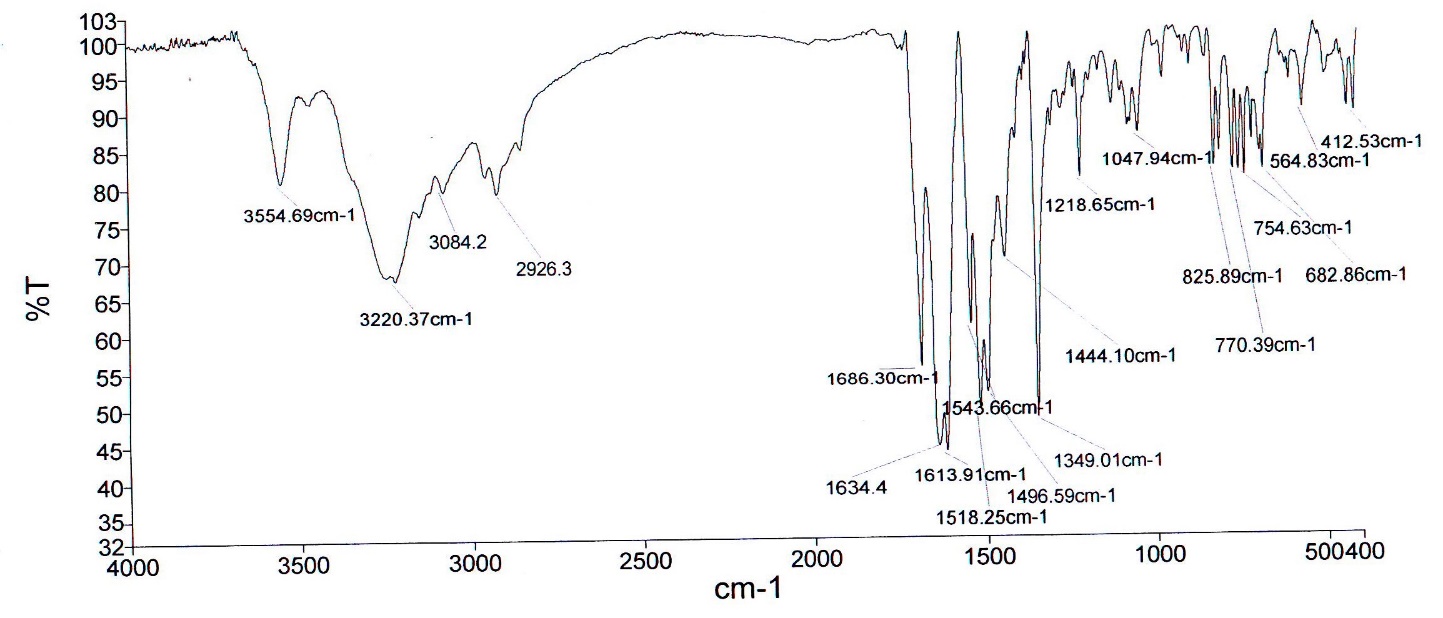
*

FT-IR Spectrum of 3,6,8-trimethyl-4-(3-nitrophenyl)-1,4,8,9-tetrahydro-5H-pyrazolo[4',3':5,6]pyrido[2,3-d]pyrimidine-5,7(6H)-dione

*
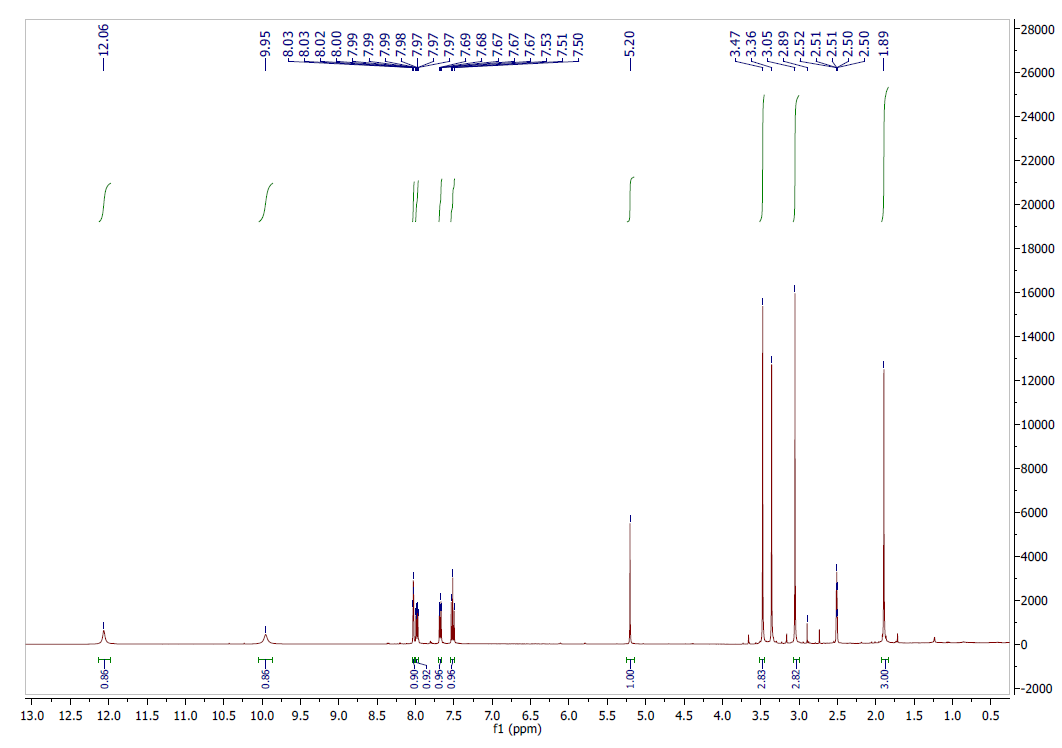
*

^1^H-NMR Spectrum of 3,6,8-trimethyl-4-(3-nitrophenyl)-1,4,8,9-tetrahydro-5H-pyrazolo[4',3':5,6]pyrido[2,3-d]pyrimidine-5,7(6H)-dione

*
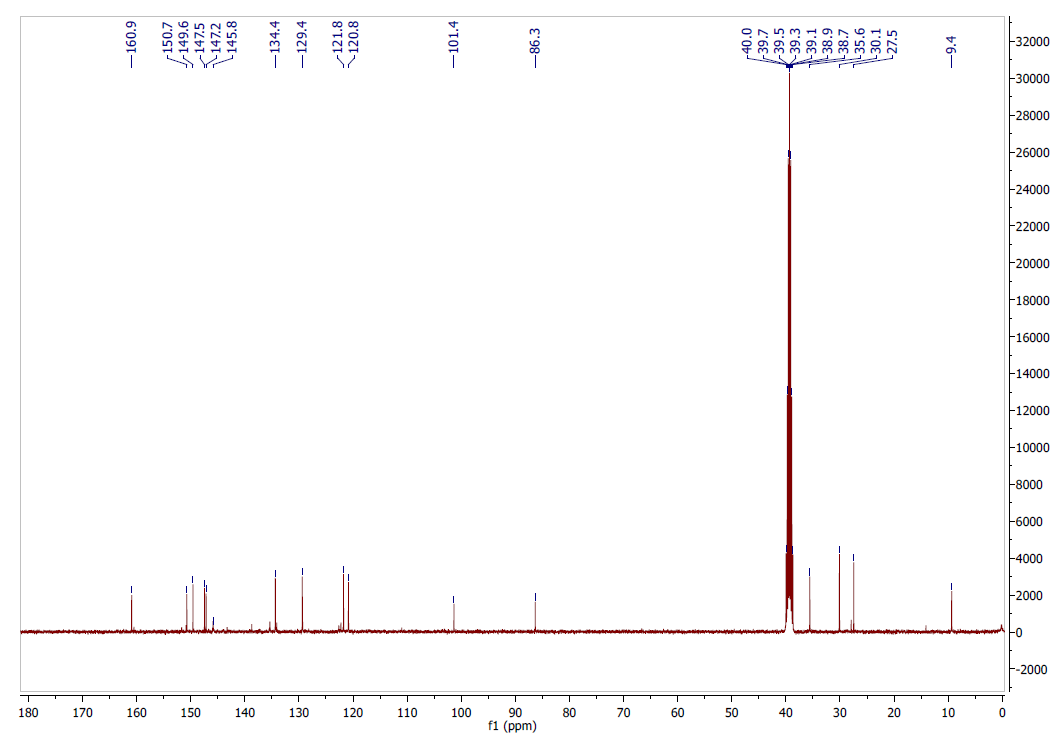
*

^13^C-NMR Spectrum of 3,6,8-trimethyl-4-(3-nitrophenyl)-1,4,8,9-tetrahydro-5H-pyrazolo[4',3':5,6]pyrido[2,3-d]pyrimidine-5,7(6H)-dione

*
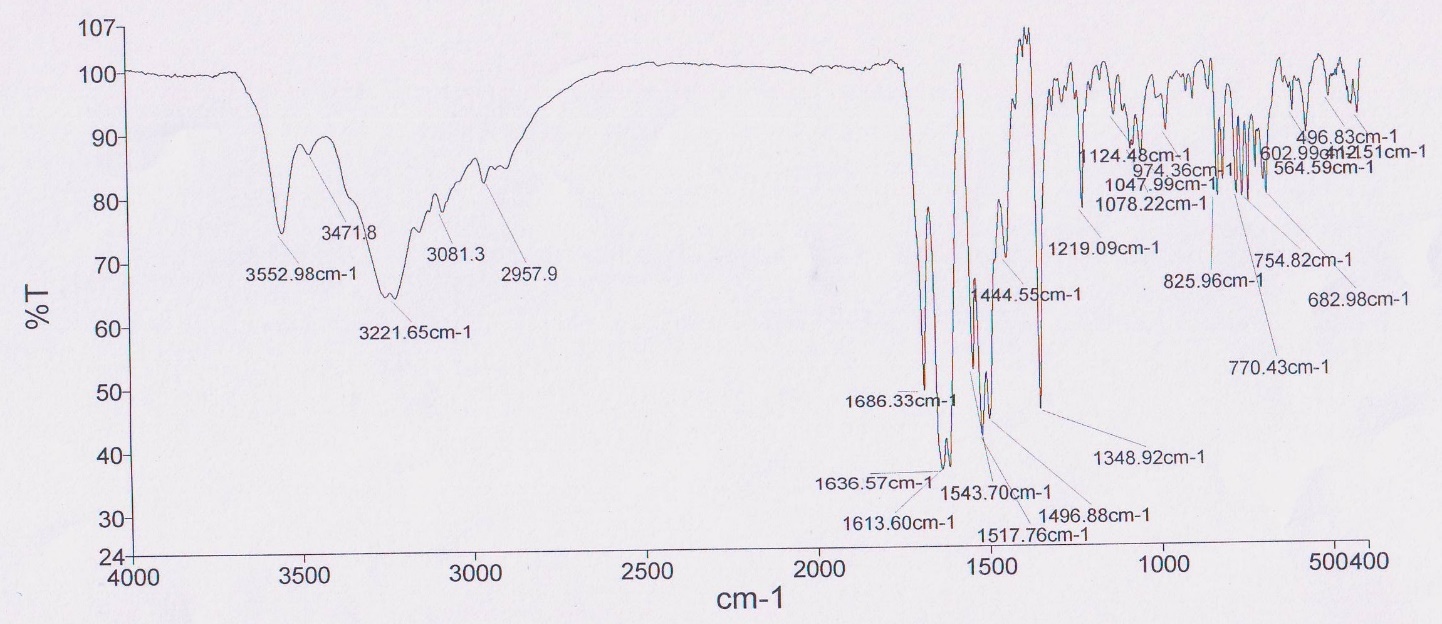
*

FT-IR Spectrum of 4-(2,6-dichlorophenyl)-3,6,8-trimethyl-1,4,8,9-tetrahydro-5H-pyrazolo[4',3':5,6]pyrido[2,3-d]pyrimidine-5,7(6H)-dione

*
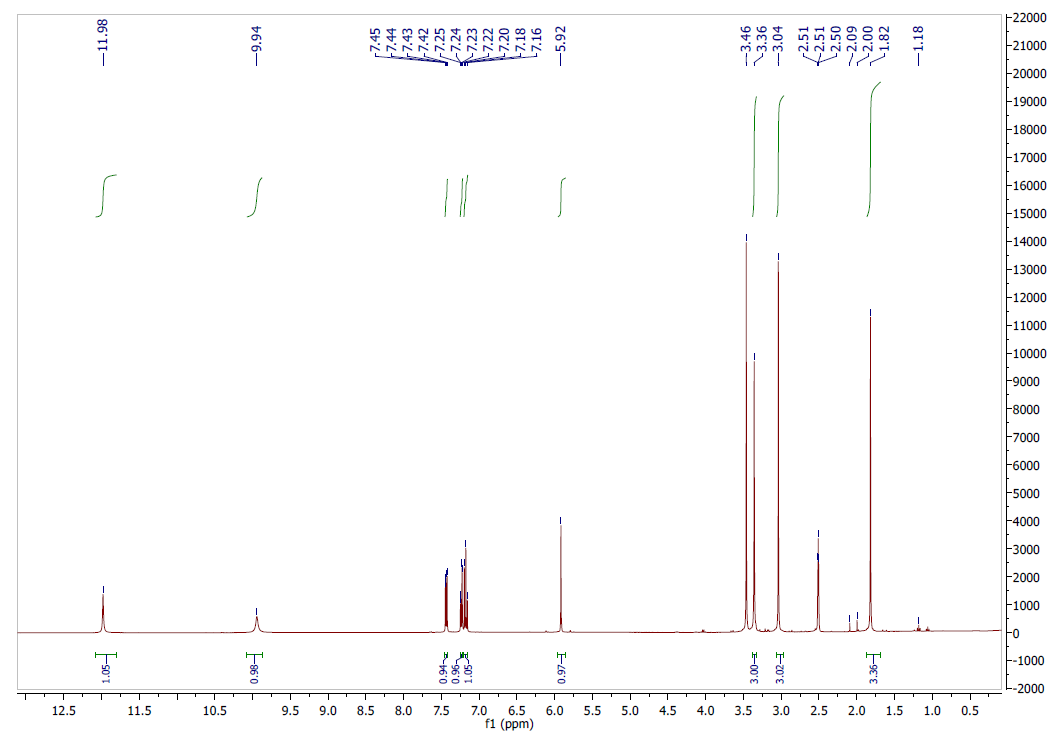
*

^1^H-NMR Spectrum of 4-(2,6-dichlorophenyl)-3,6,8-trimethyl-1,4,8,9-tetrahydro-5H-pyrazolo[4',3':5,6]pyrido[2,3-d]pyrimidine-5,7(6H)-dione

*
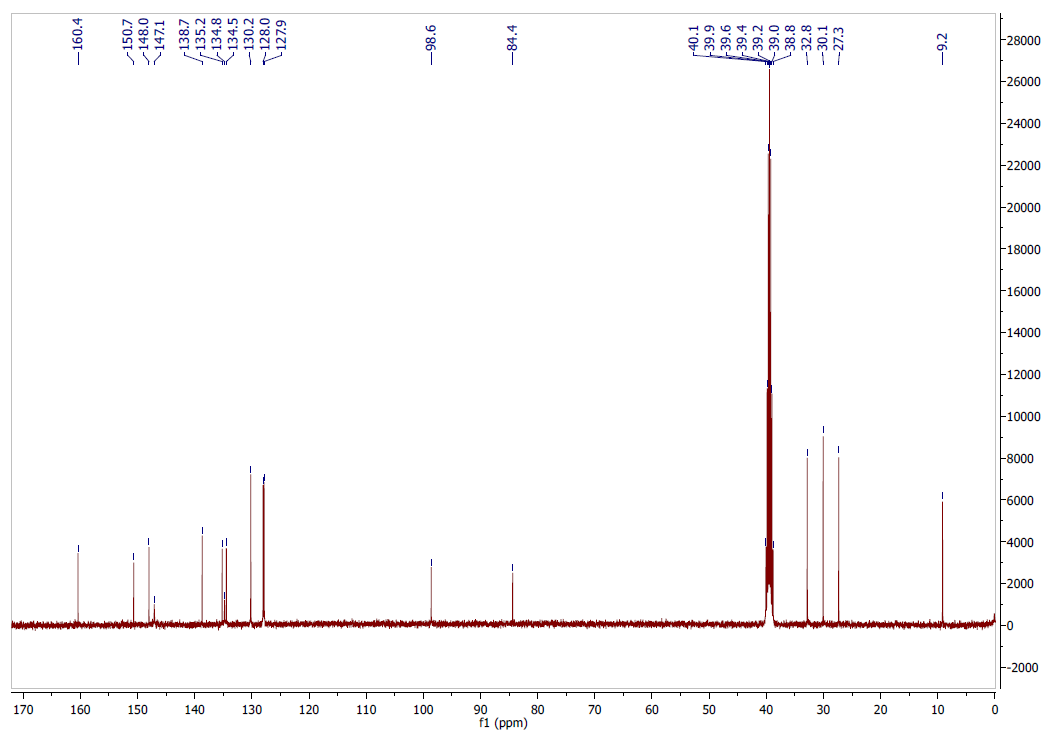
*

^13^C-NMR Spectrum of 4-(2,6-dichlorophenyl)-3,6,8-trimethyl-1,4,8,9-tetrahydro-5H-pyrazolo[4',3':5,6]pyrido[2,3-d]pyrimidine-5,7(6H)-dione

*
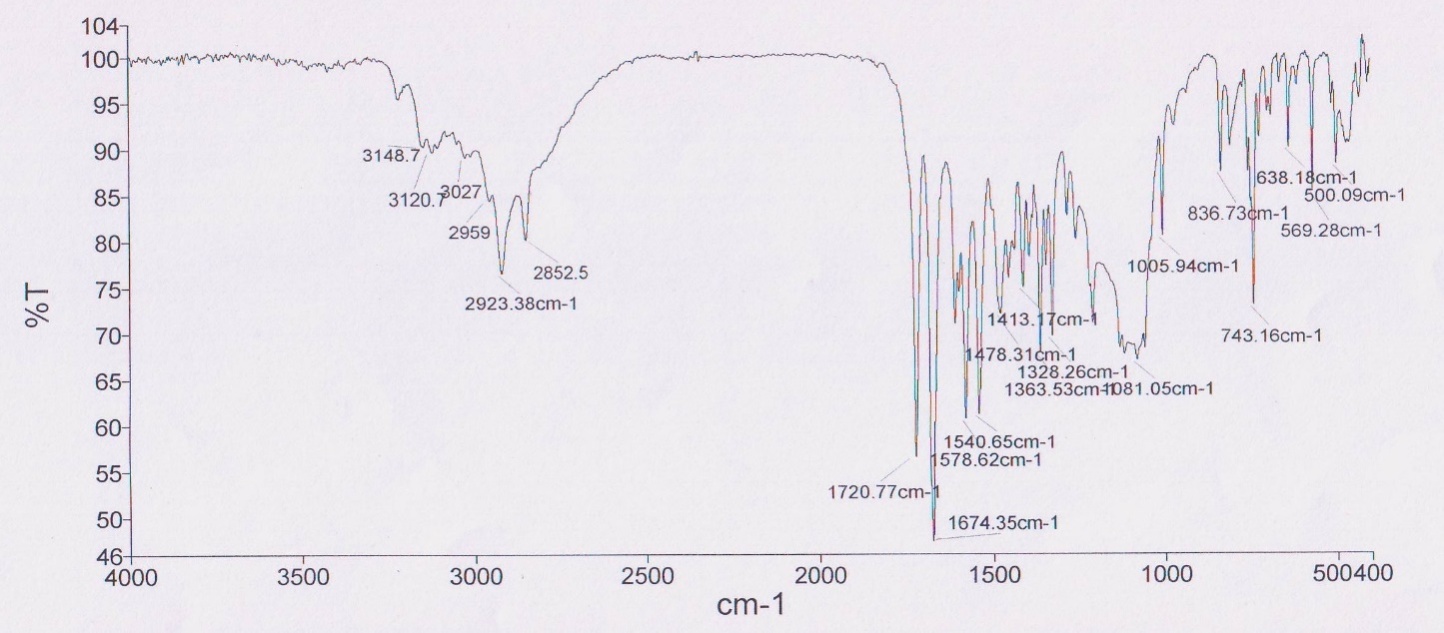
*

FT-IR Spectrum of 4-(2-chlorophenyl)-3,6,8-trimethyl-1,8-dihydro-5H-pyrazolo[4',3':5,6]pyrido[2,3-d]pyrimidine-5,7(6H)-dione

*
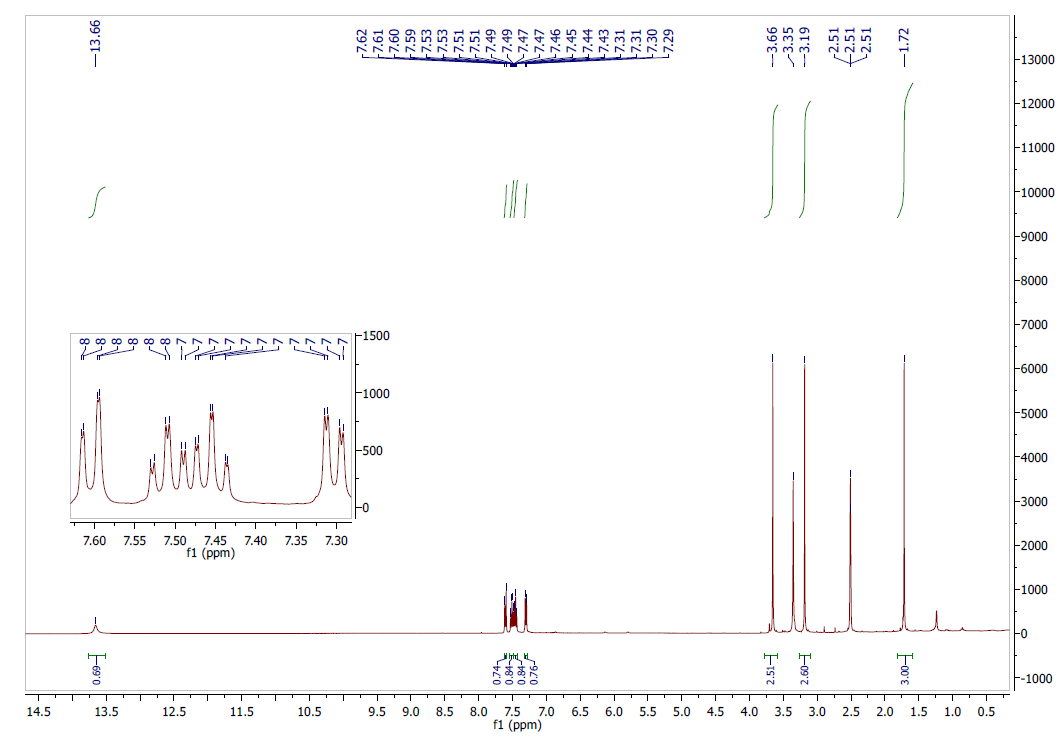
*

^1^H-NMR Spectrum of 4-(2-chlorophenyl)-3,6,8-trimethyl-1,8-dihydro-5H-pyrazolo[4',3':5,6]pyrido[2,3-d]pyrimidine-5,7(6H)-dione

*
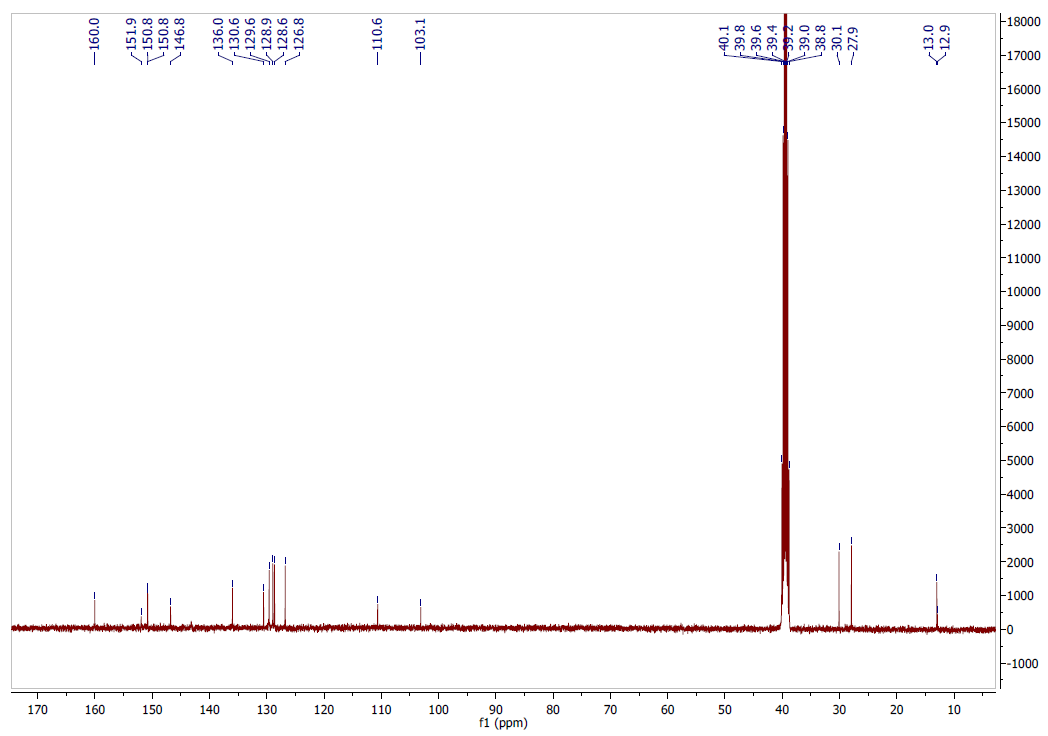
*

^13^C-NMR Spectrum of 4-(2-chlorophenyl)-3,6,8-trimethyl-1,8-dihydro-5H-pyrazolo[4',3':5,6]pyrido[2,3-d]pyrimidine-5,7(6H)-dione

*
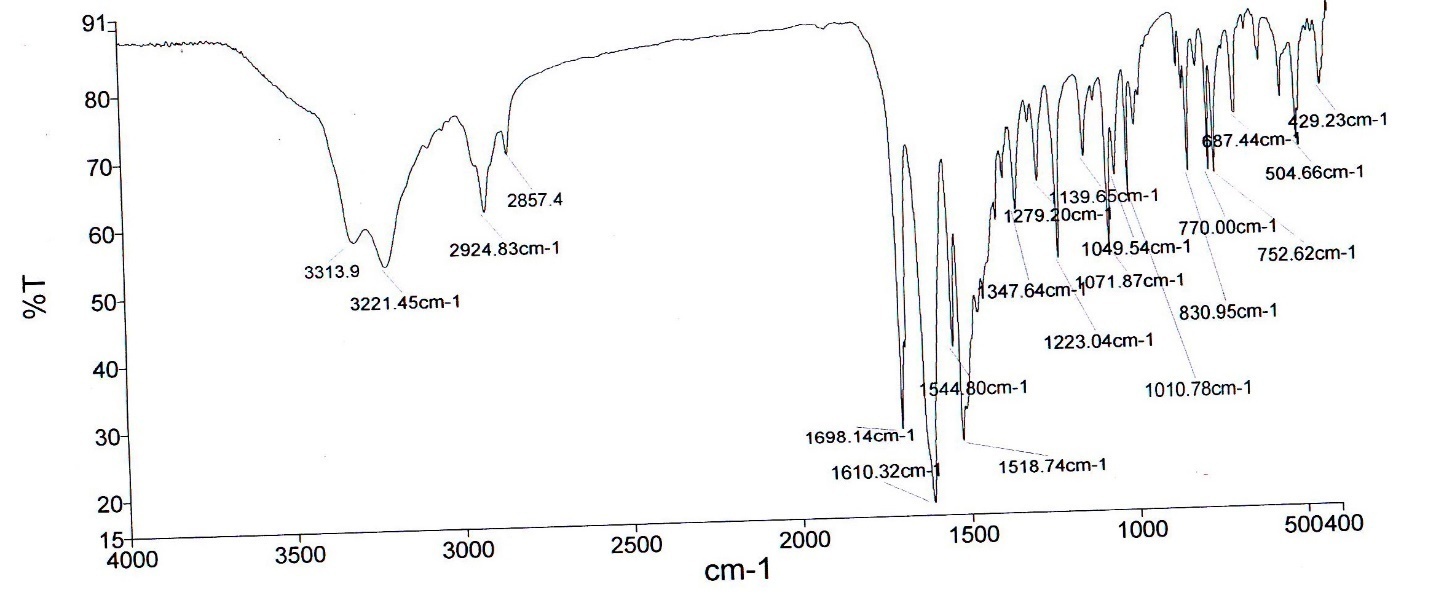
*

FT-IR Spectrum of 4-(4-bromophenyl)-3,6,8-trimethyl-1,4,8,9-tetrahydro-5H-pyrazolo[4',3':5,6]pyrido[2,3-d]pyrimidine-5,7(6H)-dione

*
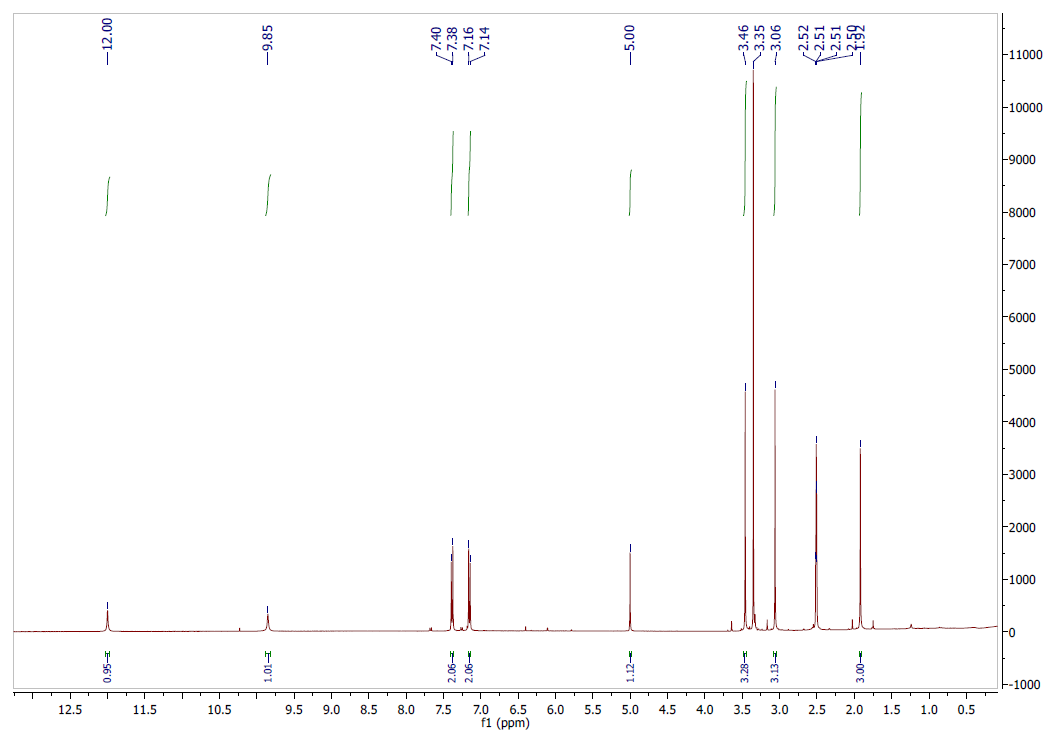
*

^1^H-NMR Spectrum of 4-(4-bromophenyl)-3,6,8-trimethyl-1,4,8,9-tetrahydro-5H-pyrazolo[4',3':5,6]pyrido[2,3-d]pyrimidine-5,7(6H)-dione

*
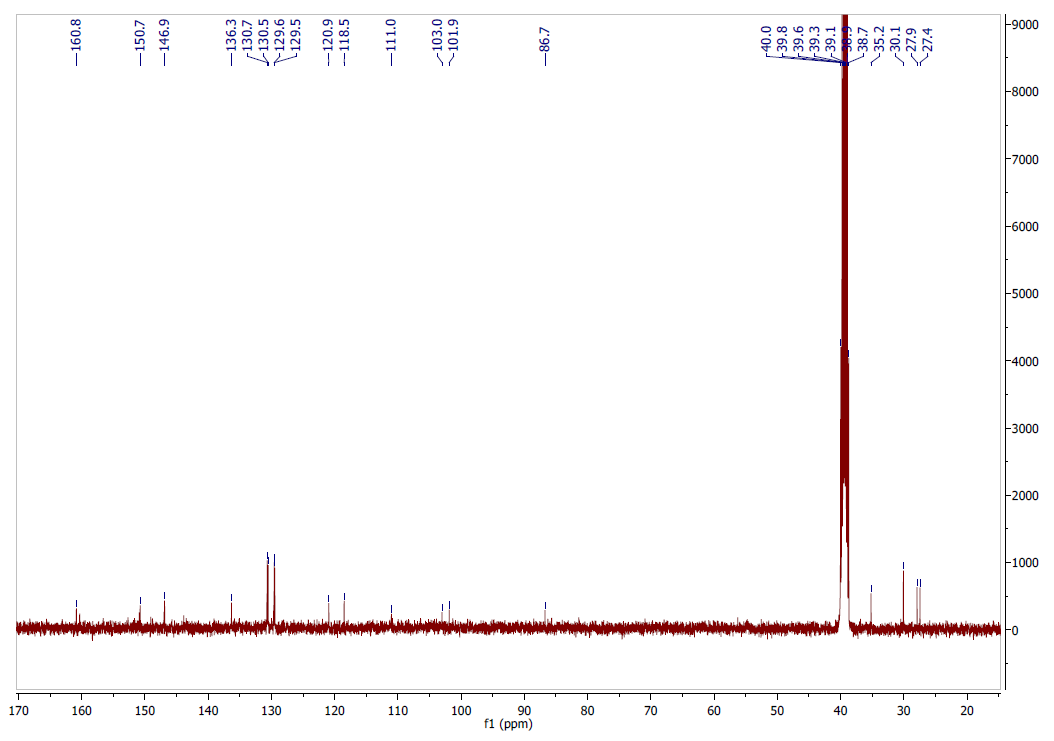
*

^13^C-NMR Spectrum of 4-(4-bromophenyl)-3,6,8-trimethyl-1,4,8,9-tetrahydro-5H-pyrazolo[4',3':5,6]pyrido[2,3-d]pyrimidine-5,7(6H)-dione

*
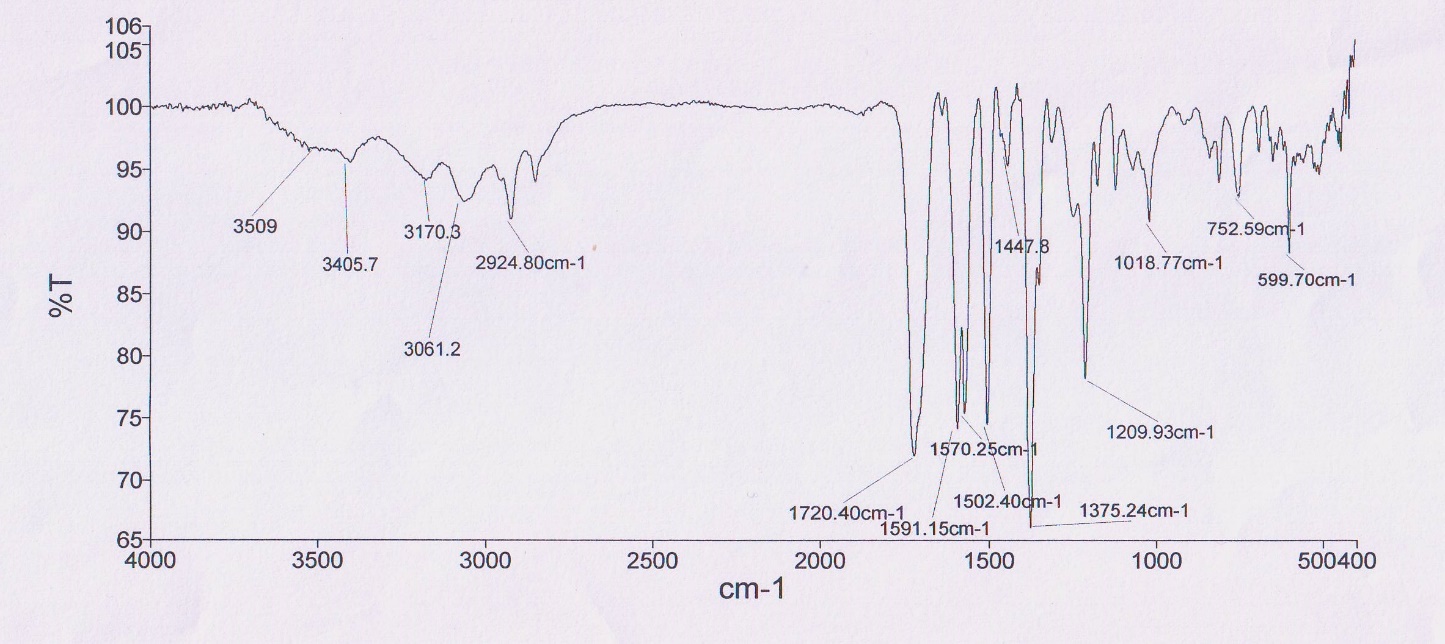
*

FT-IR Spectrum of 4,4',4''-(((1,3,5-triazine-2,4,6-triyl)tris(oxy))tris(benzene-4,1-diyl))tris(3-methyl-1-phenyl-1,8-dihydro-5H-pyrazolo[4',3':5,6]pyrido[2,3-d]pyrimidine-5,7(6H)-dione)

*
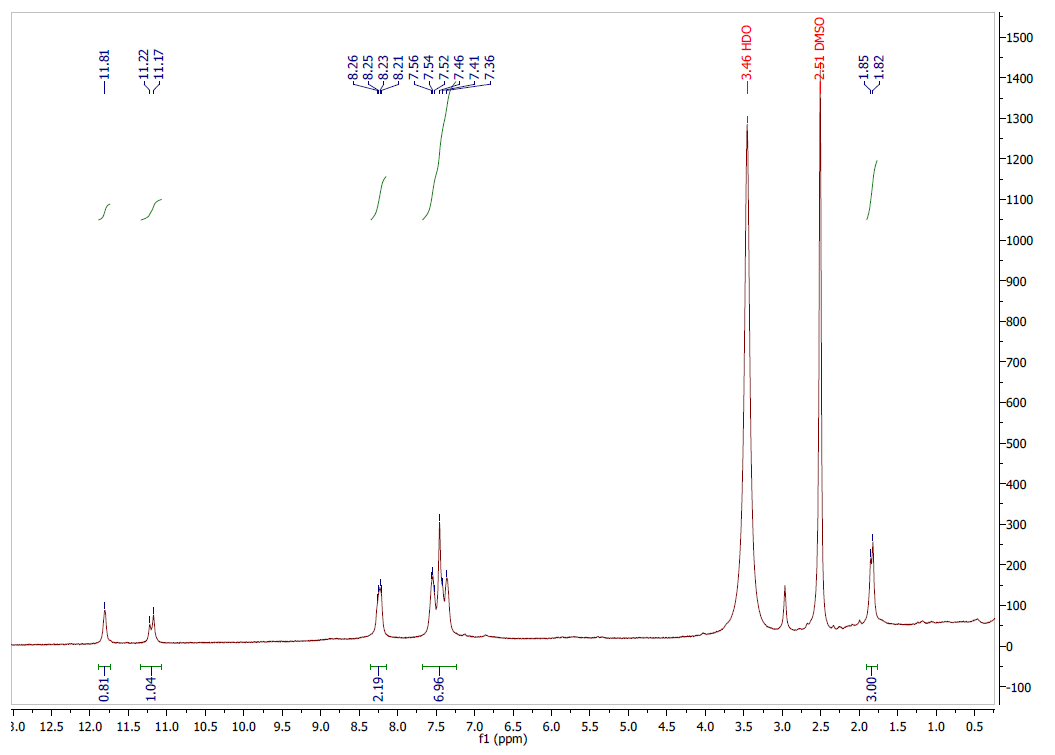
*

^1^H-NMR Spectrum of 4,4',4''-(((1,3,5-triazine-2,4,6-triyl)tris(oxy))tris(benzene-4,1-diyl))tris(3-methyl-1-phenyl-1,8-dihydro-5H-pyrazolo[4',3':5,6]pyrido[2,3-d]pyrimidine-5,7(6H)-dione)

*
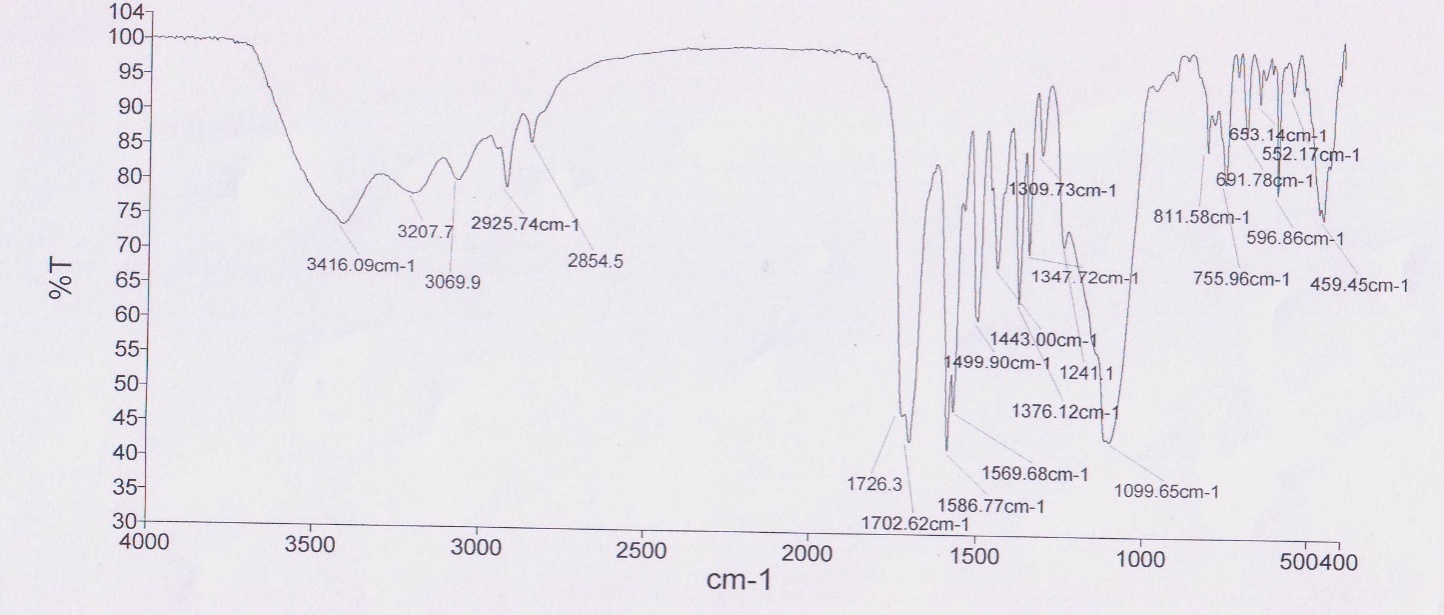
*

FT-IR Spectrum of 4,4'-(1,3-phenylene)bis(3-methyl-1-phenyl-1,8-dihydro-5H-pyrazolo[4',3':5,6]pyrido[2,3-d]pyrimidine-5,7(6H)-dione)

*
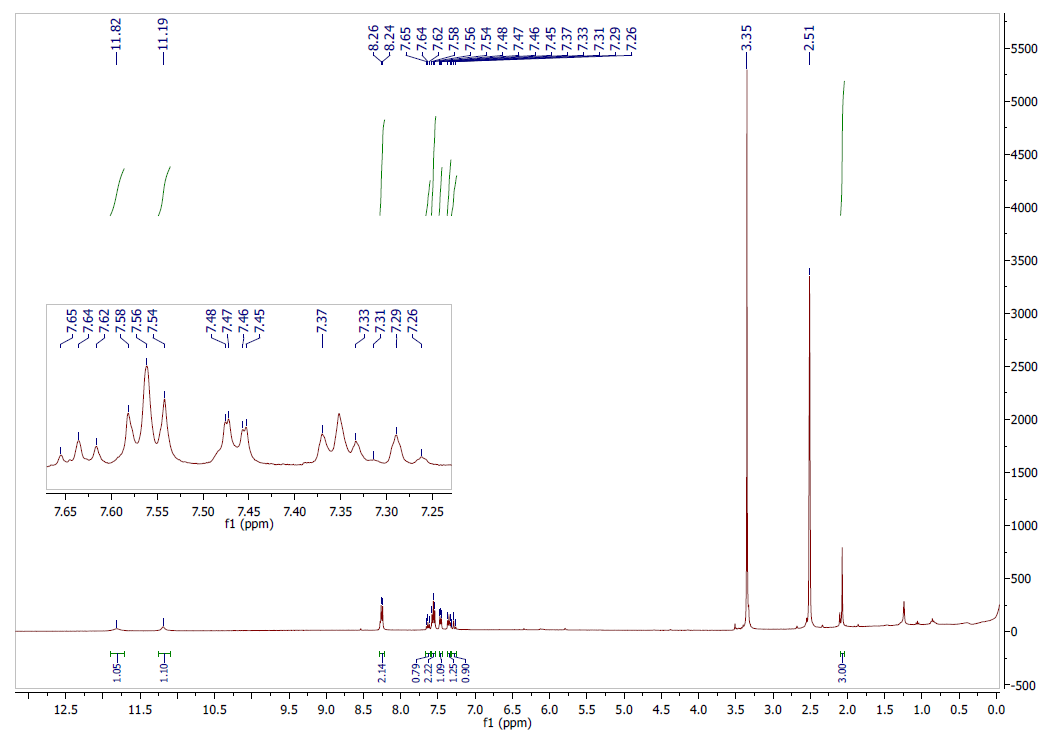
*

^1^H-NMR Spectrum of 4,4'-(1,3-phenylene)bis(3-methyl-1-phenyl-1,8-dihydro-5H-pyrazolo[4',3':5,6]pyrido[2,3-d]pyrimidine-5,7(6H)-dione)

*c
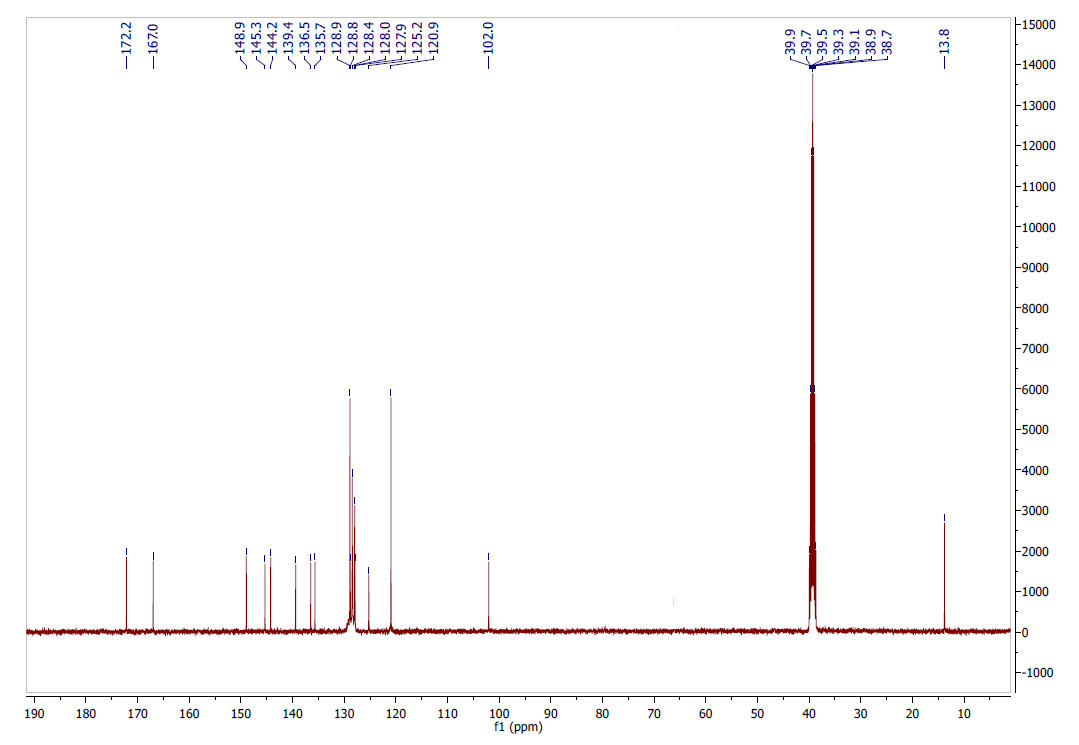
*

^13^C-NMR Spectrum of 4,4'-(1,3-phenylene)bis(3-methyl-1-phenyl-1,8-dihydro-5H-pyrazolo[4',3':5,6]pyrido[2,3-d]pyrimidine-5,7(6H)-dione)

^1H-NMR Spectrum of 4-(3,5-dimdthyl-1,7-diphenyl-1,4,7,8-tetrahydrodipyrazolo[3,4:4,3-e]pyridine-4-yl)-2-ethoxyphenol^ **^(W1)^**
